# Inhibition of SYK and cSrc kinases can protect bone and cartilage in preclinical models of osteoarthritis and rheumatoid arthritis

**DOI:** 10.1038/s41598-021-02568-6

**Published:** 2021-11-30

**Authors:** F. N. Novikov, M. V. Panova, I. Y. Titov, V. S. Stroylov, O. V. Stroganov, G. G. Chilov

**Affiliations:** 1 Zelinsky Institute of Organic Chemistry RAS, 47 Leninsky Prospect, Moscow, Russian Federation 119991; 2 National Research University Higher School of Economics (HSE), 20 Myasnitskaya Street, Moscow, Russian Federation 101000; 3 Molecular Technologies, LLC, Moscow, Russian Federation

**Keywords:** Rheumatic diseases, Drug discovery

## Abstract

The pathophysiology of osteoarthritis (OA) includes the destruction of subchondral bone tissue and inflammation of the synovium. Thus, an effective disease-modifying treatment should act on both of these pathogenetic components. It is known that cSrc kinase is involved in bone and cartilage remodeling, and SYK kinase is associated with the inflammatory component. Thus the aim of this study was to characterize the mechanism of action and efficacy of a small molecule multikinase inhibitor MT-SYK-03 targeting SYK and cSrc kinases among others in different in vitro and in vivo arthritis models. The selectivity of MT-SYK-03 kinase inhibition was assayed on a panel of 341 kinases. The compound was evaluated in a set of in vitro models of OA and in vivo OA and RA models: surgically-induced arthritis (SIA), monosodium iodoacetate-induced arthritis (MIA), collagen-induced arthritis (CIA), adjuvant-induced arthritis (AIA). MT-SYK-03 inhibited cSrc and SYK with IC_50_ of 14.2 and 23 nM respectively. Only five kinases were inhibited > 90% at 500 nM of MT-SYK-03. In in vitro OA models MT-SYK-03 reduced hypertrophic changes of chondrocytes, bone resorption, and inhibited SYK-mediated inflammatory signaling. MT-SYK-03 showed preferential distribution to joint and bone tissue (in rats) and revealed disease-modifying activity in vivo by halving the depth of cartilage erosion in rat SIA model, and increasing the pain threshold in rat MIA model. Chondroprotective and antiresorptive effects were shown in a monotherapy regime and in combination with methotrexate (MTX) in murine and rat CIA models; an immune-mediated inflammation in rat AIA model was decreased. The obtained preclinical data support inhibition of cSrc and SYK as a viable strategy for disease-modifying treatment of OA. A Phase 2 clinical study of MT-SYK-03 is to be started.

## Introduction

Osteoarthritis (OA) is the most common musculoskeletal system disease causing incapacitation with frequency comparable to that for colds and viral diseases^1–3^. It manifests as joint inflammation and loss of joint function up to disability. Existing OA treatment options are associated with side effects^4^ and are directed towards symptomatic relief of pain and inflammation^5^. Thus, development of true disease-modifying osteoarthritis drugs (DMOADs) is urgently needed.

The pathogenesis of OA is associated with aberrant bone and cartilage tissue metabolism and the concomitant chronic inflammation^6–8^. Despite the molecular pathogenesis of OA is not clearly understood compared to self-antigen driven rheumatoid arthritis (RA), another chronic inflammatory disease affecting joints (as well as other tissues and organs), the role of cytokines such as IL-1b IL-6 and TNFa appears to be important in sustaining both conditions^9,10^. Thus, inhibition of kinases downstream to the cytokines of interest might be a potential strategy for the OA treatment. One of the key kinases controlling cartilage and bone tissue remodeling is cSrc kinase^11–13^. It is involved in a number of signaling cascades that induce hypertrophic changes in chondrocytes and lead to decrease in anabolic rate and acceleration of catabolic processes in cartilage. In addition, cSrc kinase plays the key role in the formation of an actin ring, a unique element of the osteoclast cytoskeleton necessary for bone resorption^13^. Inhibition of cSrc kinase suppresses catabolism of cartilaginous tissue by inducing synthesis of essential articular cartilage components such as aggrecan and collagen II, reducing secretion of aggrecanases, and depressing osteoclast-mediated resorption of bone tissue^14^. Inflammation development during OA is associated with SYK kinase^15–17^, and leads to increased production of IL-1β and other inflammatory modulators which accelerate cartilage and bone tissue catabolism. SYK kinase inhibitors diminish the severity of inflammatory reaction^18,19^ and also decrease the rate of degenerative-dystrophic changes in the cartilaginous and bone tissues^20,21^.

Since both cSrc and SYK kinases are included in OA pathogenesis and are responsible for its catabolic and inflammatory effects, we hypothesize that their simultaneous inhibition can be used for developing disease-modifying treatment of OA.

Here we report on the preclinical characterization of MT-SYK-03, a novel selective orally available kinase inhibitor with cSrc and SYK kinases among its main targets. MT-SYK-03 provides a tool for testing the hypothesis that simultaneous cSrc and SYK inhibition may be effective for disease-modifying OA treatment. We study the selectivity profile of MT-SYK-03 on a broad kinase panel (341 kinases) and assess its inhibitory activity in cellular assays. Furthermore, we test for efficacy of MT-SYK-03 in animal models of OA. Because OA and RA involve at least a number of common cytokines as well as some common clinical features (such as destruction of the barrier between the bone and cartilage^22–24^ and formation of a pannus^25,26^), the animal models of RA also considered in the current study. We conclude that simultaneous inhibition of cSrc and SYK by MT-SYK-03 is efficient in suppressing bone and cartilage destruction, as well as inflammation in in vivo models and can be nominated for further evaluation in clinical setting.

## Materials and methods

All experiments were performed in accordance with relevant guidelines and regulations. The study was carried out in compliance with the ARRIVE guidelines.

### MT-SYK-03 preparation

MT-SYK-03 was synthesized according to the reported procedure^27^.

### In vitro experiments

#### SYK inhibition luminescent assay

Luminescent assay was performed at A. N. Bakh Institute of Biochemistry (Moscow, Russia). Recombinant 6xHis-SYK kinase (expressed in Sf9 cells) was obtained as described previously^28^. The reaction mixture containing the studied compound MT-SYK-03 or known SYK kinase inhibitor R406^19^ as a reference (1 nM–10 μM) and 6xHis-SYK (5 μL, 0.7 pM) was incubated for 30 min, then polyE4Y and ATP (up to 10 μM) were added. After incubation (20 min) Kinase-Glo reagent (Promega) was added. The luminescence was detected using the Fusion Universal Microplate Analyzer (PerkinElmer USA). The obtained IC_50_ values are given in Table S1, first column. Dose response curve of inhibitory activity of MT-SYK-03 against SYK kinase is presented in Fig. S1a.

#### SYK inhibition radiometric assay

Radiometric assay was performed at EMD Millipore Corporation (UK) by measuring the extent of substrate phosphorylation by 6xHis-SYK (expressed in Sf21 cells) using the standard protocol^29^. The reaction mixture containing polyE4Y (0.2 mg/mL), GST-labeled kinase (up to 2 nM), the studied compound (MT-SYK-03 or R406) and ^32^P ATP was incubated and spotted onto ion exchange filter; unbound phosphate was removed by extensive washing of filters in 0.75% phosphoric acid. The obtained IC_50_ values are given in Table S1.

#### cSrc inhibition and kinase selectivity profile assay

The activity of MT-SYK-03 against cSrc kinase and selectivity profile towards other 341 kinases was determined at Reaction Biology Corporation (Malvern, PA, USA) using the standard kinase assay protocol. The reaction mixture containing 0.1 mg/mL of substrate polyE4Y (Sigma, USA), 6xHis-SYK (0.1—0.7 pM), the studied compound MT-SYK-03 or reference R406 (500 nM) and ^33^P ATP (up to 10 μM, final specific activity 0.225–0.360 μCi) was incubated (120 min) and spotted onto ion exchange filter; unbound phosphate was removed by extensive washing of filters in phosphoric acid. Kinase activity data was expressed as the percent remaining kinase activity in test samples compared to vehicle (dimethyl sulfoxide) reactions based on the radioactivity of reaction products (see Table S2). Experiment was replicated twice. IC_50_ values for kinase listed in Table S3 (having less than 50% residual activity) were measured in 10-dose mode with threefold serial dilution starting at 10 μM and curve fits were obtained using Prism4 Software (GraphPad). Dose response curve of inhibitory activity of MT-SYK-03 against cSrc kinase is presented in Fig. S1b.

### *In-vitro* experiments

#### Osteoclast-mediated resorption of the mineralized matrix

The experiment was performed at Atlantic Bone Screen (France) and approved by Atlantic Bone Screen ethics committee. Human CD14 + monocytes were isolated from peripheral blood of healthy volunteers (informed consent was obtained from all subjects) using density gradient centrifugation (Ficoll-Hypaque) and magnetic cell sorting (MACS, MiltenyiBiotec). CD14 + monocytes (1.5 × 10^6^ cells/well) were cultured for three days in proliferation medium (αMEM/FCS-10% supplemented with 25 ng/mL M-CSF), inducing proliferation and expression of RANK. Secondly, the cells were differentiated into osteoclasts in differentiation medium supplemented with 100 ng/mL RANKL for 4 days. Osteoclasts were detached from the wells using accutase, the cell suspension was homogenized and cells (75 μL/well) were reseeded into: (a) a 96-well plate coated with a synthetic mineralized matrix for an evaluation of osteoclast resorption, or (b) a classic 96-well plastic culture plate, for an assessment of osteoclast number. 75 μL of differentiation medium containing M-CSF and RANKL (at a 2 × concentration) was added to each well. The cells were incubated for an hour and were cultured for 48 h in the fresh differentiation medium supplemented with MT-SYK-03 or vehicle (DMSO, 0.3%). All treatments were carried out in quadruple. Then the cell medium was removed, cells were lysed and the surface of the resorbed areas of the mineralized matrix was quantified in each well. TRAP staining was performed for cells in a classic plastic plate and the number of mature osteoclasts (TRAP-positive cells with three or more nuclei) was determined in each well. Reconstruction of images and osteoclast count was performed using Nikon NIS-D software. The obtained data are presented in Table S4.

#### Hypertrophic-like changes in IL-1β treated primary chondrocytes

The experiment was performed at Atlantic Bone Screen, France. To isolate primary chondrocytes, Sprague Dawley 3 weeks old rats were euthanized and their hind limbs were collected. Knee cartilage shavings were digested with collagenase in serum-free Dulbecco’s Modified Eagle’s Medium (DMEM). Once digested, the cell suspension was centrifuged and resuspended in DMEM containing 10% fetal bovine serum (FBS). Then the cells were plated on tissue culture plastic at a density of 10 000 cells∙cm^−2^ and were amplified in monolayer in culture medium (DMEM/FCS-10% supplemented with HEPES (25 mM)) until passage 1 and frozen at − 80 °C. Before study rat’s chondrocytes were seeded and cultured in monolayers in 12-well plates for 24 h. After that the cells were treated for three days with IL-1β (10 ng/mL) and MT-SYK-03; positive (no treatment at all), negative (only IL-1β) and vehicle (DMSO, 0.3% in cell medium) controls were also carried out in triplicate. At the end of the treatment, chondrocytes were lysed, and total RNA was purified using the NucleoSpin RNA II kit (Macherey Nagel). Total RNAs (2 μg) were retro-transcribed using M-MLV RT (Lifetechnologies) and obtained cDNAs (10 ng) were used for qPCR to determine steady-state levels of type II collagen and aggrecan mRNAs (levels of RPL19 and β-actin mRNAs were used as controls). Each reaction was set up as follows: 5 μL of iQ SYBR Green Supermix (Biorad, ref 1,708,882), 0.6 μL of forward primer (5 μM), and 0.6 μL of reverse primer (5 μM), 1.8 μL H_2_O, and 2 μL of cDNA. All qPCRs (72 qPCRs per marker) were run on a DNA Engine Thermal Cycler Chromo 4 in triplicates and analyzed using REST software^30^. The obtained data are presented in Table S5.

#### Detection of SYK activity in cells by Western blot analyses

Western blot was performed at A. N. Bakh Institute of Biochemistry, Russia. Human B-cell lymphoma cells obtained from Russian collection cell cultures (RCCC) were cultured in 5% CO2 humidified atmosphere at 37 °C in RPMI 1640 supplemented with 100 U mL^–1^ penicillin/streptomycin and 10% heat inactivated FBS. P3H3 cells (10^6^ cells in 1 ml of RPMI media with 5% FBS) were incubated with test compound or vehicle control (DMSO, 1%) for one hour at 37 °C in a 5% CO_2_-incubator. Then anti-IgM (1 μg/mL) was added to activate B-cell receptors and incubation continued for additional 15 min. After that the cells were washed with cooled PBS buffer and were lysed in 200 μL of ice-cold lysis buffer (62,5 mM Tris–HCl, pH 6.8, 2% w/v SDS, 10% glycerin, 50 mM DTT, 0,01% w/v BPB). Then lysates were sonicated for 20 s in ice, heated to 95–100 °C for 5 min and centrifuged for 5 min. 20 μL of obtained samples were electrophoresed on 10% SDS–polyacrylamide gel (gel blocks: 90 × 60 × 1.5 mm; current intensity: 30 mA, time: 2.5–3 h) and transferred to nitrocellulose membranes (BioRad). Western blotting was carried out according to the Amersham Pharmacia Biotech ECL Western blotting protocol. After being blocked with nonfat milk (Valio, Finland) for one hour and washed, the membranes were incubated for one hour with corresponding primary antibody (SYK, p-SYK (Tyr352), p-SYK (Tyr525/526), p38, Jnk, Erk1/2, p-p38 (Thr180/Tyr182), p-Erk1/2 (Thr202/Tyr204), p-JNK (Thr183/Tyr185), BTK, p-BTK (Tyr223), CD19 and p-CD19 (Tyr531) antibodies (Cell Signaling Technology, USA)) in solution with 5% BSA. The secondary antibodies were horseradish conjugated goat antirabbit antibodies.

#### B-cell activation in human whole blood

The whole blood assay was performed at N. N. Blokhin National Medical Research Center of Oncology (NMRCO), Russia and approved by NMRCO ethics committee. Informed consent was obtained from all subjects. Red blood cells were lysed by incubation with NH_4_Cl buffer (0.8% NH_4_Cl and 0.1% EDTA in distilled water) for 5 min at 37 °C and were precipitated by centrifugation (1500 rpm). The precipitates were resuspended in RPMI-1640 with 10% human serum and 2 mM L-glutamine, after that the cells were again precipitated by analogous centrifugation and resuspended in RPMI-1640. Prepared B-cells were activated by incubation with anti-human IgD antibody (or 80 nM PMA as a positive control) in presence of the test compounds (MT-SYK-03 or R406) or DMSO (5%) for 16 h at 37 °C in 5% CO_2_ humidified atmosphere. Blood was then stained directly with PE-conjugated anti-CD69 (dilution 1:10) and APC-conjugated anti-CD19 (dilution 1:20) antibodies for 20 min at 4 °C. After that cells were precipitated by centrifugation (1500 rpm, 4 °C) and washed twice with phosphate-buffered saline (PBS). The cells were resuspended in PBS containing 1% BSA and subjected to flow cytometry on BD FACS Canto II (BD Biosciences, USA). The number of active cells was estimated as a ratio of CD69-positive cells bearing a marker of CD19 cells, or as a mean fluorescence intensity (MFI) of CD69 + cells after gating on the CD19-positive cells. For dose–response curve see Fig. S2.

#### Inhibition of TNF-α secretion by activated monocytes

The assay was performed at A. N. Bakh Institute of Biochemistry, Russia. Human leukemia monocytic THP-1 cells obtained from Russian collection cell cultures (RCCC) were cultured in 5% CO2 humidified atmosphere at 37 °C in RPMI 1640 supplemented with 100 U mL^–1^ penicillin/streptomycin, 2% FBS and 20 μM β-mercaptoethanol. THP-1 cells were incubated in 5% CO_2_ atmosphere at 37 °C in RPMI 1640 medium. To induce differentiation the cells were treated with IFN-g for six days. 96-well culture plate coated with combined human IgG were incubated during night at 4 °C (or for 1 h at 37 °C). To evaluate baseline cell simulation F(ab’)_2_ was used as a negative control in part of wells. Unbounded antibodies were removed with sodium-phosphate buffer. A solution of test compound (MT-SYK-03 or) and differentiated cells in cultivating medium were added in each well. The cells were incubated at 37 °C after that the concentration of TNF-α in supernatant was measured fluorometrically using BMS223FF immunoanalytical kit by Bender MedSystems and IC_50_ value was computed. Each concentration was tested in triplicate; the experiment was replicated twice. For dose–response curve see Fig. S3.

### In vivo experiments

#### Pharmacokinetics of MT-SYK-03 after single oral administration to rats and rabbits

Experiments were performed at ChemPartner, Shanghai, China and approved by Institutional Ethics Committee (IEC) of ChemPartner. Male Wistar 10–12 weeks old rats with body weights of 185–220 g and male New Zealand rabbits with body weights of 2.0–2.3 kg were purchased from Shanghai SLAC Laboratory Animal Co., Ltd. MT-SYK-03 was administered to the three groups of rats (six rats per group) via oral gavage at 10 mL/kg concentration, and in rabbits 5 mL/kg concentration. Blood samples were kept on ice and centrifuged (2000 g, 4 ℃, 5 min) within 15 min post sampling.

#### Surgically induced meniscal tear OA in rats

Medial meniscal tear (MMT) model was performed at Pharmenterprises, Russia according to the published procedure^31^ and approved by Ethics Committee of Pharmenterprises. Male 4-month old Sprague-Dowley rats were anesthetized with Zoletil (Vibrac, France) and Xylazine (Interchemie werken "De Adelaar" BV, Netherlands) mixture. The medial collateral ligament was transected, and the medial meniscus was grasped with a hemostat and reflected proximally toward the femur. The meniscus was transected and then skin was closed with sutures. 60–90 min after surgery rats were subcutaneously injected with flexoprofen (VIC GROUP, Russia). Treatment groups (20 rats each) were intragastrically treated with MT-SYK-03 (100 and 500 mg/kg) or vehicle control (0.5% methylcellulose solution) 24 h before OA induction and then once daily for 21 day. Active control group was subcutaneously injected with zoledronic acid (0.1 mg/kg) 24 h before OA induction and then once every three days for 21 day. After 21 day animals were sacrificed; surgically operated right knee was removed and used for image analysis.

#### Monosodium iodoacetate induced osteoarthritis in rats

Monosodium iodoacetate (MIA) osteoarthritis model was performed at Pharmenterprises, Russia according to the known procedure^32^ and approved by Ethics Committee of Pharmenterprises. Male 10 week old Wistar rats were divided into eight groups: negative control (#1), positive control (#2) and six treatment groups (#3–8), ten rats in each group. Group #1 was left intact while rats in groups #2–8 were anesthetized on the day 0 with intraperitoneal injection of Zoletil and Xylazine mixture and given a single intra-articular injection of MIA through the infrapatellar ligament of the right knee. Rats were treated as follows: (#1, #2) 0.5% solution of methylcellulose in PBS (pH = 3) as vehicle control, (#3, #5) 100 mg/kg MT-SYK-03, (#4, #6) 500 mg/kg MT-SYK-03, (#7) 50 mg/kg nalgesin and (#8) 0.1 mg/kg zoledronic acid. Rats in groups #1–4 received treatment intragastrically QD, 25 and 1 h before the MIA injection, and then daily until the end of the study on the day 28. Rats in groups #5–6 received treatment intragastrically QD from the day 7 until the end of the study. Rats in group #7 were treated QD starting on the day 14 and until the end of the study. Finally, rats in group #8 were treated subcutaneously immediately after the MIA injection and then every third day until the study end. Disease progression was monitored by measuring mechanical hyperalgesia, mechanical allodynia and grip strength of hind paws on the days 14, 21, and 28, just before the treatment, and 1, 3, 6 h after it.

For experimental details on Collagen-Induced Arthritis and Adjuvant-Induced Arthritis please refer to the Supplementary Material.

## Results

### MT-SYK-03 is a potent and selective cSrc/SYK kinases inhibitor

**Figure 1:**
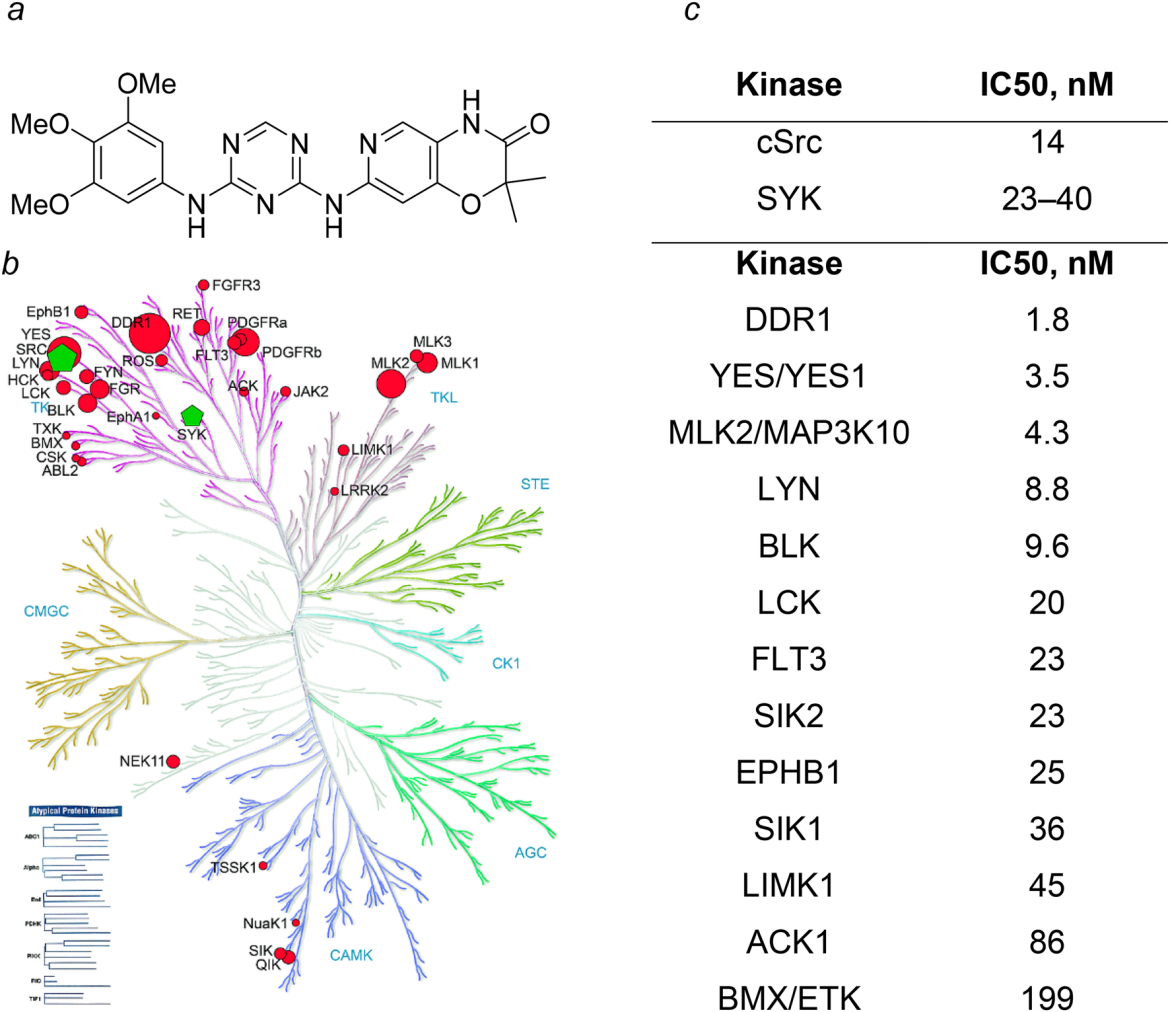
(**a**) Chemical structure of MT-SYK-03. (**b**) Kinase profiling of MT-SYK-03 (500 nM) against 343 kinases. Circle size on dendrogram is proportional to percentage of kinase residual activity: big circles, < 10% activity; small circles, < 20% activity. Green pentagons represent SYK and cSrc kinases. Kinase dendrogram background taken from *Manning *et al. Science **298** (2002). Reprinted with permission from AAAS. (**c**) IC_50_ values for cSrc and SYK kinases and other kinases with residual activity at 500 nM MT-SYK-03 below 20%.

MT-SYK-03 inhibited activities of cSrc and SYK kinases potently with IC_50_ values of 23–40 nM and 14 nM, correspondingly (Fig. 1). Of the other 341 protein kinases tested, at 500 nM 34 kinases were inhibited by more than 50%, 13 kinases—by more than 80% and only five kinases by more than 90% (DDR1, YES1, MAP3K10, LYN, BLK). Some of these kinases were found to be associated with osteoarthritis. DDR1 is expressed in chondrocytes, and is known to bind to type I collagen^33^; activates p38, ERK1/2, JNK MAP и PI3-AKT signal pathways^34,35^, as well as the Wnt/β-catenin one^36^. Osteoarthritis of temporomandibular joint was shown to develop spontaneously in Ddr1^−/−^ mice^37^. DDR1 was also recently shown to be involved in periostin-associated elevated MMP-13 expression via an AKT-Wnt/β-catenin pathway^38^. However, it is questionable that inhibition of Ddr1 kinase alone can have clinical effect on OA, because Ddr1 kinase is a common off-target of many approved kinase inhibitors, particularly approved Bcr-Abl inhibitors (imatinib, dasatinib, nilotinib, ponatinib), for which no data on efficacy in OA was reported. MAP3K10 is known to regulate cytokine expression, support homeostasis and regulatory T-cell functions in rheumatoid arthritis pathogenesis^39^; involved in Jnk and Jun mediated signaling pathways and has potential implications in osteoporosis pathogenesis^40^. Polymorphisms in BLK are potentially associated with increased risk of rheumatoid arthritis (RA)^41^.

A known SYK kinase inhibitor R406 was found to be less selective in the same assay: it inhibited 104 kinases by > 50%, 50 kinases by > 80% and 26 kinases by > 90%.

### MT-SYK-03 effectively suppresses osteoclast-mediated bone resorption and inhibits degradation of aggrecan and type II collagen in chondrocytes

**Figure 2:**
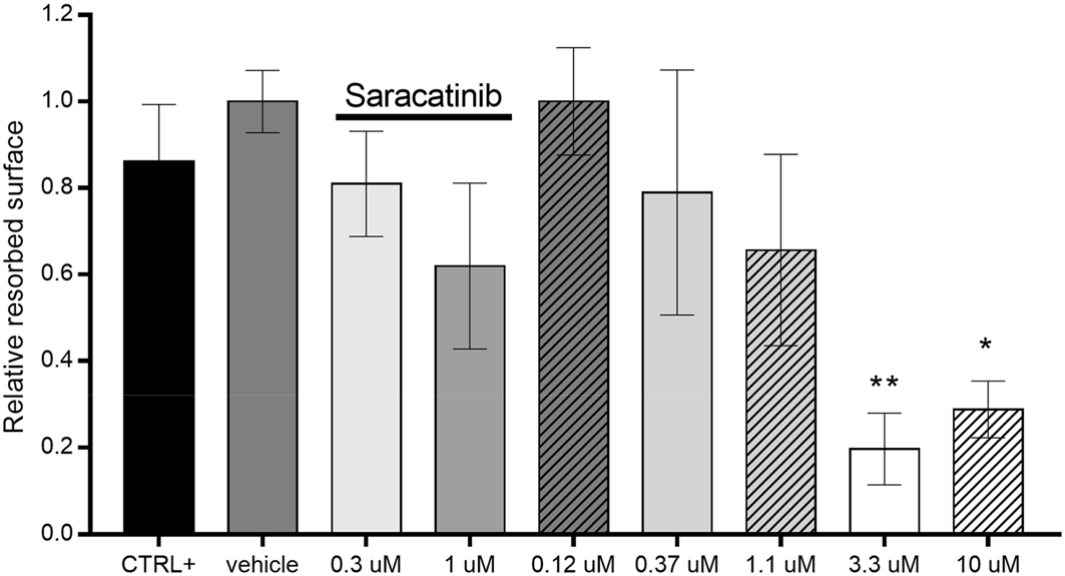
Relative resorbed surface after treatment with MT-SYK-03. The percentage of resorbed surface relative to the mean number of osteoclasts was calculated and compared to the vehicle control. Data are expressed as mean relative resorption ± SD (*p < 0.05; **p < 0.01) using Kruskal Wallis and Dunnet’s post test.

In the human osteoclast resorption assay MT-SYK-03 dose-dependently inhibited osteoclast resorption, reducing the relative resorbed surface more than threefold at 10 μM (Fig. 2). Anti-hypertrophic effect of MT-SYK-03 was assessed in chondrocytes treated with IL-1β where MT-SYK-03 increased the gene expression levels of aggrecan and type II collagen (Table S5). The increase in aggrecan expression was dose-dependent (Fig. S12).

### MT-SYK-03 inhibits SYK-mediated signaling

**Figure 3:**
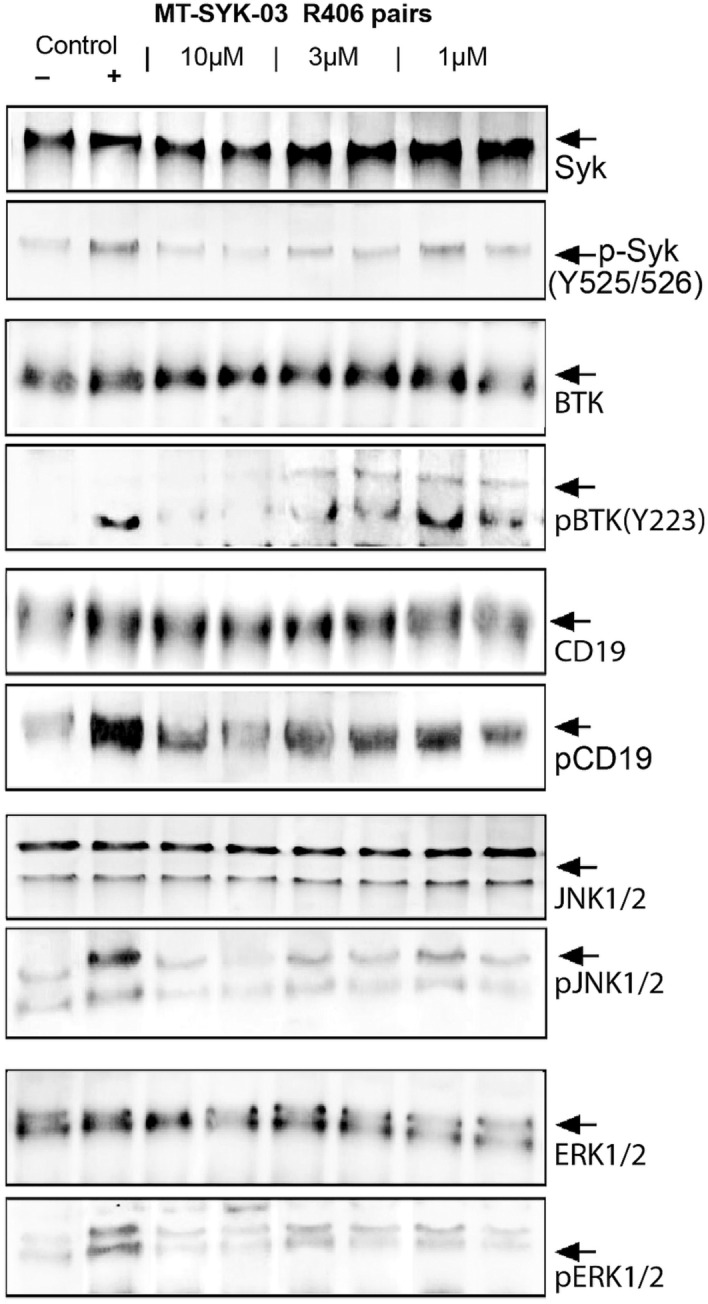
Effect of the MT-SYK-03 and a reference compound (R406, right hand side of each pair) on phosphorylation of the main modulators of the B-cell cascade development (tyrosine kinases Syk, BTK, as well as CD19 receptor) and effector kinases of mitogen/stress-activated signal cascade (JNK1/2 and ERK1/2). Cropped image from different blots is presented, as indicated by borders. Each section corresponds to a different blot.

Inhibition of SYK activity correlates well with suppression of B-cell activation. Treatment with MT-SYK-03 dose-dependently inhibited anti-IgM induced autophosphorylation of SYK kinase, phosphorylation of downstream B-cell signaling cascade components CD19 and BTK, as well as components of MAP cascade (ERK1/2 and JNK1/2) (Fig. 3).

SYK-kinase mediated inflammation induction can be achieved through B-cell receptor signaling and inflammatory cytokines production via FcγR signaling^42^. To confirm inhibition of SYK-dependent B-cells activation in human whole blood it was stimulated with anti-IgD (anti-BCR) or PMA to induce SYK-dependent or SYK-independent activation of B cells. Thus, MT-SYK-03 inhibited BCR-dependent (but not PMA-dependent) activation of B cells with IC_50_ value of 0.550 μM (Fig. S2).

As SYK kinase is also a key participant in FcγR-dependent signaling in monocytes, MT-SYK-03 effect was evaluated in the model of inhibition of cytokines production by differentiated monocytes. A dose-dependent inhibition of TNF-α release with IC_50_ value of 0.48 μM (Fig. S3) was shown. This effect of MT-SYK-03 may potentially translate into inhibition of TNF-α-induced inflammation and destruction of the joint cartilage during OA^43,44^.

### MT-SYK-03 pharmacokinetics profile and distribution in rats and rabbits

**Figure 4:**
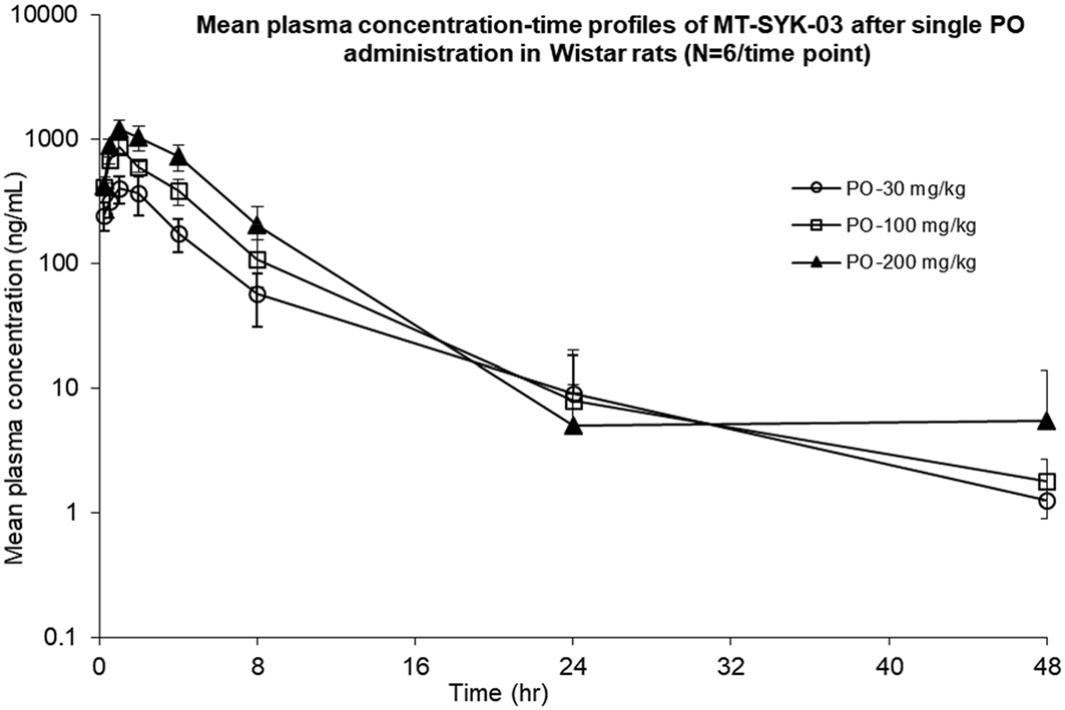
Mean plasma concentration–time profiles of MT-SYK-03 after PO administration of 30 mg/kg (circles), 100 mg/kg (squares) and 200 mg/kg (triangles) in male Wistar rats (N = 6/time point).

**Figure 5:**
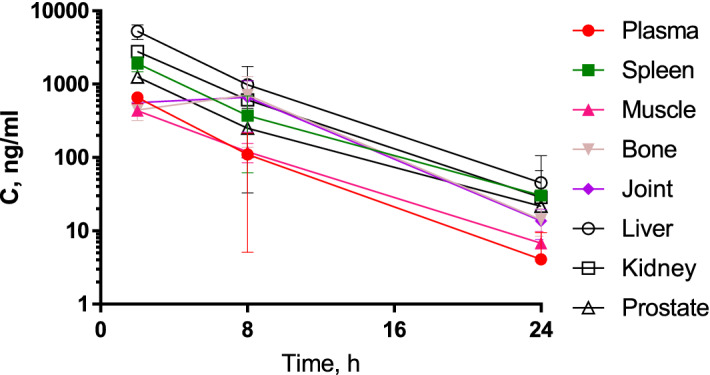
The dynamics of MT-SYK-03 concentration in various tissues after a single oral administration in rats.

The pharmacokinetic parameters after single oral administration of MT-SYK-03 are presented in Table 1 and Figs. 4 and 5. AUC and C_max_ were dose-proportional, T_max_ and t_1/2_ were dose-independent. The exposure to MT-SYK-03 was threefold higher in target tissues (bone and cartilage) than in plasma (Fig. 5). In rabbits the compound reaches higher plasma concentrations compared to rats.

**Table 1:** The main pharmacokinetic parameters after oral MT-SYK-03 administration in rats in three single doses and rabbits.

Parameters	Rats			Rabbits
	30 mg/kg	100 mg/kg	200 mg/kg	50 mg/kg
T_max_, h	1.5 ± 0.548	1.0 ± 0	1.33 ± 0.516	6.00 ± 2.19
*C*_max_, ng/mL	416 ± 114	864 ± 89.4	1276 ± 160	8573 ± 2893
*C*_max_, nM	684 ± 187	1424 ± 147	2099 ± 263	14,102 ± 4759
τ_1/2_, h	5.53 ± 2.38	4.06 ± 1.49	5.08 ± 2.39	7.41 ± 1.32
AUC_48_, h∙ng/mL	2339 ± 979	4260 ± 621	7274 ± 1490	119,493 ± 39,753
AUC_inf_, h∙ng/mL	2418 ± 945	4273 ± 608	7319 ± 1458	120,951 ± 39,331
Kel, h^−1^	0.16 ± 0.09	0.19 ± 0.08	0.17 ± 0.09	0.096 ± 0.015
MRT_24_, h	4.26 ± 0.57	4.12 ± 0.56	4.89 ± 1.63	9.2 ± 0.66

### MT-SYK-03 shows anti-resorptive effects on cartilage in surgically induced meniscal tear OA in rats

**Figure 6:**
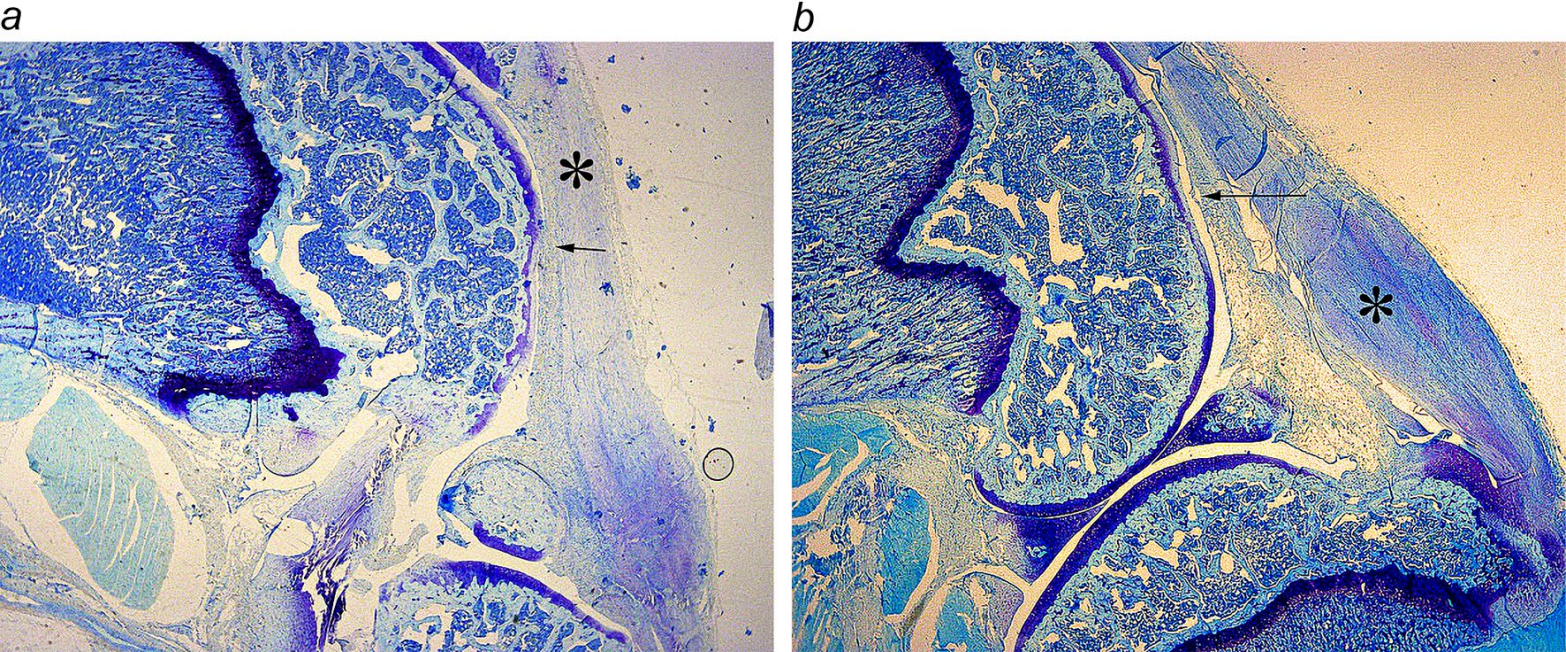
MT-SYK-03 protects against cartilage damage after medial meniscal tear in rats. Photomicrographs of sagittal cross Sects. (10 × magnification) of knee 21 days after medial meniscal tear surgery are shown for: (**a**) vehicle group; (**b**) treatment with MT-SYK-03 (500 mg/kg) group. Arrows indicate erosions, asterisks indicate thickening of the joint capsule.

The most commonly used rat medial meniscal tear (MMT) model demonstrates cartilage destruction and dynamic subchondral bone changes. Therefore, the cartilage- and bone-preserving activities of therapeutic agents can be evaluated in this model^45^. The model was carried out for 21 day, before gross joint damage becomes evident^46^: moderate to severe knee OA is already a late stage of the disease with advanced tissue changes that may not be amenable to any drug aimed at modifying the disease course^4^. It was shown that using disease-modifying OA therapy as a benchmark, that a positive result in rat MMT model would be predictive of efficacy in human OA^47^. We compared MT-SYK-03 with zoledronic acid. Joint damage was assessed histologically 3 weeks after surgery. Animals treated with vehicle were characterized by thickening of the joint capsule (fibrosis and hyperplasia of the synovial membrane) and erosion of the articular surfaces, down to the lower part of the cartilage (Fig. 6a). MT-SYK-03 therapy (500 mg/kg) halved lesion depth (from 68.5 ± 6.7% in vehicle group to 35.0 ± 3.0% in treatment group) (Fig. 6b). Treatment with zoledronic acid reduced lesion depth to a lesser extent. Lesion area was also smaller for both MT-SYK-03 and zoledronic acid treatment compared to negative control (Table 2).

**Table 2:** The area and surface of a lesion of cartilage tissue after meniscectomy on day 21.

Group	Lesion depth, %	Lesion area, %
Intact	0 ± 0	0 ± 0
Control	68.5 ± 6.7	37.0 ± 4.2
MT-SYK-03 (100 mg/kg)	72.5 ± 6.0	37.3 ± 3.3
MT-SYK-03 (500 mg/kg)	35.0 ± 3.0&	30.5 ± 2.8
zoledronic acid	50.5 ± 7.1	25.3 ± 2.2^&^

^&^Statistically significant (p < 0.05) compared with the control group.

### MT-SYK-03 is effective in mouse collagen-induced arthritis model

**Figure 7:**
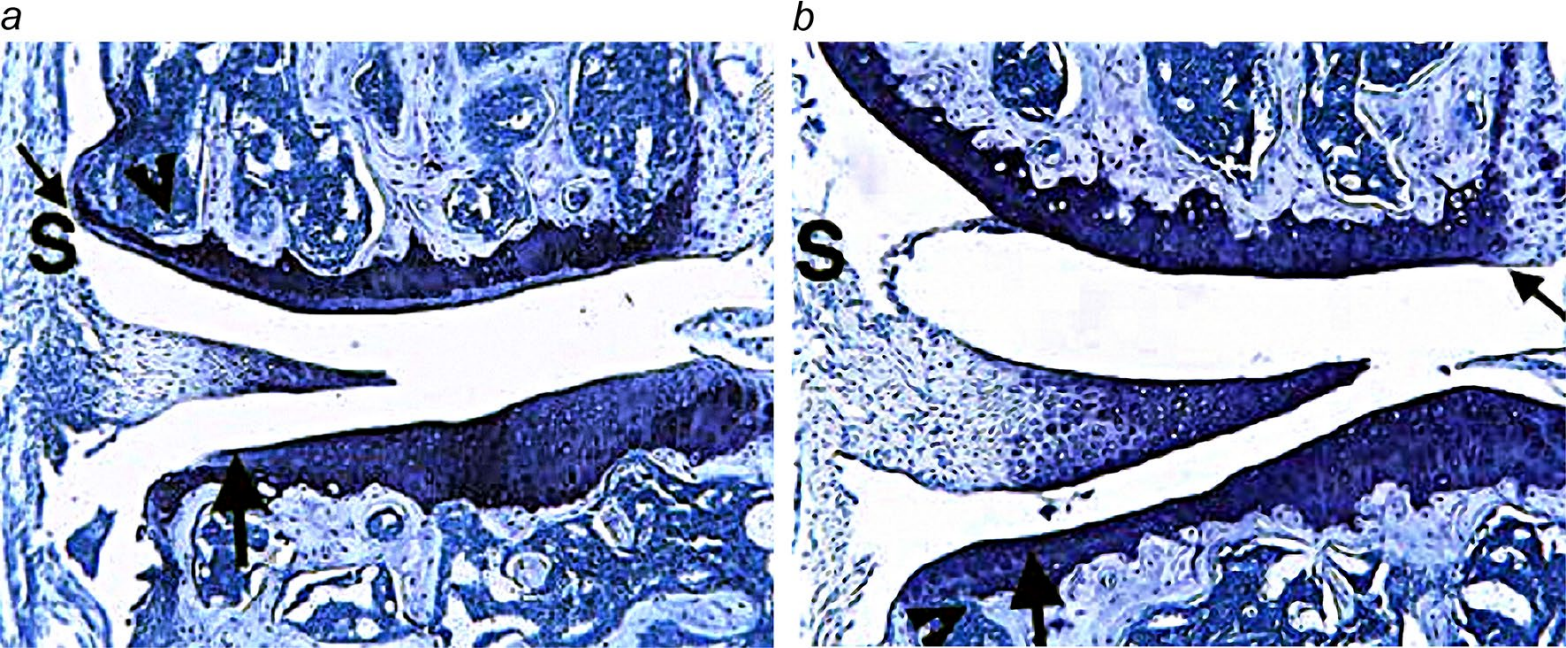
MT-SYK-03 alleviates inflammation and cartilage damage in murine CIA. Frontal section, 100 × magnification. *a*. The knee from a vehicle control animal reveals moderate inflammation (S) and marked cartilage damage (large arrow) with very minimal pannus (small arrow) and bone resorption (arrowhead). *b*. The knee from an arthritic animal treated with 103 mg/kg of MT-SYK-03 has very minimal inflammation (S) and minimal cartilage damage (large arrow).

Collagen-induced arthritis (CIA) model can be used to study all three components (cartilage and bone destruction as well as inflammation) of arthritis pathogenesis^48^. During the CIA development, paw inflammation was evident within 26 to 27 days after antigen boosting, at which time oral administration of MT-SYK-03 was initiated. MT-SYK-03 exhibited chondroprotective and antiresorptive effectiveness even at low dose (103 mg/kg): microphotographs of knees (Fig. 7) indicate decreasing of cartilage damage, bone resorption and inflammation in knees.

These results are consistent with decreased histopathology scores for knee and ankle joints for animal treated with MT-SYK-03. In case of a combination therapy with methotrexate (frequently used as reference^49,50^) these results were statistically significant (Fig. S16). MT-SYK-03 slightly decreased periosteal bone width of paw and knee joints. This effect was statistically significant when MT-SYK-03 was used in combination with MTX (Fig. S17, Table S18).

**Figure 8:**
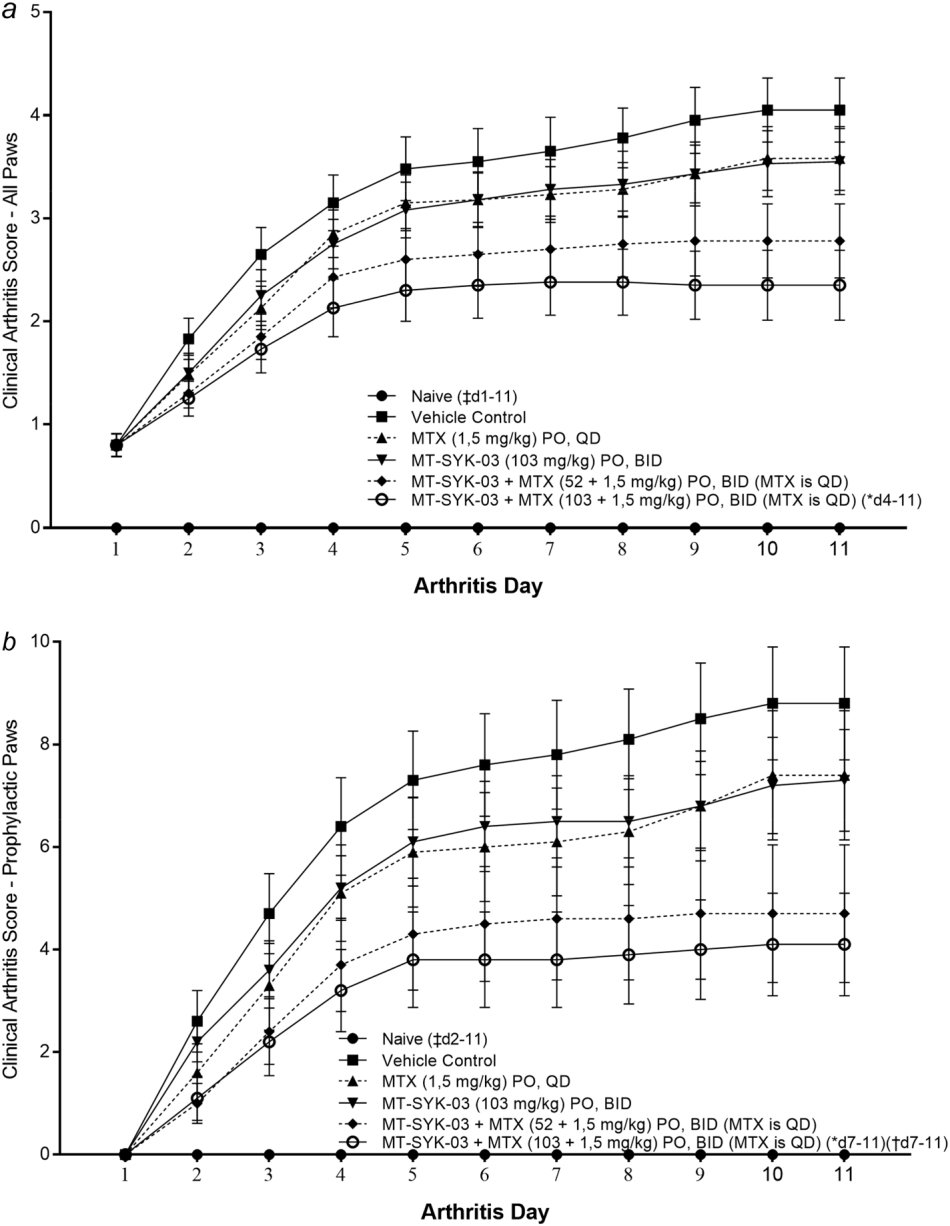
Effects of oral administration of MT-SYK-03 alone or in combination with MTX in murine CIA model. The clinical arthritis score, mean ± SE (n = 10 for vehicle and treatment groups and 4 for naive group) for (**a**) all paws and (**b**) non-involved (prophylactic) paws for six groups of animals: Scores measured severity of inflammation from 0 (no inflammation) to 5 (severe inflammation). *p < 0.05 ANOVA to Vehicle Control, ^†^p < 0.05 ANOVA to MTX (1.5 mg/kg), ^‡^p ≤ 0.05 *t*-test to Vehicle Control.

As for anti-inflammatory activity, MT-SYK-03 at 103 mg/kg reduced the severity of inflammation (Fig. 8a). The effect was larger when combined with MTX (1.5 mg/kg). Reduction of inflammation was more pronounced if measured by only non-involved paws (Fig. 8b), suggesting the autoimmune contribution.

### MT-SYK-03 is effective in rat collagen-induced arthritis model

**Figure 9:**
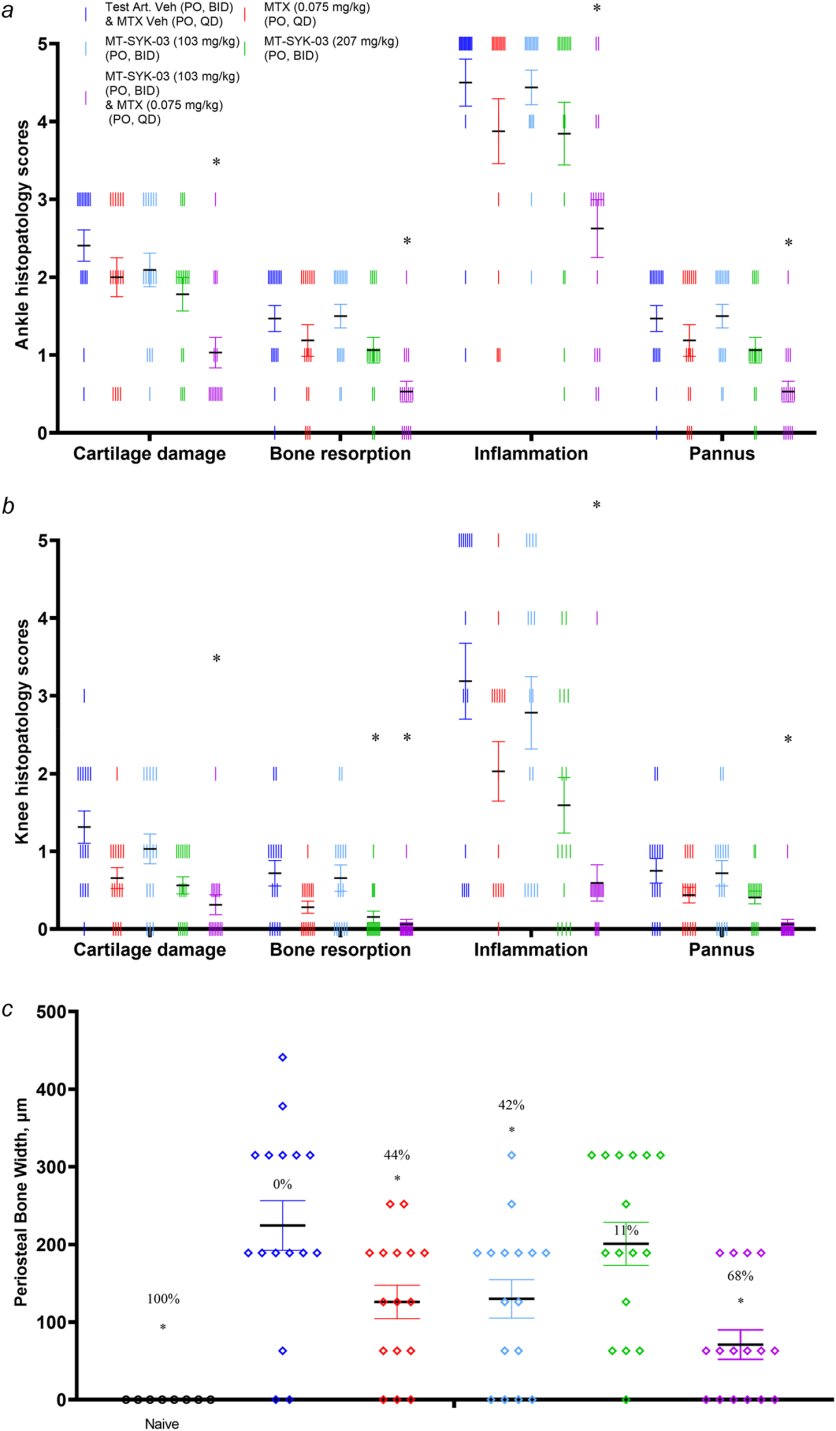
Effects of oral administration of MT-SYK-03 alone or in combination with MTX in 16 days rat semi-established CIA on inflammation, pannus, cartilage damage and bone resorption in ankles (**a**) and knees (**b**) and periosteal bone width in knee joints (**c**). Individual data, Mean ± SE (n = 8 for treatment groups and 4 for naive group, 2 paws were assayed for each animal). *p < 0.05 ANOVA to Vehicle Control.

In the rat CIA model, treatment was initiated on study day 6, 5–6 days before the ankle inflammation began (Fig. S9). As pharmacokinetics studies in rats showed fast metabolism of MT-SYK-03 with τ_1/2_ value of 5.08 ± 2.39 h at 200 mg/kg dose (Table 1), higher doses of MT-SYK-03 (compared to murine CIA model) were used in this experiment. Blinded histopathology scores for cartilage damage and bone resorption (Fig. 9, see SI for scoring details) indicated that MT-SYK-03 demonstrated chondroprotective and antiresorptive effects, although statistical significance was reached only for the knee bone resorption. MTX alone did not show statistical significance for any parameter, however its combination with 103 mg/kg of MT-SYK-03 was found to have statistically significant effects on all disease progression parameters, both at knees and ankles (Fig. 9).

Measurements of periosteal bone width (Fig. 9c) showed that high-dose MT-SYK-03 (207 mg/kg) and MTX similarly decrease periosteal bone formation by ~ 43%, while combination of the latter with low dose of MT-SYK-03 resulted in stronger decrease of ~ 68%.

Histopathology scores of inflammation and pannus formation were also smaller for MT-SYK-03 (207 mg/kg). For a combination of 103 mg/kg MT-SYK-03 with MTX the changes were statistically significant. As opposed to treatment with MT-SYK-03 or MTX alone, the combination yielded the largest effect (10% diameter reduction) which stayed statistically significant from day 11 up to the end of the study, and resulted in statistically significant 56% paw weight reduction at the study end (Figs. S9, S10). Relative weights of thymus, liver and spleen were statistically identical across all groups (Fig. S11), indicating absence of toxic effects of MT-SYK-03 treatment.

### MT-SYK-03 shows anti-inflammatory effect in adjuvant-induced OA in rats

Primary and secondary immunological reactions to the adjuvant injection were assessed by measuring volumes of the involved (CFA-injected) and non-involved paws, respectively, on days 0, 14, 18, 21, 25, and 28. MT-SYK-03 in doses 103 and 207 mg/kg and high-dose MTX decreased the volume of non-involved paws on the day 28, while diclofenac and low-dose MTX showed no statistically significant effect compared to control animals (Table S26). Combinations of MTX with MT-SYK-03 have shown statistically significant reduction of volume of paws on the days 21, 25 and 28.

Considering the treated paws (Table S22), only MTX and its combinations with MT-SYK-03 reached statistically significant reductions of paw volume, but only by day 28. As for toxicological profile, MT-SYK-03 demonstrated the best survival rate (0% rats died) compared to sodium diclofenac (20% rats died) and MTX (20% and 70% rats died after 0.2 and 0.5 mg/kg administration, respectively, Table S23). All rats died from bleeding ulcerative lesions of the stomach and intestines, confirming known side effects of diclofenac and MTX and showing lack of this fall-out for MT-SYK-03.

### MT-SYK-03 alleviates pain in MIA-induced OA in rats

The development of pain in OA is correlated with joint destruction but it can also be induced by more subtle changes or biochemical events without structural correlates^46^. Injection of sodium mono-iodoacetate (MIA) into rat joint is referred as a chronic pain model caused by cartilage degeneration^46,51^. The initial period of knee swelling in the MIA model associated with a transient synovial inflammation may correlate with early synovial inflammation in clinical setting that predicts development of OA in the knee^46^. Pain syndrome was assessed using three criteria: hind paws grip strength, mechanical allodynia and mechanical hyperalgesia. MT-SYK-03 in all doses showed trend toward restoration of hind paws grip strength; nalgesin and zoledronic acid, in turn, showed stable statistically significant improvements, but only on day 28 (Table S24). According to mechanical hyperalgesia measurements, prophylactic administration of MT-SYK-03 led to statistically significant increase of pain thresholds for both doses from day 14. In the therapeutic treatment regimen, a statistically significant improvement appeared only on day 28 for higher dose of MT-SYK-03. Both zoledronic acid and nalgesin demonstrated positive effects, but they were statistically significant only on days 21 and 28. Thus, prophylactic treatment with MT-SYK-03 has shown the best timing of onset (Table S25). According to mechanical allodynia measurements (Table S27), best efficacy of MT-SYK-03 was reached with prophylactic treatment regimen: on day 14 pain threshold was elevated in all measurements, on days 21 and 28—one and three hours after treatment. In the treatment regimen, positive effects of MT-SYK-03 administration became statistically significant on day 28 and only in the higher dose – 500 mg/kg. Zoledronic acid and nalgesin increased the pain threshold, but their therapeutic effects were outplayed by those of MT-SYK-03 in the prophylactic treatment regime.

## Discussion

The disease-modifying osteoarthritis drugs (DMOADs) being developed currently target only one of three main components of OA: articular cartilage damage, subchondral bone resorption, or synovium inflammation^4,52,53^. Despite the evidence of multiple phenotypes of OA, the data on how distinct phenotypes respond to current treatment is lacking^4^. In our study we tested the hypothesis whether selective inhibition of cSrc and SYK kinases by MT-SYK-03 can potentially affect all three pathogenetic components of OA.

MT-SYK-03 selectivity profile was compared with that of a known SYK kinase inhibitor R406 (tamatinib). The narrow therapeutic window for R406 was responsible for its discontinuation in rheumatoid arthritis^54^. A binding assay to profile MT-SYK-03 against 341 other protein kinases demonstrated that the molecule is highly selective. Complete selectivity toward cSrc is hard to reach due to its similarity to the related kinases^55^, but MT-SYK-03 is far more selective compared to R406 toward some of them (Lyn, Fyn, Hck, PDGFR, Lck, Fgr, Blk, Flt4, Ret, EphA1) and moderately inhibits (> 54% residual activity at 500 nM) the rest (BCR-Abl, c-Kit, EphA2, EphA3, EphA4, EphA5, EphA6, EphA7, EphA8, EphB2, EphB3, EphB4, EGFR, FGFR1-4, TIE2, Brk, Frk). Importantly, MT-SYK-03 did not inhibit FLT1 and KDR kinases inhibition of which was associated with hypertension^56^, diarrhea, nausea and increased transaminase^57^. Also, despite the high PO doses used in in vivo experiments, the average plasma concentration after administration in rats is relatively low and lies in 200–700 nM range thereby indicating the achievement of kinase selectivity in vivo. Interestingly, while plasma PK parameters confirmed achievability of effective concentrations in vivo, the drug’s exposure to bone and cartilage tissues was found to be 3 times higher. Taken together with promising preclinical toxicology and a relatively selective kinase inhibition profile, there is a possibility of favorable safety of drug candidate in a clinical setting, which was recently confirmed by Phase 1 clinical study of MT-SYK-03 in healthy volunteers [to be published].

In vitro and in vivo studies were conducted to support cSrc and SYK-mediated biological action of MT-SYK-03 and to check its effectivity in alleviation of OA symptoms: cartilage degradation, bone remodeling, inflammation, and pain. Antiresorptive and chondroprotective effects of MT-SYK-03 were demonstrated in osteoclast-mediated bone resorption and chondrocyte hypertrophic-like changes models in vitro and in surgically induced meniscal tear and collagen-induced arthritis models in vivo. Although it was hypothesized earlier that such effects may be associated with inhibiting other kinases (for example, osteoclastogenesis suppression was explained by MEK1, MAPKAPK2, PI3K or PKA inhibition^12^), MT-SYK-03 did not inhibit any from this list.

It should also be noted that some of in vivo models used to characterize MT-SYK-03, namely AIA and CIA, are well recognized as rheumatoid arthritis models and were used in this study to study features common for both RA and OA: inflamation^58^, immunity^59,60^, subchondral bone resorption^22–24^, and pannus^25,26^.

The role of SYK inhibition in the anti-inflammatory properties of MT-SYK-03 was tested in in vitro and in vivo models. In AIA model MT-SYK-03 was compared with methotrexate and diclofenac. Despite not being approved as an OA therapy, methotrexate efficacy was recently investigated in this indication^61^; some sources report its case studies in knee OA and erosive OA^62–64^. Administration of methotrexate and diclofenac was associated with severe adverse effects including lethality. On the contrary, MT-SYK-03 demonstrated anti-inflammatory activity without such adverse effects. MT-SYK-03 combined with MTX demonstrated efficacy in a setting where MTX alone was not effective^65,66^.

Current results demonstrate that MT-SYK-03 is a kinase inhibitor with a relatively selective profile and cSrc and SYK kinases among the main targets associated with OA. To our knowledge, this is the first compound which acts simultaneously on bone and cartilage metabolism and inflammation in the synovium and demonstrates disease-modifying ability (zoledronic acid that is approved only as an antiresorptive therapy showed low disease-modifying ability^4^; combined with severe adverse effects when administered intravenously^67^). The phase 2 clinical study of MT-SYK-03 in patients with painful OA is being planned.

## Supplementary Information

Supplementary Information.
