# Author Correction: Inhibition of SYK and cSrc kinases can protect bone and cartilage in preclinical models of osteoarthritis and rheumatoid arthritis

**DOI:** 10.1038/s41598-022-21911-z

**Published:** 2022-10-11

**Authors:** F. N. Novikov, M. V. Panova, I. Y. Titov, V. S. Stroylov, O. V. Stroganov, G. G. Chilov

**Affiliations:** 1 Zelinsky Institute of Organic Chemistry RAS, 47 Leninsky Prospect, Moscow, Russian Federation 119991; 2 National Research University Higher School of Economics (HSE), 20 Myasnitskaya Street, Moscow, Russian Federation 101000; 3 Molecular Technologies, LLC, Moscow, Russian Federation

Correction to: *Scientific Reports* 10.1038/s41598-021-02568-6, published online 30 November 2021

In the original version of this Article F. N. Novikov was incorrectly affiliated with ‘Molecular Technologies, LLC, Moscow, Russian Federation’. The correct affiliations are listed below.

Zelinsky Institute of Organic Chemistry RAS, 47 Leninsky Prospect, Moscow, Russian Federation, 119991

National Research University Higher School of Economics (HSE), 20 Myasnitskaya Street, Moscow, Russian Federation, 101000

The original Article and accompanying Supplementary Information file have been corrected.

