# Supplementary material for: Inhibition of SYK and cSrc kinases can protect bone and cartilage in preclinical models of osteoarthritis and rheumatoid arthritis
Source: Sci Rep. 2021 Nov 30;11:23120. doi: 10.1038/s41598-021-02568-6 (PMC8632988; doi:10.1038/s41598-021-02568-6)
Supplement: Supplementary file 1 — Supplementary Information. [file 41598_2021_2568_MOESM1_ESM.docx]

Supporting Information

Inhibition of SYK and cSrc kinases can protect bone and cartilage in preclinical models of osteoarthritis and rheumatoid arthritis.

Novikov FN,^1,2^ Panova MV,^1^ Titov IY,^1,2*^ Stroylov VS,^1,2*^ Stroganov OV,^2,3^ Chilov GG^2,3^

^1^ Zelinsky Institute of Organic Chemistry RAS, Moscow, Russian Federation;

^2^ National Research University Higher School of Economics (HSE), 20 Myasnitskaya Street, 101000, Moscow, Russian Federation;

^3^ Molecular Technologies, LLC, Moscow, Russia;

**Collagen-Induced Arthritis Model in Mice.**

Collagen-induced arthritis (CIA) model was performed at Bolder BioPATH, USA. Study design and animal usage was approved by Bolder BioPATH’s Institutional Animal Care and Use Committee. CIA was induced in 6–7 week old male DBA/1 mice with body weight of 15–26 g (Harlan, Inc) by intradermal injection of Freund’s Complete Adjuvant (Difco) containing bovine type II collagen (Bolder BioPATH) on day 0 and again on day 21 according to the standard protocol. Establishment of disease was evidenced by swelling of at least one paw on days 26–27; clinical arthritis scores were provided. After that mice were randomized into six groups: a control group of four, vehicle group and four treatment groups of ten mice each. Mice in treatment groups were treated orally during 11 days as follows: (1) MT-SYK-03 103 mg/kg BID, (2) MT-SYK-03 52 mg/kg BID and MTX 1.5 mg/kg QD, (3) MT-SYK-03 103 mg/kg BID and MTX 1.5 mg/kg, QD, (4) MTX 1.5 mg/kg QD. Each paw was scored daily for inflammation. Therapeutic and prophylactic paws were considered. At study termination mice were euthanized by CO2 inhalation followed by cervical dislocation. Fore paws, hind paws, and knees were removed for microscopy.

**Collagen-Induced Semi-Established Arthritis Model in Rats.**

Rat CIA model was performed was performed at Pharmenterprises, Russia and approved by Ethics Committee of Pharmenterprises. Female Lewis rats with body weight of 164–183 g (Charles River Laboratories, Inc., Wilmington, MA) were injected with porcine type II collagen (Chondrex) emulsified with Freund’s Incomplete Adjuvant (Sigma Aldrich) at two sites at the base of the tail according to standard protocol. On day 7, the rats were boosted with a second subcutaneous injection. On day 6 of the study, rats were randomized by body weight into six groups: a control group of four, and five treatment groups of eight. Treatment groups were orally treated from day 6 to day 16 as follows: (1) 207 mg/kg MT-SYK-03 BID and Veh2 QD, (2) 103 mg/kg MT-SYK-03 BID and Veh2 QD, (3) Veh1 BID and 0.075 mg/kg methotrexate (MTX, a solution in Veh2) QD, (4) 103 mg/kg MT-SYK-03 BID and 0.075 mg/kg MTX QD, (5) Veh1 BID and Veh2 QD. Disease progression was monitored via daily ankle thickness measurements. At study termination on day 16 rats were anesthetized with Isoflurane, bled by vacutainer through the descending aorta to exsanguinate and then euthanized for tissue collection. Hind paws were transected at the level of the medial and lateral malleolus, weighed, and collected for microscopy.

**Adjuvant-Induced Arthritis in Rats.**

Rat adjuvant-induced arthritis (AIA) model was performed at Pharmenterprises, Russia as described previously^3^ and approved by Ethics Committee of Pharmenterprises. Male 10 week old mongrel rats were divided into eleven groups: negative control (#1), positive control (#2) and nine treatment groups (#3–11), ten mice in each group. Group #1 was left intact, while subplantar injection of 0.1 mL of Complete Freund’s adjuvant (CFA, Sigma) was made on day 0 at right paws of rats in groups #2–8. Rats were intragastrically treated daily from the day 14 until the end of the study on the day 28 as follows: (#1, #2) 0.5% solution of methylcellulose in PBS (pH = 3) QD as vehicle control, (#3) 8 mg/kg diclofenac QD, (#4) 0.2 mg/kg MTX QD (#5) 0.5 mg/kg MTX QD, (#6, #7) 52 or 103 mg/kg MT-SYK-03 BID, respectively, (#8) 207 mg/kg MT-SYK-03 QD, (#9, #10, #11) combination of MTX (0.2 mg/kg QD) and MT-SYK-03 (52 mg/kg BID, 103 mg/kg BID, or 207 mg/kg QD, correspondingly). Disease progression was monitored by measuring volumes of the right (involved) and left (non-involved) paws on days 0, 14, 18, 21, 25, and 28.

Clinical arthritis scores in mice collagen-induced arthritis model

Mice were weighed on arthritis days 1, 3, 5, 7, 9, and 11 (prior to necropsy). Daily clinical scores were given for each of the paws (right front, left front, right rear, left rear) on days 1–11 using the following criteria:

0 – Normal.

1 – One hind or fore paw joint affected or minimal diffuse erythema and swelling.

2 – Two hind or fore paw joints affected or mild diffuse erythema and swelling.

3 – Three hind or fore paw joints affected or moderate diffuse erythema and swelling.

4 – Four hind or fore paw joints affected or marked diffuse erythema and swelling.

5 – Entire paw affected, severe diffuse erythema and severe swelling, unable to flex digits.

Morphologic pathology methods and scores in mice collagen-induced arthritis model

After 1–2 days in fixative and 4–5 days in 5% formic acid for decalcification, tissues were trimmed, processed for paraffin embedding, sectioned at 8 µm and stained with toluidine blue. Hind paws and knees were embedded and sectioned in the frontal plane. Two joints from each animal were processed for histopathology evaluation. The joints were then scored by two independent readers as indicated below. When scoring, severity of changes as well as number of individual joints affected were considered. When only 1–3 joints of the paws or ankles out of a possibility of numerous metacarpal/metatarsal/digit or tarsal/tibiotarsal joints were affected, an arbitrary assignment of a maximum score of 1, 2 or 3 for parameters below was given depending on severity of changes. If more than 3 joints were involved, the criteria below were applied to the most severely affected/majority of joints.

*Inflammation scores*

0 – Normal.

0.5 – Very minimal, affects only 1 joint or minimal multifocal periarticular infiltration of inflammatory cells.

1 – Minimal infiltration of inflammatory cells in synovium and periarticular tissue of affected joints.

2 – Mild infiltration of inflammatory cells. If referring to paws, generally restricted to affected joints (1–3 affected).

3 – Moderate infiltration with moderate edema. If referring to paws, restricted to affected joints, generally 3–4 joints and the wrist or ankle.

4 – Marked infiltration affecting most areas with marked edema, 1 or 2 unaffected joints may be present.

5 – Severe diffuse infiltration with severe edema affecting all joints (to some extent) and periarticular tissues.

*Pannus formation scores*

0 – Normal.

0.5 – Very minimal, affects only 1 joint at marginal zone.

1 – Minimal infiltration of pannus in cartilage and subchondral bone, marginal zones of two or more joints.

2 – Mild infiltration with marginal zone destruction of hard tissue in affected joints.

3 – Moderate infiltration with moderate hard tissue destruction in affected joints.

4 – Marked infiltration with marked destruction of joint architecture, affecting most joints.

5 – Severe infiltration associated with total or near total destruction of joint architecture, affects all joints.

*Cartilage damage scores*

0 – Normal.

0.5 – Very minimal (Affects marginal zones only of 1 to several joints.)

1 – Minimal (Generally minimal to mild loss of toluidine blue staining with no obvious chondrocyte loss or collagen disruption in affected joints.)

2 – Mild (Generally mild loss of toluidine blue staining with focal areas of chondrocyte loss and/or collagen disruption in some affected joints, may have 1 or 2 digit joints with near total to total loss of cartilage.)

3 – Moderate (Generally moderate loss of toluidine blue staining with multifocal chondrocyte loss and/or collagen disruption in affected joints, may have 3 or 4 joints with near total or total loss. In the knee, some matrix remains on any affected surface with areas of severe matrix loss.)

4 – Marked (Marked loss of toluidine blue staining with multifocal marked (depth to deep zone or tidemark) chondrocyte loss and/or collagen disruption in most joints with a few unaffected or mildly affected. In the knee, one surface with total to near total cartilage loss.)

5 – Severe (Severe diffuse loss of toluidine blue staining with severe (depth to tide mark) chondrocyte loss and/or collagen disruption in most or all joints. In the knee, two or more surfaces with total to near total cartilage loss.)

*Bone resorption scores*

0 – Normal.

0.5 – Very minimal (Affects only 1 joint or is restricted to cortical/subperiosteal areas.)

1 – Minimal (Small/few areas of definite resorption, not readily apparent on low magnification, rare osteoclasts in affected joints, restricted to marginal zones.)

2 – Mild (More numerous/larger areas of resorption, osteoclasts more numerous in affected joints, mainly in marginal zones but some extension to load bearing areas, may have endosteal proliferation in areas of resorption.)

3 – Moderate (Obvious resorption of medullary trabecular and cortical bone without widespread full thickness defects in cortex, loss of medullary trabeculae, lesion apparent on low magnification, osteoclasts more numerous in affected joints, may have endosteal proliferation in areas of resorption.)

4 – Marked (Full thickness defects in cortical bone, often with distortion of profile of remaining cortical surface, marked loss of medullary bone, numerous osteoclasts, affects most joints, may have endosteal proliferation in areas of resorption.)

5 – Severe (Full thickness defects in cortical bone and destruction of joint architecture of all joints, may have endosteal proliferation in areas of resorption.)

*Periosteal new bone formation scores*

0 – Normal, no periosteal proliferation.

0.5 – Minimal focal or multifocal early proliferation, measures less than 40 !m width (< 1 unit on 25x).

1 – Minimal multifocal early proliferation, measures 40–119 µm width (1–2 units on 25x).

2 – Mild multifocal to diffuse with widths that measure 120–239 µm (3–5 units on 25x).

3 – Moderate diffuse with widths that measure 240–319 µm (6–7 units on 25x).

4 – Marked diffuse with widths that measure 320–439 µm (8–10 units on 25x).

5 – Severe, diffuse with widths that measure greater than 439 µm (> 10 units on 25x).

Scoring of joints in rat semi-established collagen-induced arthritis model

Collagen arthritic ankles and knees are given scores of 0–5 for inflammation, pannus formation

and bone resorption according to the following criteria:

*Knee and/or Ankle Inflammation*

0 – Normal.

0.5 – Minimal focal inflammation.

1 – Minimal infiltration of inflammatory cells in synovium/periarticular tissue.

2 – Mild infiltration.

3 – Moderate infiltration with moderate edema.

4 – Marked infiltration with marked edema.

5 – Severe infiltration with severe edema.

*Ankle pannus formation scores*

0 – Normal.

0.5 – Minimal infiltration of pannus in cartilage and subchondral bone, affects only marginal zones and affects only a few joints.

1 – Minimal infiltration of pannus in cartilage and subchondral bone, primarily affects marginal zones.

2 – Mild infiltration (<1/4 of tibia or tarsals at marginal zones).

3 – Moderate infiltration (1/4 to 1/3 of tibia or small tarsals affected at marginal zones).

4 – Marked infiltration (1/2 to 3/4 of tibia or tarsals affected at marginal zones).

5 – Severe infiltration (>3/4 of tibia or tarsals affected at marginal zones, severe distortion of overall architecture).

*Knee pannus formation scores*

0 – Normal.

0.5 – Minimal infiltration of pannus in cartilage and subchondral bone, affects only marginal zones and affects only a few joints.

1 – Minimal infiltration of pannus in cartilage and subchondral bone, approximately 1% – 10% of cartilage surface or subchondral bone affected.

2 – Mild infiltration (extends over up to 1/4 of surface or subchondral area of tibia or femur), approximately 11% – 25% of cartilage surface or subchondral bone affected

3 – Moderate infiltration (extends over > 1/4 but < 1/2 of surface or subchondral area of tibia or femur) approximately 26% – 50% of cartilage surface or subchondral bone affected.

4 – Marked infiltration (extends over 1/2 to 3/4 of tibial or femoral surface) approximately 51% – 75% of cartilage surface or subchondral bone affected.

5 – Severe infiltration approximately 76%–100% of cartilage surface or subchondral bone affected.

*Ankle cartilage damage scores (emphasis on small tarsals)*

0 – Normal.

0.5 – Very minimal (Minimal decrease in T blue staining, affects only marginal zones and affects only a few joints.)

1 – Minimal (Minimal to mild loss of toluidine blue staining with no obvious chondrocyte loss or collagen disruption.)

2 – Mild (Mild loss of toluidine blue staining with focal mild (superficial) chondrocyte loss and/or collagen disruption.)

3 – Moderate (Moderate loss of toluidine blue staining with multifocal moderate (depth to middle zone) chondrocyte loss and/or collagen disruption, smaller tarsals affected to 1/2 to 3/4 depth with rare areas of full thickness loss.)

4 – Marked (Marked loss of toluidine blue staining with multifocal marked (depth to deep zone) chondrocyte loss and/or collagen disruption, 1 or 2 small tarsals surfaces have full thickness loss of cartilage.)

5 – Severe (Severe diffuse loss of toluidine blue staining with multifocal severe (depth to tide mark) chondrocyte loss and/or collagen disruption affecting more than 2 cartilage surfaces.)

*Knee cartilage damage scores*

0 – Normal.

0.5 – Very minimal (Minimal decrease in T blue staining, affects only marginal zones.)

1 – Minimal (Minimal to mild loss of toluidine blue staining with no obvious chondrocyte loss or collagen disruption.)

2 – Mild (Mild loss of toluidine blue staining with focal mild (superficial) chondrocyte loss and/or collagen disruption, may have few small areas of 50% depth of cartilage affected.)

3 – Moderate (Moderate loss of toluidine blue staining with multifocal to diffuse moderate (depth to middle zone) chondrocyte loss and/or collagen disruption, may have 1–2 small areas of full thickness loss affecting less than ! of the total width of a surface and not more than 25% of the total width of all surfaces.)

4 – Marked (Marked loss of toluidine blue staining with multifocal to diffuse marked (depth to deep zone) chondrocyte loss and/or collagen disruption or 1 surface with near total loss and partial loss on others, total overall loss less than 50% of width of all surfaces combined.)

5 – Severe (Severe diffuse loss of toluidine blue staining with multifocal severe (depth to tide mark) chondrocyte loss and/or collagen disruption on both femurs and/or tibias, total overall loss greater than 50% of width of all surfaces combined.)

*Ankle bone resorption scores*

0 – Normal.

0.5 – Very minimal (Minimal resorption affects only marginal zones and affects only a few joints.)

1 – Minimal (Small areas of resorption, not readily apparent on low magnification, rare osteoclasts.)

2 – Mild (More numerous areas of resorption, not readily apparent on low magnification, osteoclasts more numerous, < 1/4 of tibia or tarsals at marginal zones resorbed.)

3 – Moderate (Obvious resorption of medullary trabecular and cortical bone without full thickness defects in cortex, loss of some medullary trabeculae, lesion apparent on low magnification, osteoclasts more numerous, 1/4 to 1/3 of tibia or tarsals affected at marginal zones.)

4 – Marked (Full thickness defects in cortical bone, often with distortion of profile of remaining cortical surface, marked loss of medullary bone, numerous osteoclasts, 1/2 to 3/4 of tibia or tarsals affected at marginal zones.)

5 – Severe (Full thickness defects in cortical bone, often with distortion of profile of remaining cortical surface, marked loss of medullary bone, numerous osteoclasts, > 3/4 of tibia or tarsals affected at marginal zones, severe distortion of overall architecture.)

*Knee bone resorption scores*

0 – Normal.

0.5 – Very minimal (Minimal resorption affects only marginal zones.)

1 – Minimal (Small areas of resorption, not readily apparent on low magnification, approximately 1%–10% of total joint width of subchondral bone affected.)

2 – Mild (More numerous areas of resorption, definite loss of subchondral bone, approximately 11% – 25% of total joint width of subchondral bone affected.)

3 – Moderate (Obvious resorption of subchondral bone approximately 26% – 50% of total joint width of subchondral bone affected.)

4 – Marked (Obvious resorption of subchondral bone approximately 51% – 75% of total joint width of subchondral bone affected.)

5 – Severe (Distortion of entire joint due to destruction approximately 76% – 100% of total joint width of subchondral bone affected.)

*Periarticular matrix deposition scores (only scored if an increase is seen in any treated group relative to disease controls)*

0 – Normal.

1 – Faint, multi-focal metachromatic staining, no excessive expansion of periarticular tissue.

2 – Darker, diffuse metachromatic staining, no excessive expansion of periarticular tissue.

3 – Darker, diffuse metachromatic staining, mild expansion of periarticular tissue.

4 – Darker, diffuse metachromatic staining, moderate expansion of periarticular tissue.

5 – Darker, diffuse metachromatic staining, severe expansion of periarticular tissue.

*Periosteal new bone formation scores (ankles-measure on 16X)*

0 – Normal, no periosteal proliferation.

0.5 – Minimal focal or multifocal proliferation, measures less than 127 μm width (1–2) at any location.

1 – Minimal multifocal proliferation, width at any location measures 127–252 μm (3–4 units).

2 – Mild multifocal on tarsals, diffuse in some locations, width at any location 253–441 μm (5–7 units).

3 – Moderate multifocal on tarsals, diffuse in most other locations, width at any location measures 442–630 μm (8–10 units).

4 – Marked multifocal on tarsals, diffuse at most other locations, width at any location measures 630–819 μm (11–13 units).

5 – Severe, multifocal on tarsals, diffuse at most other locations, width at any location measures > 819 μm (> 13 units).

# **Figures**

## **Figure S1.** Dose response curves of inhibitory activities of MT-SYK-03 against a. Syk (luminescent assay) and b. c-Src kinases.

|  |
| --- |
|  |

## **Figure S2.** Dependence of the number of activated B-cells on the inhibitor concentration during B-cell activation in human whole blood.

**Figure S3.** Inhibition of cytokine production by differentiated monocytes by MT-SYK-03 (or R406 as a reference).

**Figure S4.** Individual plasma concentration-time profiles of MT-SYK-03 after PO dose of a) 30 mg/kg, b) 100 mg/kg and c) 200 mg/kg in male Wistar rats (N = 6 for each concentration).

| *a.* 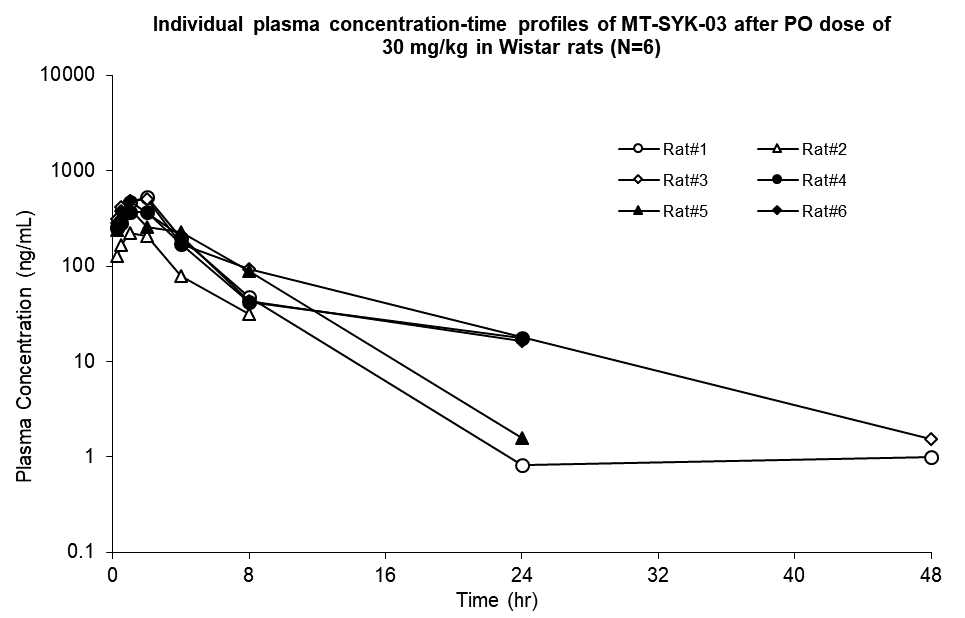 |
| --- |
| *b*.  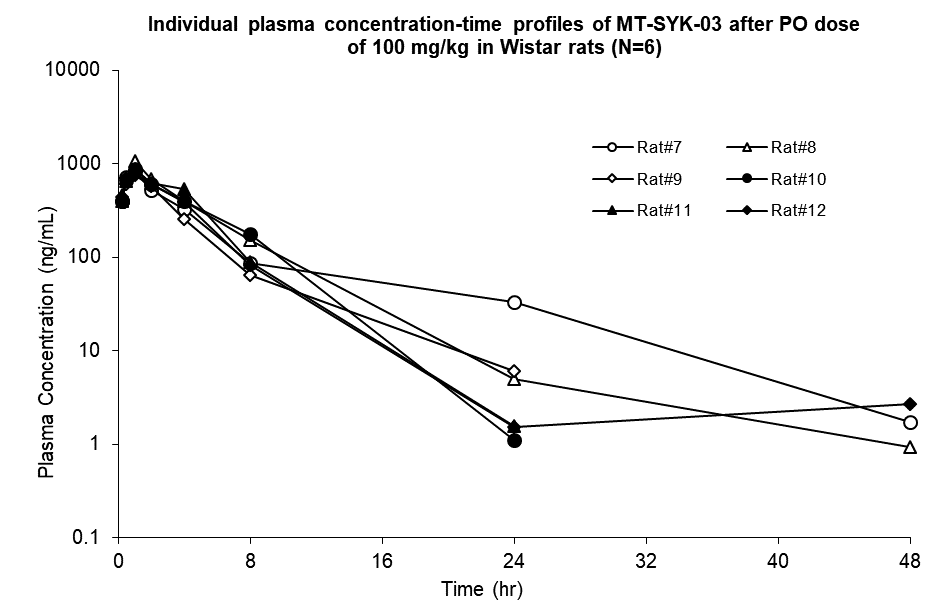 |
| *c*. 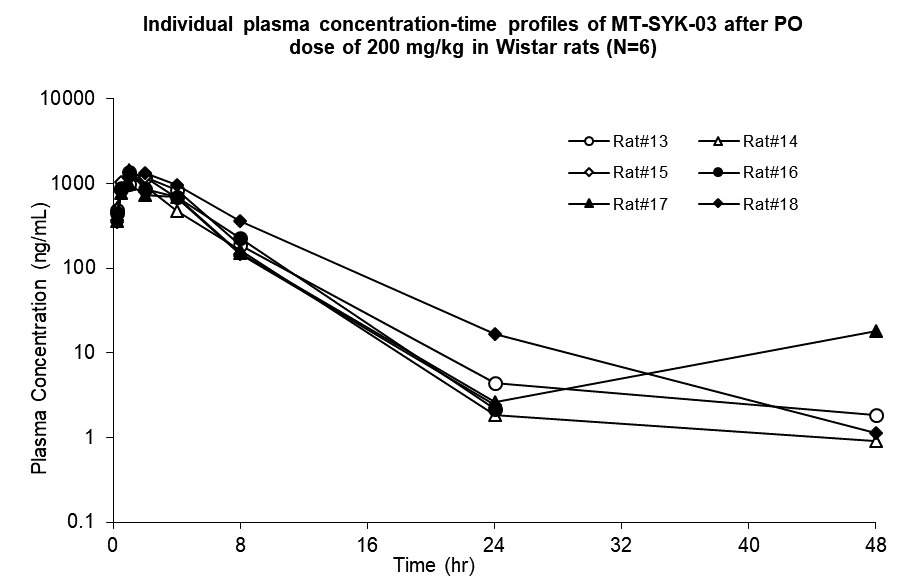 |

**Figure S6.** C_max_-dose dependence for MY-SYK-03 after single peroral administration in rats.

**Figure S7.** AUC-dose dependence for MY-SYK-03 after single peroral administration in rats.

**Figure S9**. Potential effects of MT-SYK-03 (PO, BID) alone or in combination with methotrexate in 16 days semi-established type II collagen arthritis in rats on ankle diameter measurements.


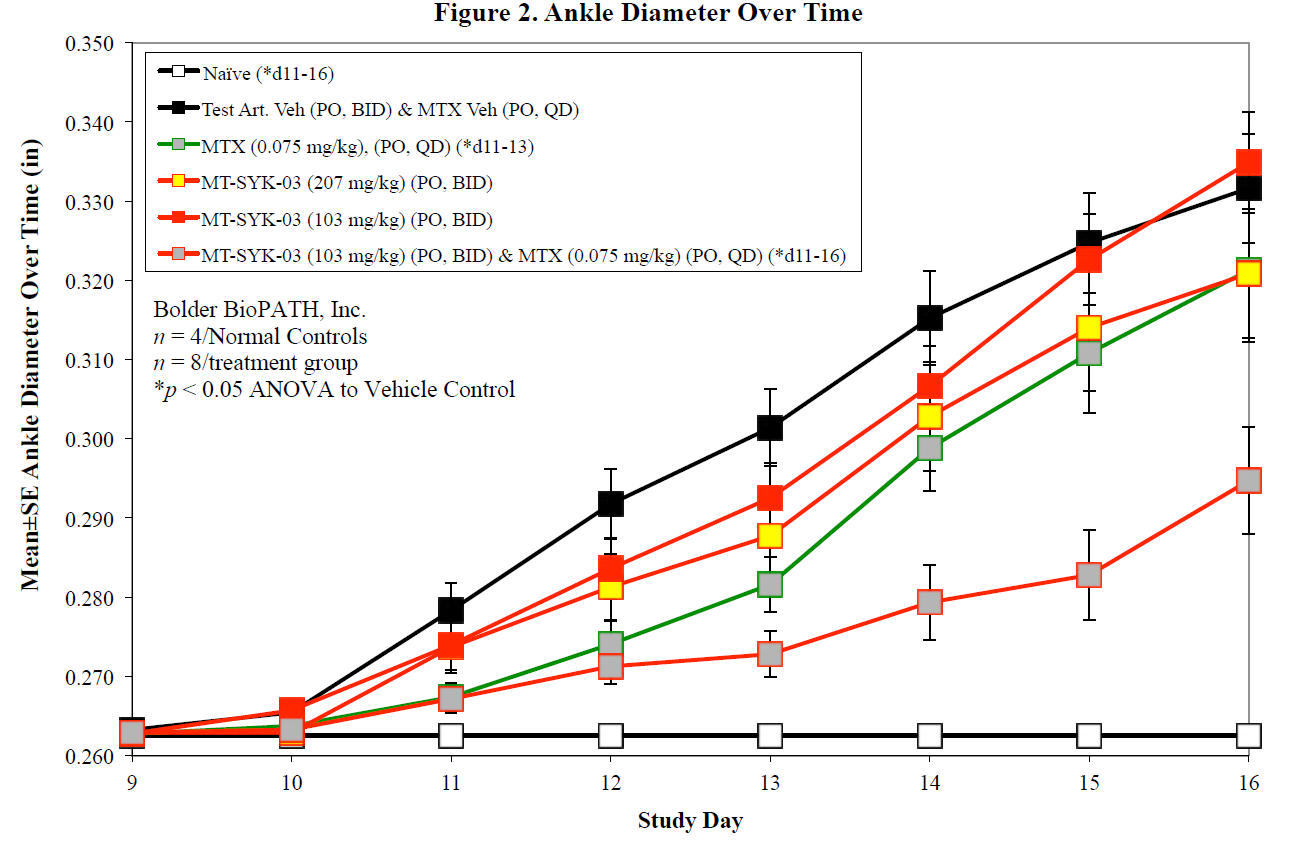


**Figure S10**. Potential effects of MT-SYK-03 (PO, BID) alone or in combination with methotrexate in 16 days semi-established type II collagen arthritis in rats on absolute paw weight.


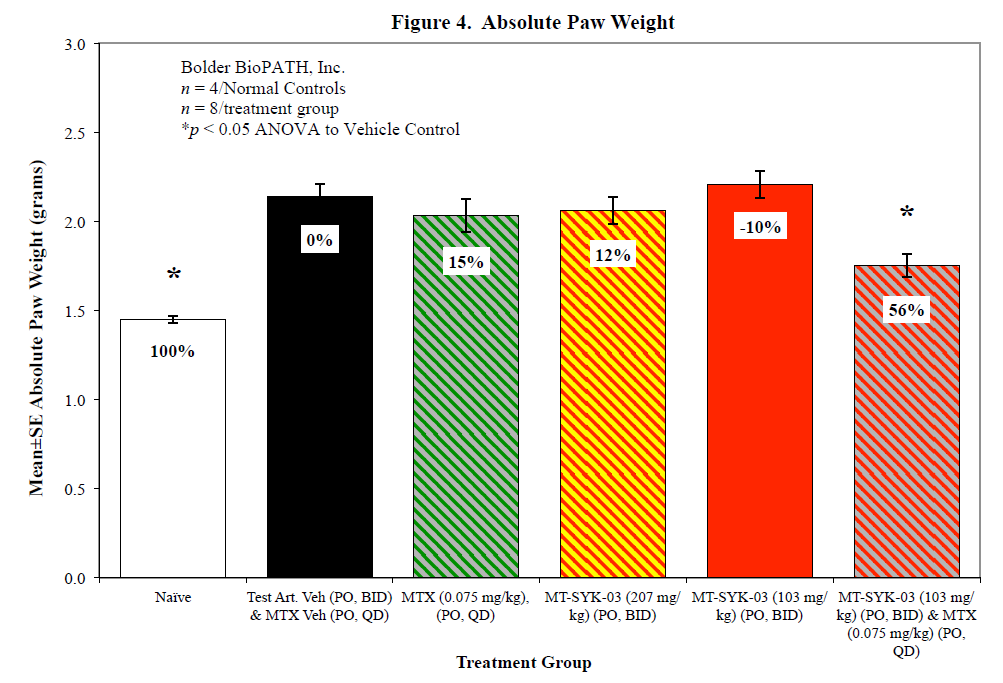


**Figure S11**. Potential effects of MT-SYK-03 (PO, BID) alone or in combination with methotrexate in 16 days semi-established type II collagen arthritis in rats on relative liver, spleen and thymus weight.


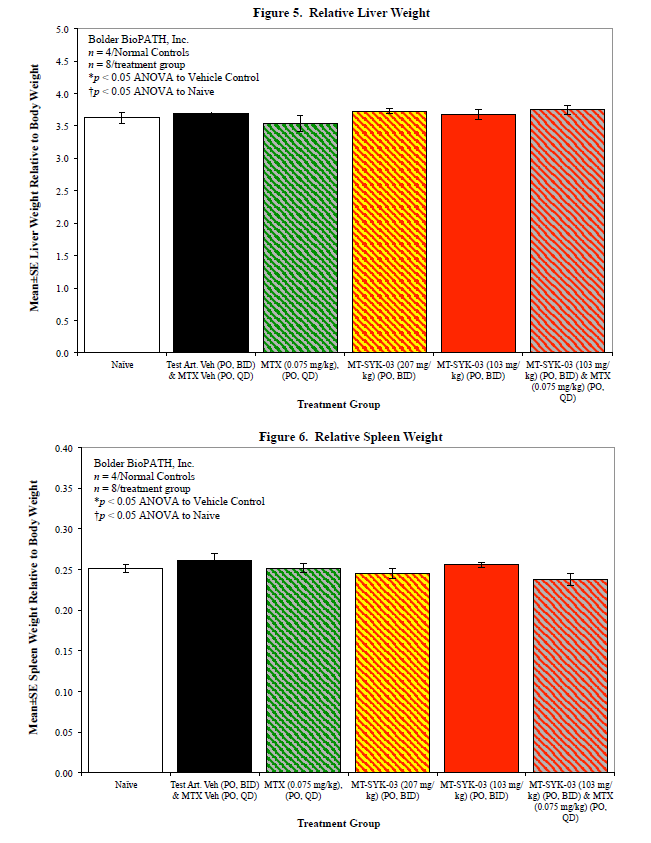


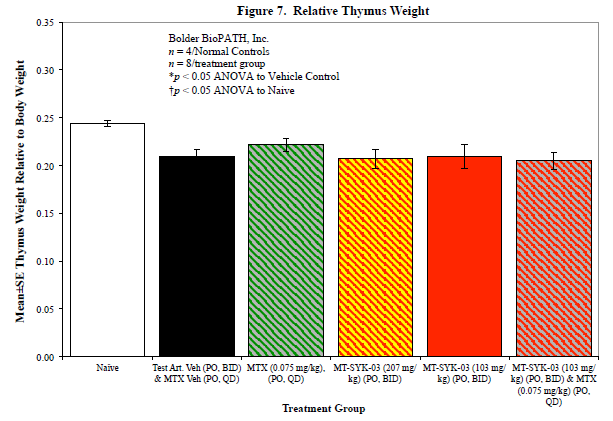


## **Figure S12.** Effect of MT-SYK-03 on chondrocyte hypertrophic-like changes induced *in vitro* by IL-1β. Aggrecan expression was evaluated in chondrocytes treated with or without MT-SYK-03 along with IL-1β. Expression was normalized against reference genes RPL19 and β-actin and compared to expression in untreated chondrocytes. Results are expressed as mean ± SD. * p < 0.05 compared to “IL-1β + DMSO 0.3%” using a pairwise fixed reallocation randomization test.


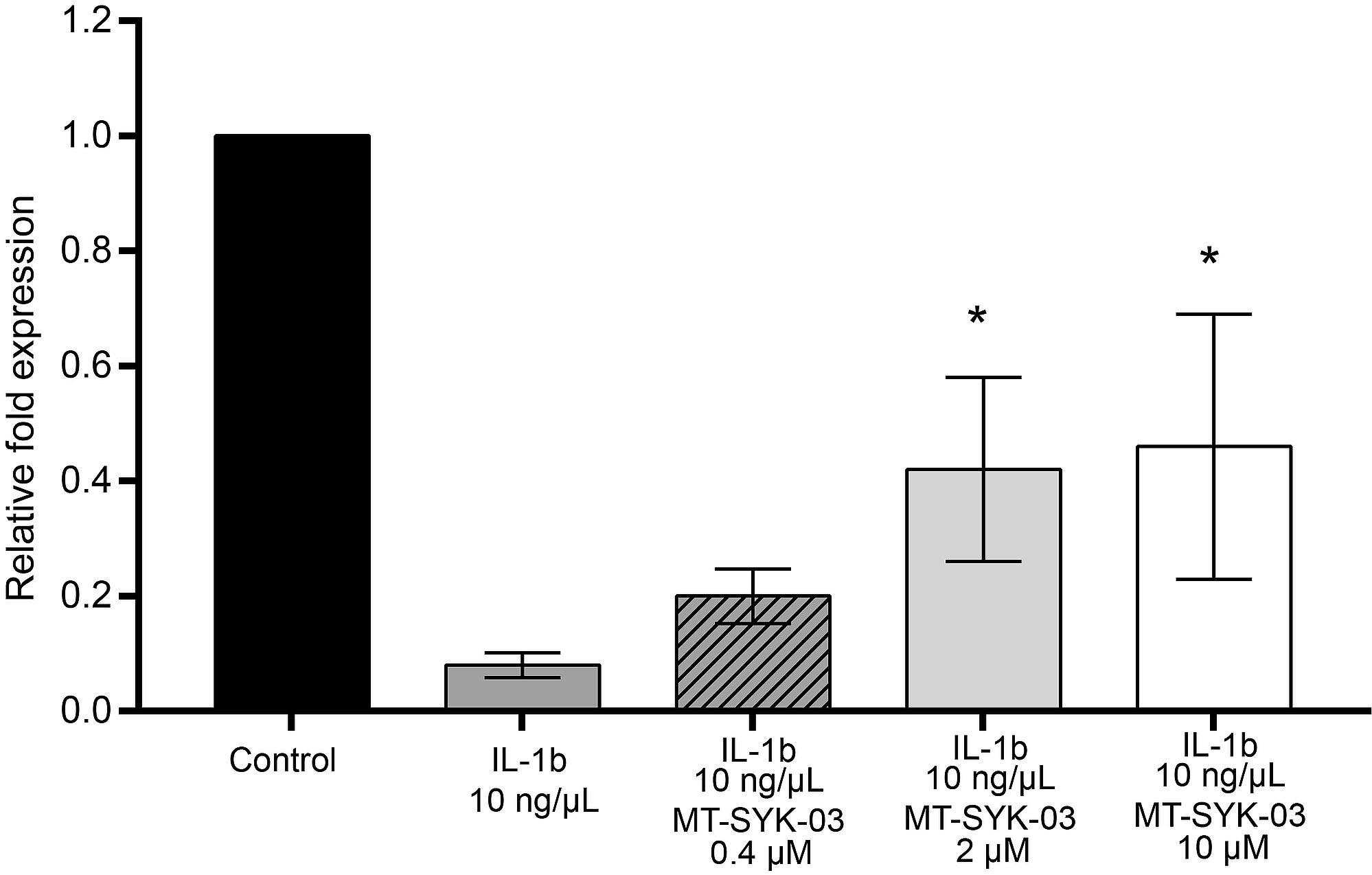


## **Figure S16.** Effects of oral administration of MT-SYK-03 (52 and 103 mg/kg BID) alone or in combination with MTX in DBA/1 11-day mice established type II collagen arthritis on inflammation, pannus formation, cartilage damage, bone resorption and periosteal bone formation in all joints. Individual histopathology parameters, Mean ± SE (n = 10 for vehicle and treatment groups and 4 for naive group, scored: 0=Normal, 1 = Minimal, 2 = Mild, 3 = Moderate, 4 = Marked, 5 = Severe). * p < 0.05 ANOVA to Vehicle Control, † p < 0.05 ANOVA to MTX (1.5 mg/kg), ‡ p ≤ 0.05 *t*-test to Vehicle Control. For raw data see Table S17.


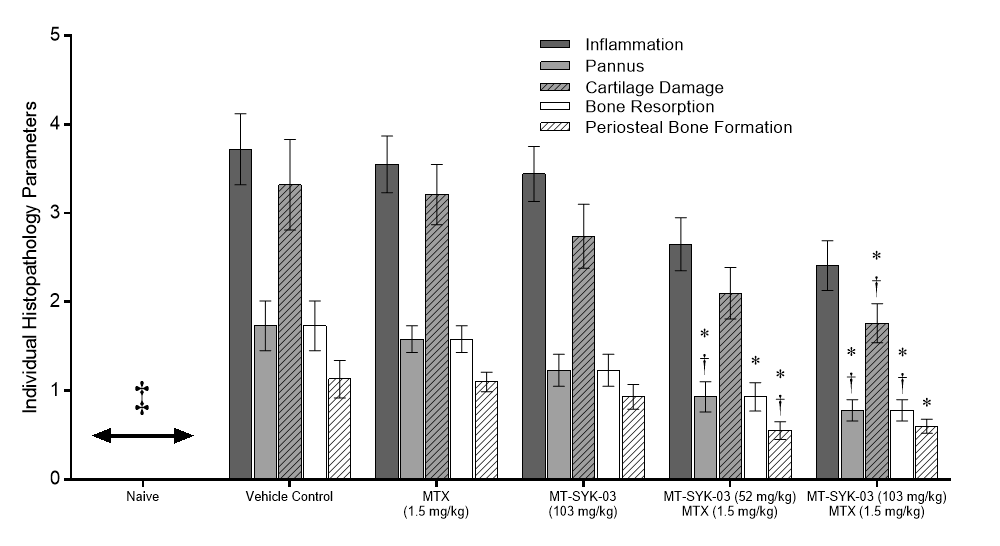


## **Figure S17**. Effects of oral administration of MT-SYK-03 (52 and 103 mg/kg BID) alone or in combination with MTX in DBA/1 11-day mice established type II collagen arthritis on periosteal bone width in all joints, paws and knees. Data presented as Mean ± SE (n = 10 for vehicle and treatment groups and 4 for naive group). * p < 0.05 ANOVA to Vehicle Control, † p < 0.05 ANOVA to MTX (1.5 mg/kg), ‡ p ≤ 0.05 *t*-test to Vehicle Control. For raw data see Table S18.


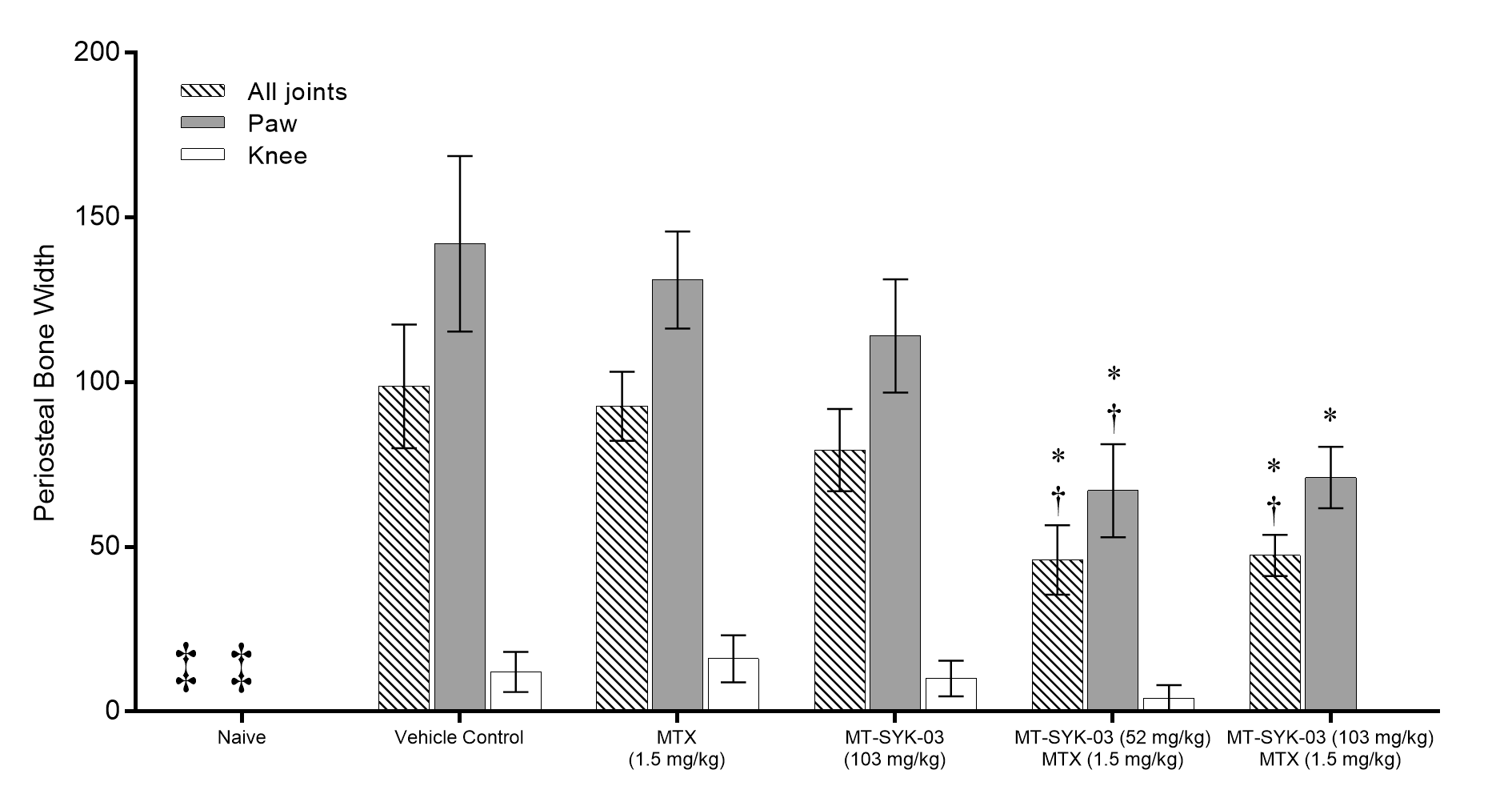


## **Figure S18.** MT-SYK-03 protects against cartilage damage in murine CIA. Photomicrographs of histologic sections of forepaw (16x) are shown for: a) naïve group; b) vehicle group; c) MT-SYK-03 (103 mg/kg); d) SYK-03 (103 mg/kg) + MTX (1.5 mg/kg). Arrows identify representative affected joints. W identifies wrist.

**
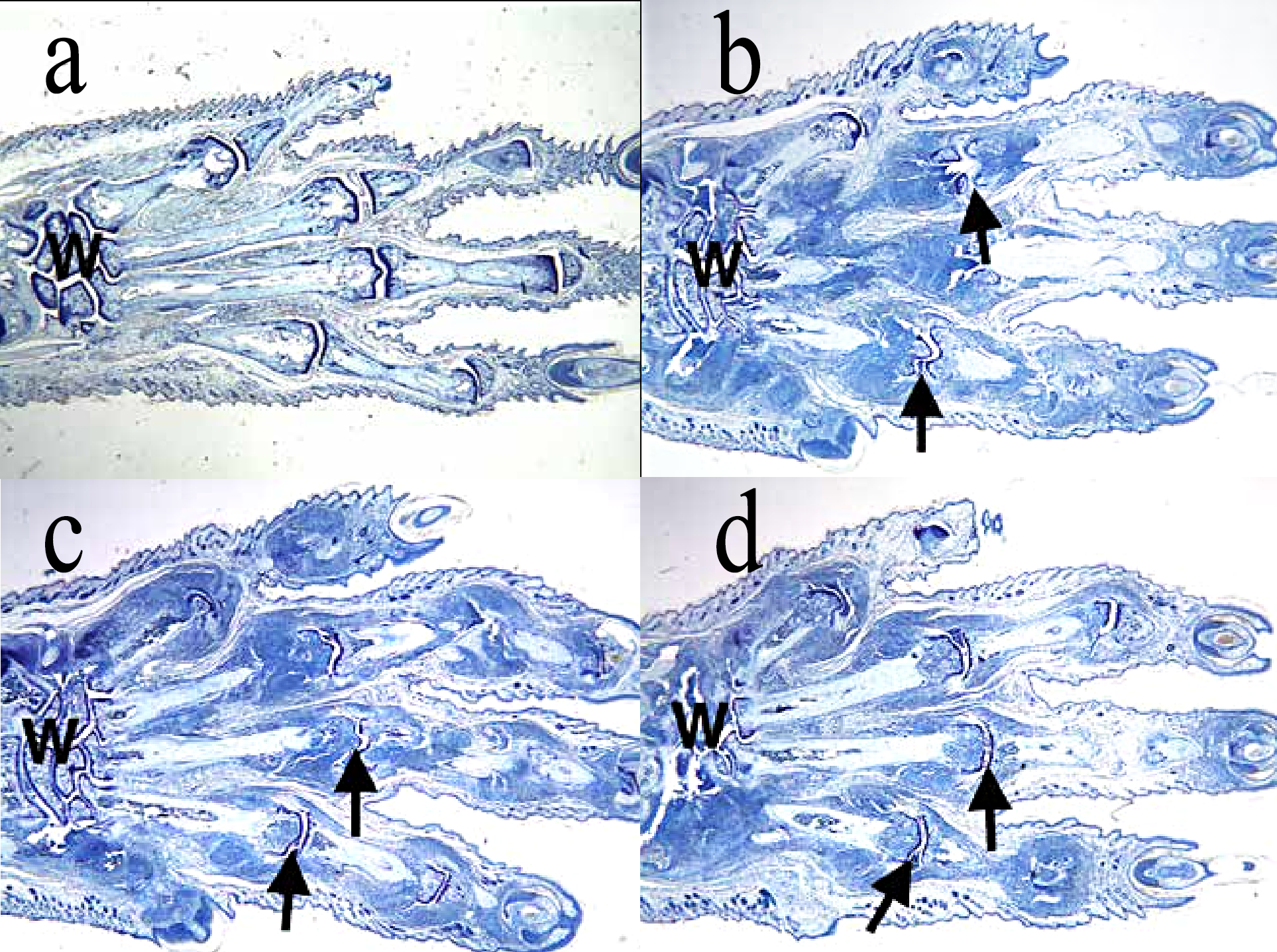
**

# **Figure S19.** Effect of the MT-SYK-03 and a reference compound (R406, right hand side of each pair) on phosphorylation of the main modulators of the B-cell cascade development (tyrosine kinases Syk, BTK, as well as CD19 receptor) and effector kinases of mitogen/stress-activated signal cascade (JNK1/2 and ERK1/2). Uncropped and unprocessed images from different blot fragments are presented. Each section corresponds to a different blot.

| Lanes in order of appearance:  Control (-, +); 10 uM (MT-SYK-03, R406), 3 uM (MT-SYK-03, R406), 1 uM (MT-SYK-03, R406) |  |
| --- | --- |
|  | Syk |
|  | p-Syk (Y525/526) |
|  | BTK |
|  | pBTK(Y223) |
|  | CD19 |
|  | pCD19 |
|  | JNK1/2 |
|  | pJNK1/2 |
|  | ERK1/2 |
|  | pERK1/2 |

# **Tables**

## **Table S1.** Calculated IC_50_ for Syk inhibition by MT-SYK-03 and R406 as a reference in luminescent and radiometric assays.

| **Compound** | **IC_50_, nM** | |
| --- | --- | --- |
|  | **luminescent assay** | **radiometric assay** |
| R406 | 42 ± 4 | 11 ± 2 |
| MT-SYK-03 | 23 ± 1 | 40 ± 4 |

## **Table S2.** Residual enzymatic activity of recombinant human kinases in the presence of inhibitors (MT-SYK-03, R406).

| **Kinase** | **Residual enzymatic activity, % (relative to DMSO)** | | | |
| --- | --- | --- | --- | --- |
|  | **R406 (500 nM)** | | **MT-SYK-03 (500 nM)** | |
|  | **%** | **[min %; max %]** | **%** | **[min %; max %]** |
| ABL1 | 68.6 | [67.4; 69.8] | 68.0 | [63.6; 72.5] |
| ABL2/ARG | 34.5 | [34.2; 34.9] | 32.3 | [32.1; 32.5] |
| ACK1 | 0.6 | [0.4; 0.9] | 5.4 | [5.3; 5.5] |
| AKT1 | 99.4 | [97.6; 101.2] | 100.9 | [98.4; 103.4] |
| AKT2 | 110.3 | [108.3; 112.3] | 106.1 | [101.1; 111.1] |
| AKT3 | 95.2 | [90.8; 99.7] | 102.6 | [101.7; 103.5] |
| ALK | 41.2 | [37; 45.3] | 96.5 | [91.2; 101.8] |
| ALK1/ACVRL1 | 100.5 | [100.2; 100.8] | 125.7 | [121.7; 129.8] |
| ALK2/ACVR1 | 134.7 | [110.5; 158.9] | 240.6 | [237.2; 244] |
| ALK3/BMPR1A | 119.9 | [119.88; 119.93] | 130.3 | [125.6; 135] |
| ALK4/ACVR1B | 133.2 | [131.8; 134.7] | 125.4 | [125.2; 125.7] |
| ALK5/TGFBR1 | 107.7 | [103.7; 111.7] | 104.5 | [104.3; 104.7] |
| ALK6/BMPR1B | 145.2 | [143.8; 146.7] | 143.1 | [142.4; 143.8] |
| ARAF | 41.0 | [39.9; 42.1] | 59.7 | [56.7; 62.7] |
| ARK5/NUAK1 | 8.7 | [8.2; 9.1] | 49.7 | [49.71; 49.73] |
| ASK1/MAP3K5 | 97.1 | [95.4; 98.9] | 102.1 | [100.2; 104.1] |
| Aurora A | 10.9 | [10.8; 11] | 74.4 | [73.8; 74.9] |
| Aurora B | 40.2 | [36.4; 44.1] | 78.6 | [78; 79.2] |
| Aurora C | 42.1 | [41; 43.2] | 89.2 | [88.6; 89.7] |
| AXL | 44.5 | [43.4; 45.6] | 74.3 | [73.5; 75] |
| BLK | 16.7 | [14.9; 18.6] | 19.8 | [19.5; 20.1] |
| BMPR2 | 71.1 | [68.4; 73.8] | 90.0 | [87.4; 92.6] |
| BMX/ETK | 7.6 | [7.3; 7.9] | 18.0 | [16.8; 19.2] |
| BRAF | 95.9 | [86.3; 105.5] | 86.7 | [80.4; 92.9] |
| BRK | 27.6 | [24.2; 30.9] | 54.2 | [52.5; 55.9] |
| BRSK1 | 61.6 | [58.2; 64.9] | 92.6 | [89.3; 96] |
| BRSK2 | 84.2 | [82.9; 85.4] | 92.5 | [90.3; 94.8] |
| BTK | 28.8 | [27.6; 30.1] | 51.3 | [48.2; 54.4] |
| c-Kit | 37.1 | [34.4; 39.9] | 69.2 | [64.9; 73.4] |
| c-MER | 73.9 | [71.2; 76.5] | 108.9 | [106.7; 111] |
| c-MET | 71.4 | [63.8; 79] | 108.1 | [107.2; 109] |
| c-Src | 6.8 | [5.8; 7.7] | 6.2 | [6.1; 6.3] |
| CAMK1a | 77.1 | [76.9; 77.4] | 100.2 | [100; 100.5] |
| CAMK1b | 108.0 | [107.2; 108.9] | 106.4 | [105.7; 107.1] |
| CAMK1d | 81.4 | [80.1; 82.7] | 108.4 | [106.7; 110.2] |
| CAMK1g | 84.9 | [78.9; 90.8] | 99.2 | [94.7; 103.7] |
| CAMK2a | 94.4 | [92.3; 96.5] | 94.1 | [92; 96.2] |
| CAMK2b | 95.8 | [95.5; 96.2] | 97.7 | [95.9; 99.4] |
| CAMK2d | 98.9 | [97.1; 100.8] | 104.3 | [102.1; 106.5] |
| CAMK2g | 101.4 | [98.7; 104.1] | 100.1 | [95.9; 104.4] |
| CAMK4 | 119.7 | [112.6; 126.9] | 111.1 | [104.8; 117.3] |
| CAMKK1 | 91.9 | [91.6; 92.3] | 102.4 | [99.7; 105.1] |
| CAMKK2 | 88.8 | [81.7; 96] | 97.2 | [89.8; 104.6] |
| CDC7/DBF4 | 82.6 | [81; 84.2] | 101.8 | [97.8; 105.8] |
| CDK1/cyclin A | 74.2 | [67.1; 81.3] | 110.5 | [105; 116.1] |
| CDK1/cyclin B | 75.6 | [72.4; 78.7] | 96.0 | [92.5; 99.5] |
| CDK1/cyclin E | 76.5 | [73; 79.9] | 94.6 | [93.6; 95.5] |
| CDK16/cyclin Y | 63.3 | [61.9; 64.7] | 95.8 | [94.4; 97.2] |
| CDK2/cyclin A | 47.6 | [47.2; 47.9] | 83.1 | [79; 87.1] |
| CDK2/Cyclin A1 | 59.5 | [55.5; 63.4] | 90.7 | [86.6; 94.8] |
| CDK2/cyclin E | 60.0 | [56.4; 63.5] | 82.1 | [78.2; 86.1] |
| CDK3/cyclin E | 90.3 | [83.4; 97.1] | 99.0 | [98.5; 99.5] |
| CDK4/cyclin D1 | 104.5 | [100.5; 108.6] | 108.7 | [107.5; 109.9] |
| CDK4/cyclin D3 | 99.6 | [98.2; 100.9] | 100.0 | [98.4; 101.5] |
| CDK5/p25 | 79.4 | [78.7; 80.1] | 101.7 | [95.6; 107.8] |
| CDK5/p35 | 82.9 | [81.5; 84.4] | 101.2 | [99.8; 102.5] |
| CDK6/cyclin D1 | 93.4 | [91.9; 95] | 91.8 | [91.3; 92.3] |
| CDK6/cyclin D3 | 97.4 | [97.36; 97.41] | 101.0 | [100.1; 101.8] |
| CDK7/cyclin H | 81.7 | [80.2; 83.2] | 99.6 | [98.1; 101.1] |
| CDK9/cyclin K | 84.9 | [84.85; 84.92] | 95.7 | [94.7; 96.7] |
| CDK9/cyclin T1 | 99.5 | [93.9; 105.1] | 118.4 | [106.6; 130.3] |
| CHK1 | 78.6 | [78.2; 79] | 91.4 | [86; 96.9] |
| CHK2 | 50.7 | [49.6; 51.8] | 95.0 | [93.4; 96.5] |
| CK1a1 | 95.5 | [94.1; 96.8] | 100.8 | [98.4; 103.3] |
| CK1d | 78.7 | [73.6; 83.7] | 86.5 | [83; 89.9] |
| CK1epsilon | 103.6 | [101.1; 106.1] | 98.0 | [95.9; 100.1] |
| CK1g1 | 92.7 | [91.6; 93.8] | 93.5 | [91.5; 95.5] |
| CK1g2 | 101.3 | [96; 106.6] | 97.2 | [87.6; 106.8] |
| CK1g3 | 79.6 | [79; 80.3] | 72.2 | [69.5; 75] |
| CK2a | 50.8 | [45.5; 56.1] | 94.3 | [91.8; 96.7] |
| CK2a2 | 21.4 | [20.5; 22.2] | 66.0 | [62.8; 69.2] |
| CLK1 | 61.7 | [59.9; 63.5] | 90.4 | [87.8; 93] |
| CLK2 | 81.6 | [80.8; 82.4] | 94.5 | [90.6; 98.4] |
| CLK3 | 89.4 | [88.2; 90.6] | 102.9 | [101.2; 104.5] |
| CLK4 | 52.1 | [49.9; 54.3] | 88.7 | [79.4; 98] |
| COT1/MAP3K8 | 100.4 | [97.4; 103.4] | 90.4 | [89.5; 91.3] |
| CSK | 20.6 | [15; 26.1] | 34.8 | [34.5; 35.1] |
| CTK/MATK | 119.7 | [117.7; 121.7] | 96.6 | [95.6; 97.6] |
| DAPK1 | 65.2 | [64.1; 66.3] | 112.6 | [104.7; 120.6] |
| DAPK2 | 102.6 | [101; 104.3] | 98.4 | [90.5; 106.3] |
| DCAMKL1 | 84.2 | [81.7; 86.7] | 97.6 | [94.3; 100.9] |
| DCAMKL2 | 91.8 | [87.3; 96.2] | 99.1 | [96.7; 101.5] |
| DDR1 | 1.0 | [0.96; 1.02] | 4.7 | [4.1; 5.3] |
| DDR2 | 31.5 | [29.4; 33.7] | 69.0 | [63.7; 74.4] |
| DLK/MAP3K12 | 70.6 | [67.6; 73.7] | 83.7 | [80.3; 87] |
| DMPK | 90.5 | [89.5; 91.6] | 94.3 | [92.9; 95.7] |
| DMPK2 | 57.5 | [56.9; 58.1] | 96.4 | [94.5; 98.2] |
| DRAK1/STK17A | 43.3 | [41.1; 45.5] | 93.6 | [90; 97.2] |
| DYRK1/DYRK1A | 53.0 | [49.9; 56.1] | 90.5 | [90.1; 90.9] |
| DYRK1B | 72.5 | [68.4; 76.5] | 90.6 | [88.7; 92.5] |
| DYRK2 | 64.1 | [58; 70.1] | 104.5 | [101.8; 107.1] |
| DYRK3 | 90.6 | [86.7; 94.6] | 91.6 | [89.7; 93.5] |
| DYRK4 | 77.1 | [74.5; 79.7] | 104.3 | [102.3; 106.3] |
| EGFR | 54.2 | [48.8; 59.7] | 59.6 | [55.2; 64] |
| EPHA1 | 25.4 | [25.2; 25.6] | 48.3 | [46.6; 50.1] |
| EPHA2 | 30.9 | [30.2; 31.7] | 56.3 | [54; 58.5] |
| EPHA3 | 85.6 | [83.3; 88] | 91.8 | [89.3; 94.3] |
| EPHA4 | 42.8 | [39.4; 46.2] | 58.1 | [55.9; 60.4] |
| EPHA5 | 60.9 | [60; 61.8] | 76.4 | [76.3; 76.6] |
| EPHA6 | 24.9 | [23.6; 26.2] | 73.9 | [71.5; 76.4] |
| EPHA7 | 10.5 | [9.9; 11.1] | 58.4 | [56.5; 60.3] |
| EPHA8 | 92.6 | [90.7; 94.6] | 99.7 | [97; 102.5] |
| EPHB1 | -4.0 | [-7.5; -0.5] | 9.6 | [6.8; 12.5] |
| EPHB2 | 69.5 | [65; 74.1] | 77.7 | [74; 81.3] |
| EPHB3 | 84.4 | [82.4; 86.4] | 94.2 | [87.1; 101.2] |
| EPHB4 | 51.2 | [49.1; 53.4] | 64.6 | [62.1; 67.1] |
| ERBB2/HER2 | 80.5 | [79.8; 81.3] | 82.2 | [81.5; 82.8] |
| ERBB4/HER4 | 68.2 | [66.3; 70.1] | 79.4 | [76.6; 82.1] |
| ERK1 | 97.4 | [88.5; 106.3] | 119.4 | [109.2; 129.7] |
| ERK2/MAPK1 | 88.0 | [86.4; 89.6] | 90.6 | [90; 91.1] |
| ERK5/MAPK7 | 88.2 | [85.3; 91.1] | 92.2 | [85.3; 99.1] |
| ERK7/MAPK15 | 77.1 | [73.3; 80.9] | 100.4 | [98.6; 102.1] |
| FAK/PTK2 | 29.7 | [28.6; 30.7] | 87.4 | [86.3; 88.6] |
| FER | 22.9 | [19.8; 26.1] | 78.8 | [74.7; 83] |
| FES/FPS | 46.2 | [43.6; 48.9] | 92.2 | [89.5; 94.8] |
| FGFR1 | 29.0 | [25.8; 32.1] | 77.5 | [74.5; 80.6] |
| FGFR2 | 14.9 | [13.5; 16.4] | 65.1 | [62; 68.2] |
| FGFR3 | 17.4 | [13.7; 21.1] | 57.2 | [55.8; 58.5] |
| FGFR4 | 58.0 | [55.3; 60.6] | 95.7 | [86; 105.3] |
| FGR | 15.7 | [14.7; 16.7] | 22.6 | [21.2; 23.9] |
| FLT1/VEGFR1 | 12.4 | [10.4; 14.4] | 65.4 | [63.8; 67.1] |
| FLT3 | 6.2 | [4; 8.3] | 5.7 | [5.2; 6.3] |
| FLT4/VEGFR3 | 4.9 | [4.85; 4.86] | 40.4 | [39.9; 40.9] |
| FMS | 17.2 | [16.1; 18.4] | 51.1 | [50.7; 51.6] |
| FRK/PTK5 | 35.0 | [34.9; 35.2] | 56.5 | [52.6; 60.3] |
| FYN | 18.8 | [17.4; 20.1] | 25.2 | [21.3; 29.1] |
| GCK/MAP4K2 | 77.5 | [77.4; 77.6] | 100.4 | [97.6; 103.1] |
| GLK/MAP4K3 | 65.8 | [65.1; 66.4] | 94.1 | [88.9; 99.4] |
| GRK1 | 96.0 | [95.3; 96.8] | 105.1 | [100.1; 110.2] |
| GRK2 | 97.2 | [96.3; 98.2] | 100.9 | [97.8; 104] |
| GRK3 | 102.5 | [101.7; 103.2] | 103.3 | [102.2; 104.3] |
| GRK4 | 96.8 | [95.7; 97.9] | 99.7 | [97.7; 101.7] |
| GRK5 | 96.4 | [96.41; 96.47] | 103.3 | [103.2; 103.5] |
| GRK6 | 92.7 | [86.5; 98.9] | 100.1 | [91.8; 108.5] |
| GRK7 | 89.6 | [88; 91.2] | 99.7 | [99; 100.5] |
| GSK3a | 27.5 | [24.1; 30.9] | 74.2 | [70.5; 77.8] |
| GSK3b | 37.9 | [37.8; 37.9] | 82.4 | [79.1; 85.7] |
| Haspin | 91.5 | [90.8; 92.1] | 100.8 | [97.6; 104.1] |
| HCK | 15.4 | [14; 16.8] | 26.3 | [26; 26.5] |
| HGK/MAP4K4 | 75.3 | [74.3; 76.4] | 78.4 | [74.7; 82.1] |
| HIPK1 | 90.8 | [86.5; 95.2] | 97.4 | [95.1; 99.8] |
| HIPK2 | 94.9 | [89.3; 100.6] | 103.3 | [102.6; 104.1] |
| HIPK3 | 96.4 | [93.9; 99] | 110.0 | [108.2; 111.8] |
| HIPK4 | 92.9 | [91.9; 93.8] | 112.3 | [103; 121.6] |
| HPK1/MAP4K1 | 98.8 | [97.8; 99.8] | 107.2 | [107.1; 107.4] |
| IGF1R | 60.3 | [57.2; 63.3] | 90.0 | [87.7; 92.2] |
| IKKa/CHUK | 89.9 | [87.9; 91.9] | 100.2 | [99.1; 101.3] |
| IKKb/IKBKB | 53.0 | [49.1; 56.8] | 94.0 | [88.9; 99.2] |
| IKKe/IKBKE | 16.1 | [15.3; 16.9] | 77.5 | [75.9; 79.1] |
| IR | 77.5 | [72; 82.9] | 94.4 | [91.6; 97.1] |
| IRAK1 | 13.5 | [12.5; 14.4] | 74.6 | [74.62; 74.67] |
| IRAK4 | 41.9 | [37.9; 45.9] | 103.4 | [95.8; 110.9] |
| IRR/INSRR | 73.6 | [70.9; 76.4] | 89.0 | [86.7; 91.4] |
| ITK | 54.5 | [54.53; 54.56] | 101.9 | [99.3; 104.5] |
| JAK1 | 0.0 | [-3; 3] | 63.4 | [59.5; 67.3] |
| JAK2 | 0.9 | [0.5; 1.2] | 27.0 | [25.2; 28.8] |
| JAK3 | 10.4 | [9.2; 11.5] | 74.7 | [71.3; 78] |
| JNK1 | 38.1 | [36.4; 39.8] | 94.3 | [94.1; 94.6] |
| JNK2 | 52.5 | [49.6; 55.4] | 92.6 | [90.3; 94.9] |
| JNK3 | 114.0 | [113.3; 114.7] | 96.4 | [96; 96.8] |
| KDR/VEGFR2 | 25.1 | [25; 25.2] | 88.6 | [86.4; 90.8] |
| KHS/MAP4K5 | 87.1 | [85.4; 88.8] | 91.8 | [87.3; 96.4] |
| LATS1 | 79.6 | [74.1; 85] | 99.4 | [95.4; 103.5] |
| LATS2 | 51.2 | [49.6; 52.9] | 85.3 | [82.8; 87.9] |
| LCK | 6.1 | [5.1; 7.1] | 12.0 | [12.01; 12.07] |
| LCK2/ICK | 99.8 | [97.5; 102] | 109.6 | [107.6; 111.6] |
| LIMK1 | 7.2 | [6.7; 7.7] | 11.4 | [10.5; 12.3] |
| LIMK2 | 77.5 | [74.3; 80.7] | 84.9 | [83.8; 86] |
| LKB1 | 69.3 | [67.8; 70.8] | 101.1 | [99.8; 102.3] |
| LOK/STK10 | 37.4 | [36.7; 38] | 75.7 | [75.6; 75.8] |
| LRRK2 | 5.5 | [4.9; 6.1] | 48.0 | [44.8; 51.3] |
| LYN | 5.7 | [5.3; 6] | 12.5 | [11.2; 13.8] |
| LYN B | 14.5 | [13.5; 15.5] | 25.7 | [25.2; 26.2] |
| MAPKAPK2 | 106.2 | [106.15; 106.2] | 101.4 | [100.6; 102.2] |
| MAPKAPK3 | 101.9 | [100.4; 103.3] | 102.0 | [97.9; 106.1] |
| MAPKAPK5/PRAK | 99.4 | [98.1; 100.8] | 92.3 | [87.4; 97.2] |
| MARK1 | 57.5 | [53.2; 61.7] | 95.7 | [93.8; 97.6] |
| MARK2/PAR-1Ba | 53.7 | [52.2; 55.3] | 97.9 | [95.9; 100] |
| MARK3 | 51.8 | [50.7; 53] | 97.1 | [94.9; 99.4] |
| MARK4 | 46.7 | [43.9; 49.5] | 92.1 | [82.9; 101.4] |
| MEK1 | 87.2 | [81.2; 93.3] | 106.8 | [101.3; 112.2] |
| MEK2 | 83.5 | [81.4; 85.5] | 97.2 | [94.4; 100.1] |
| MEK3 | 131.3 | [131; 131.6] | 119.2 | [117.9; 120.4] |
| MEKK1 | 100.0 | [99.8; 100.2] | 104.8 | [103.9; 105.7] |
| MEKK2 | 56.8 | [54; 59.6] | 106.5 | [101.2; 111.8] |
| MEKK3 | 76.1 | [76; 76.2] | 116.5 | [112.9; 120] |
| MELK | 54.7 | [52.7; 56.7] | 86.6 | [84.9; 88.3] |
| MINK/MINK1 | 87.3 | [82.2; 92.3] | 92.0 | [86.3; 97.6] |
| MKK4 | 102.1 | [98.6; 105.6] | 99.8 | [96.7; 102.9] |
| MKK6 | 111.8 | [109; 114.7] | 116.2 | [113.8; 118.6] |
| MLCK/MYLK | 90.5 | [89.5; 91.5] | 96.2 | [92.7; 99.7] |
| MLCK2/MYLK2 | 89.2 | [79.7; 98.8] | 104.8 | [98.5; 111.1] |
| MLK1/MAP3K9 | 6.3 | [-2.6; 15.1] | 21.5 | [20.9; 22] |
| MLK2/MAP3K10 | 1.1 | [-1.9; 4.2] | 7.5 | [6; 8.9] |
| MLK3/MAP3K11 | 3.0 | [2.2; 3.9] | 31.4 | [29.9; 32.8] |
| MNK1 | 61.5 | [57.6; 65.3] | 91.2 | [86.7; 95.7] |
| MNK2 | 37.9 | [35.2; 40.5] | 78.6 | [77.4; 79.8] |
| MRCKa/CDC42BPA | 104.0 | [99.5; 108.4] | 102.2 | [101.4; 103] |
| MRCKb/CDC42BPB | 104.8 | [104.7; 104.9] | 103.0 | [100.5; 105.5] |
| MSK1/RPS6KA5 | 97.3 | [95.7; 98.9] | 106.1 | [106; 106.2] |
| MSK2/RPS6KA4 | 97.7 | [96.9; 98.6] | 97.9 | [93.1; 102.7] |
| MSSK1/STK23 | 107.3 | [106.5; 108] | 106.5 | [105.4; 107.6] |
| MST1/STK4 | 86.3 | [80.4; 92.1] | 94.2 | [92.1; 96.2] |
| MST2/STK3 | 97.9 | [97.6; 98.2] | 105.1 | [102.3; 107.9] |
| MST3/STK24 | 95.6 | [92.3; 98.9] | 107.9 | [107.1; 108.8] |
| MST4 | 94.8 | [93.5; 96] | 111.5 | [106.5; 116.6] |
| MUSK | 62.1 | [61.6; 62.7] | 79.9 | [79.8; 80] |
| MYLK3 | 96.4 | [95.6; 97.1] | 95.9 | [91.8; 100] |
| MYO3b | 94.1 | [88.8; 99.4] | 95.4 | [91.9; 99] |
| NEK1 | 7.0 | [6.7; 7.2] | 52.7 | [49.6; 55.7] |
| NEK11 | 77.3 | [77; 77.5] | 35.9 | [33.9; 37.9] |
| NEK2 | 54.5 | [50.8; 58.2] | 86.0 | [84.6; 87.4] |
| NEK3 | 19.2 | [17.4; 21] | 72.0 | [68.7; 75.2] |
| NEK4 | 52.1 | [49.2; 55] | 109.4 | [109.42; 109.45] |
| NEK5 | 27.1 | [25.8; 28.5] | 63.6 | [59.7; 67.6] |
| NEK6 | 70.5 | [67.4; 73.5] | 103.2 | [99.9; 106.4] |
| NEK7 | 62.3 | [61.9; 62.7] | 95.9 | [94.9; 96.9] |
| NEK9 | 2.5 | [2.3; 2.7] | 55.7 | [54.2; 57.2] |
| NLK | 107.0 | [101.3; 112.7] | 94.9 | [93.9; 95.8] |
| OSR1/OXSR1 | 97.2 | [94.8; 99.5] | 98.2 | [96; 100.5] |
| P38a/MAPK14 | 99.9 | [94.6; 105.2] | 109.8 | [108.3; 111.4] |
| P38b/MAPK11 | 101.3 | [100.3; 102.4] | 103.3 | [101.3; 105.2] |
| P38d/MAPK13 | 84.7 | [84.7; 84.71] | 119.2 | [109.8; 128.6] |
| P38g | 90.5 | [90.4; 90.6] | 101.3 | [99.9; 102.7] |
| p70S6K/RPS6KB1 | 49.1 | [48.7; 49.5] | 98.3 | [96.7; 99.8] |
| p70S6Kb/RPS6KB2 | 74.6 | [70.6; 78.7] | 91.4 | [87.4; 95.4] |
| PAK1 | 79.1 | [78.2; 80.1] | 100.0 | [97.7; 102.2] |
| PAK2 | 58.4 | [56.3; 60.4] | 94.1 | [94.04; 94.07] |
| PAK3 | 30.9 | [30.7; 31.1] | 87.0 | [85.3; 88.7] |
| PAK4 | 53.9 | [51; 56.7] | 92.1 | [89.5; 94.7] |
| PAK5 | 49.0 | [43.2; 54.8] | 98.5 | [97.3; 99.8] |
| PAK6 | 81.2 | [77; 85.3] | 96.5 | [92.8; 100.3] |
| PASK | 105.7 | [101.9; 109.5] | 102.3 | [99.1; 105.4] |
| PBK/TOPK | 100.2 | [96.9; 103.4] | 102.2 | [100.3; 104] |
| PDGFRa | 10.5 | [8.7; 12.4] | 46.4 | [45.4; 47.5] |
| PDGFRb | 6.1 | [4.8; 7.5] | 24.5 | [23.9; 25.2] |
| PDK1/PDPK1 | 30.7 | [28.5; 32.8] | 85.5 | [81.1; 89.8] |
| PHKg1 | 61.5 | [57.9; 65.1] | 97.1 | [93.3; 100.9] |
| PHKg2 | 93.4 | [90.7; 96.2] | 101.5 | [101.3; 101.8] |
| PIM1 | 99.2 | [98.3; 100.1] | 100.7 | [100.4; 101] |
| PIM2 | 95.6 | [93.3; 97.9] | 97.2 | [92.8; 101.6] |
| PIM3 | 82.8 | [81.5; 84] | 98.8 | [97.3; 100.3] |
| PKA | 50.8 | [48.4; 53.2] | 96.4 | [95.6; 97.3] |
| PKAcb | 70.1 | [64.1; 76.2] | 98.4 | [88.9; 107.8] |
| PKAcg | 91.7 | [84.7; 98.7] | 105.5 | [100.4; 110.6] |
| PKCa | 58.3 | [56.1; 60.6] | 92.3 | [89.3; 95.3] |
| PKCb1 | 63.0 | [61.3; 64.8] | 104.2 | [98.5; 109.9] |
| PKCb2 | 30.7 | [26.7; 34.7] | 87.7 | [79.3; 96.1] |
| PKCd | 97.7 | [95.8; 99.6] | 110.5 | [109.6; 111.5] |
| PKCepsilon | 95.0 | [92.6; 97.4] | 106.5 | [106.1; 106.9] |
| PKCeta | 96.6 | [95.9; 97.2] | 108.6 | [105.2; 112.1] |
| PKCg | 63.6 | [62.2; 65] | 102.7 | [100.4; 104.9] |
| PKCiota | 103.1 | [100.2; 105.9] | 101.5 | [99.9; 103.1] |
| PKCmu/PRKD1 | 82.1 | [80; 84.1] | 98.5 | [95; 102] |
| PKCnu/PRKD3 | 72.8 | [70.3; 75.3] | 99.9 | [97.7; 102.2] |
| PKCtheta | 99.5 | [99.4; 99.6] | 102.2 | [102.1; 102.3] |
| PKCzeta | 104.9 | [103.9; 105.8] | 108.6 | [107.9; 109.4] |
| PKD2/PRKD2 | 75.2 | [73.8; 76.6] | 102.4 | [100.5; 104.2] |
| PKG1a | 85.4 | [82.3; 88.4] | 94.8 | [88.7; 100.8] |
| PKG1b | 98.6 | [95.9; 101.3] | 93.9 | [93.8; 94] |
| PKG2/PRKG2 | 89.9 | [87.4; 92.4] | 94.2 | [92; 96.4] |
| PKN1/PRK1 | 62.6 | [59.7; 65.4] | 103.8 | [97.5; 110.2] |
| PKN2/PRK2 | 85.4 | [76.2; 94.5] | 95.6 | [92.1; 99.1] |
| PKN3/PRK3 | 40.8 | [40.5; 41.1] | 90.0 | [89; 91.1] |
| PLK1 | 60.4 | [57.8; 63] | 93.4 | [92.5; 94.3] |
| PLK2 | 19.4 | [18; 20.9] | 81.6 | [75.7; 87.5] |
| PLK3 | 53.1 | [51.5; 54.6] | 97.6 | [96.7; 98.5] |
| PLK4/SAK | 17.2 | [15.5; 18.9] | 77.8 | [75.8; 79.7] |
| PRKX | 103.8 | [102.8; 104.8] | 114.8 | [113; 116.7] |
| PYK2 | 19.6 | [17.2; 21.9] | 63.4 | [57.7; 69.2] |
| RAF1 | 73.3 | [66.7; 80] | 91.5 | [83.3; 99.6] |
| RET | 3.4 | [2.8; 4] | 23.0 | [22; 24] |
| RIPK2 | 73.0 | [72.8; 73.3] | 84.7 | [80.3; 89.1] |
| RIPK3 | 107.4 | [106.9; 107.8] | 107.5 | [103.4; 111.5] |
| RIPK5 | 100.1 | [93.7; 106.4] | 104.5 | [94.4; 114.5] |
| ROCK1 | 87.0 | [86.8; 87.2] | 95.1 | [92.5; 97.7] |
| ROCK2 | 77.6 | [74; 81.1] | 91.1 | [88.8; 93.4] |
| RON/MST1R | 47.9 | [45; 50.9] | 98.4 | [95.3; 101.4] |
| ROS/ROS1 | 0.9 | [-0.3; 2] | 25.9 | [24.5; 27.3] |
| RSK1 | 66.9 | [64.9; 69] | 104.7 | [102.9; 106.4] |
| RSK2 | 56.9 | [54.6; 59.3] | 103.9 | [102.3; 105.4] |
| RSK3 | 52.0 | [49.5; 54.5] | 91.6 | [87.5; 95.8] |
| RSK4 | 45.6 | [44.3; 46.9] | 99.7 | [99; 100.5] |
| SGK1 | 109.0 | [106.9; 111.1] | 107.5 | [105.1; 109.9] |
| SGK2 | 105.8 | [104.6; 107] | 109.6 | [105.9; 113.2] |
| SGK3/SGKL | 100.1 | [99.9; 100.3] | 103.3 | [98.7; 108] |
| SIK1 | 7.2 | [6.5; 7.9] | 10.1 | [8.9; 11.3] |
| SIK2 | 8.5 | [6.4; 10.7] | 16.2 | [16.1; 16.3] |
| SIK3 | 75.4 | [70.7; 80] | 83.4 | [81.3; 85.5] |
| SLK/STK2 | 31.4 | [29.3; 33.4] | 81.0 | [80.9; 81.2] |
| SNARK/NUAK2 | 59.7 | [52.9; 66.4] | 101.9 | [99.6; 104.2] |
| SRMS | 101.2 | [101; 101.4] | 99.7 | [97.5; 101.8] |
| SRPK1 | 101.4 | [97.4; 105.4] | 108.1 | [108.06; 108.15] |
| SRPK2 | 103.6 | [101.6; 105.6] | 104.5 | [104; 104.9] |
| SSTK/TSSK6 | 98.7 | [97.7; 99.7] | 102.9 | [97.4; 108.4] |
| STK16 | 6.3 | [5.1; 7.5] | 50.6 | [50.3; 50.8] |
| STK22D/TSSK1 | 9.6 | [8.4; 10.9] | 49.9 | [49.8; 50.1] |
| STK25/YSK1 | 90.7 | [89.2; 92.1] | 105.3 | [99.4; 111.2] |
| STK32B/YANK2 | 92.0 | [90.6; 93.3] | 102.7 | [100.8; 104.6] |
| STK32C/YANK3 | 102.1 | [99; 105.2] | 115.3 | [111.8; 118.9] |
| STK33 | 41.5 | [38.8; 44.2] | 70.5 | [65.7; 75.4] |
| STK38/NDR1 | 70.4 | [62.7; 78] | 111.5 | [111.4; 111.6] |
| STK38L/NDR2 | 32.1 | [31.3; 33] | 89.6 | [88.6; 90.7] |
| STK39/STLK3 | 106.9 | [100.7; 113.1] | 107.4 | [99.5; 115.3] |
| SYK | 11.2 | [10.7; 11.7] | 57.4 | [53.4; 61.3] |
| TAK1 | 48.3 | [47.4; 49.2] | 102.7 | [102.69; 102.74] |
| TAOK1 | 78.1 | [77; 79.2] | 102.2 | [102.1; 102.2] |
| TAOK2/TAO1 | 47.4 | [46.2; 48.6] | 104.9 | [99.9; 109.9] |
| TAOK3/JIK | 77.2 | [73; 81.3] | 107.5 | [104.1; 111] |
| TBK1 | 13.9 | [13.5; 14.4] | 66.8 | [63.1; 70.4] |
| TEC | 39.4 | [34.9; 43.8] | 71.0 | [69.8; 72.2] |
| TESK1 | 85.7 | [82.6; 88.8] | 94.5 | [90.6; 98.3] |
| TGFBR2 | 97.1 | [96.6; 97.6] | 115.7 | [114.4; 117.1] |
| TIE2/TEK | 69.9 | [66.6; 73.2] | 92.9 | [92.1; 93.7] |
| TLK1 | 75.1 | [74; 76.3] | 101.3 | [97.8; 104.9] |
| TLK2 | 54.2 | [51.7; 56.7] | 95.6 | [91.4; 99.8] |
| TNIK | 83.1 | [78; 88.2] | 78.6 | [77.1; 80] |
| TNK1 | 9.6 | [9.1; 10.1] | 44.7 | [42.9; 46.4] |
| TRKA | 25.7 | [25.5; 25.8] | 82.5 | [79; 86.1] |
| TRKB | 28.0 | [27.1; 28.9] | 92.4 | [91.1; 93.6] |
| TRKC | 26.6 | [25.2; 28.1] | 84.5 | [84.3; 84.7] |
| TSSK2 | 95.2 | [91.8; 98.7] | 105.6 | [101.4; 109.7] |
| TSSK3/STK22C | 84.9 | [84.8; 85] | 99.9 | [98.1; 101.6] |
| TTBK1 | 98.5 | [92.3; 104.7] | 108.3 | [108.1; 108.5] |
| TTBK2 | 95.4 | [93.3; 97.4] | 109.4 | [109; 109.8] |
| TXK | 16.3 | [16; 16.6] | 35.4 | [35.1; 35.6] |
| TYK1/LTK | 30.2 | [27.6; 32.9] | 92.0 | [91.6; 92.3] |
| TYK2 | 10.5 | [9.1; 12] | 71.2 | [66.7; 75.8] |
| TYRO3/SKY | 94.6 | [89.3; 100] | 104.4 | [96.1; 112.7] |
| ULK1 | 8.8 | [8.2; 9.5] | 62.2 | [60.4; 64.1] |
| ULK2 | 37.9 | [37; 38.8] | 111.4 | [106.9; 115.9] |
| ULK3 | 20.3 | [17.9; 22.7] | 74.9 | [70.8; 79] |
| VRK1 | 98.8 | [97.9; 99.8] | 101.6 | [98.3; 105] |
| VRK2 | 130.9 | [124.8; 137] | 128.9 | [128.5; 129.3] |
| WEE1 | 105.2 | [102.4; 108.1] | 107.9 | [99.3; 116.5] |
| WNK1 | 103.1 | [98.3; 108] | 105.5 | [101.7; 109.3] |
| WNK2 | 95.2 | [93.6; 96.9] | 84.6 | [82.8; 86.5] |
| WNK3 | 77.7 | [74.7; 80.7] | 82.4 | [81.8; 83] |
| YES/YES1 | 10.1 | [8.1; 12.1] | 11.6 | [9.7; 13.6] |
| ZAK/MLTK | 85.4 | [84.4; 86.4] | 92.9 | [92.9; 93] |
| ZAP70 | 43.5 | [43.5; 43.6] | 93.4 | [92.4; 94.4] |
| ZIPK/DAPK3 | 40.0 | [38.8; 41.1] | 98.4 | [95.2; 101.7] |

## **Table S3.** IC_50_ values for kinases with less than 50% residual activity.

| **Kinase** | **IC_50_. nM** |
| --- | --- |
| DDR1 | 1.8 |
| YES/YES1 | 3.5 |
| MLK2/MAP3K10 | 4.3 |
| PDGFRb | 4.5 |
| MLK1/MAP3K9 | 7.8 |
| LYN | 8.8 |
| FGR | 9.0 |
| BLK | 9.6 |
| RET | 13.5 |
| LYN B | 14.2 |
| FYN | 20.0 |
| LCK | 20.1 |
| FLT3 | 23.0 |
| SIK2 | 23.4 |
| EPHB1 | 24.7 |
| MLK3/MAP3K11 | 25.6 |
| NEK11 | 26.1 |
| ROS/ROS1 | 34.7 |
| PDGFRa | 35.9 |
| SIK1 | 35.9 |
| LIMK1 | 44.6 |
| HCK | 53.7 |
| FLT4/VEGFR3 | 55.4 |
| JAK2 | 67.2 |
| ACK1 | 85.7 |
| ABL2/ARG | 119.7 |
| STK22D/TSSK1 | 197.8 |
| BMX/ETK | 198.5 |
| LRRK2 | 206.0 |
| TXK | 221.6 |
| CSK | 238.5 |
| ARK5/NUAK1 | 376.3 |
| EPHA1 | 457.2 |

## **Table S4.** The percentage of resorption, TRAP counting and the relative resorbed area in osteoclast-mediated resorption experiment after MT-SYK-03 treatment.

| **Condition** | **Resorption** | **TRAP** | **Ratio** | |
| --- | --- | --- | --- | --- |
|  | **% resorption** | **Number of osteoclasts (Nb OC)** | **resorption/Nb OC** | **%** |
| Ctrl+ B11 | 31.62 | 533 | 0.059 | 0.754 |
| Ctrl+ C11 | 31.30 | 501 | 0.062 | 0.794 |
| Ctrl+ D11 | 34.42 | 515 | 0.067 | 0.849 |
| Ctrl+ E11 | 33.14 | 401 | 0.083 | 1.050 |
| **Average** | 32.62 | 488 | 0.068 | 0.861 |
| **EC** | 1.16 | 43 | 0.007 | 0.094 |
| vehicle B10 | 32.58 | 442 | 0.074 | 0.936 |
| vehicle C10 | 33.68 | 449 | 0.075 | 0.953 |
| vehicle D10 | 38.01 | 441 | 0.086 | 1.095 |
| vehicle E10 | 37.84 | 473 | 0.080 | 1.016 |
| **Average** | 35.53 | 451 | 0.079 | 1.000 |
| **EC** | 2.40 | 11 | 0.004 | 0.055 |
| MT-SYK-03 (0.12 μM) B1 | 34.41 | 389 | 0.088 | 1.124 |
| MT-SYK-03 (0.12 μM) C1 | 30.62 | 408 | 0.075 | 0.953 |
| MT-SYK-03 (0.12 μM) D1 | 34.06 | 402 | 0.085 | 1.076 |
| MT-SYK-03 (0.12 μM) E1 | 28.02 | 419 | 0.067 | 0.849 |
| **Average** | 31.78 | 405 | 0.079 | 1.001 |
| **EC** | 2.46 | 9 | 0.008 | 0.099 |
| MT-SYK-03 (0.37 μM) B2 | 33.11 | 394 | 0.084 | 1.067 |
| MT-SYK-03 (0.37 μM) C2 | 27.51 | 426 | 0.065 | 0.820 |
| MT-SYK-03 (0.37 μM) D2 | 32.94 | 478 | 0.069 | 0.875 |
| MT-SYK-03 (0.37 μM) E2 | 12.15 | 391 | 0.031 | 0.395 |
| **Average** | 26.43 | 422 | 0.062 | 0.789 |
| **EC** | 7.14 | 30 | 0.016 | 0.197 |
| MT-SYK-03 (1.1 μM) B3 | 14.81 | 453 | 0.033 | 0.415 |
| MT-SYK-03 (1.1 μM) C3 | 19.78 | 420 | 0.047 | 0.598 |
| MT-SYK-03 (1.1 μM) D3 | 20.80 | 397 | 0.052 | 0.665 |
| MT-SYK-03 (1.1 μM) E3 | 24.41 | 327 | 0.075 | 0.948 |
| **Average** | 19.95 | 399 | 0.052 | 0.657 |
| **EC** | 2.66 | 37 | 0.012 | 0.150 |
| MT-SYK-03 (3.3 μM) B4 | 2.31 | 165 | 0.014 | 0.178 |
| MT-SYK-03 (3.3 μM) C4 | 3.40 | 136 | 0.025 | 0.318 |
| MT-SYK-03 (3.3 μM) D4 | 2.62 | 247 | 0.011 | 0.135 |
| MT-SYK-03 (3.3 μM) E4 | 2.91 | 235 | 0.012 | 0.157 |
| **Average** | 2.81 | 196 | 0.015 | 0.197 |
| **EC** | 0.35 | 45 | 0.005 | 0.060 |
| MT-SYK-03 (10 μM) B5 | 5.87 | 371 | 0.016 | 0.201 |
| MT-SYK-03 (10 μM) C5 | 9.73 | 402 | 0.024 | 0.307 |
| MT-SYK-03 (10 μM) D5 | 8.76 | 388 | 0.023 | 0.287 |
| MT-SYK-03 (10 μM) E5 | 11.00 | 390 | 0.028 | 0.358 |
| MT-SYK-03 (10 μM) B5 | 8.84 | 388 | 0.023 | 0.288 |
| **Average** | 1.53 | 8 | 0.004 | 0.044 |
| **EC** | 5.87 | 371 | 0.016 | 0.201 |

## **Table S5.** Effect of MT-SYK-03 on chondrocyte hypertrophic-like changes induced *in vitro* by IL-1β. Type II collagen and aggrecan expression was evaluated in chondrocytes treated with or without MT-SYK-03 along with IL-1β.

| **Condition** | **Mean Ct values** | | **Relative fold expression** | | **SD** | | **P value**  **(statistical significance of difference vs vehicle)** | |
| --- | --- | --- | --- | --- | --- | --- | --- | --- |
|  | **type II collagen** | **aggrecan** | **type II collagen** | **aggrecan** | **type II collagen** | **aggrecan** | **type II collagen** | **aggrecan** |
| control | 16.21 | 22.54 | 1 | 1 | N/A | N/A | N/A | N/A |
|  | 15.96 | 21.98 |  |  |  |  |  |  |
|  | 16.11 | 22.49 |  |  |  |  |  |  |
| IL-1β | 18.86 | 25.42 | 0.11 | 0.12 | 0.02923 | 0.03675 | N/A | N/A |
|  | 18.20 | 24.35 |  |  |  |  |  |  |
|  | 19.06 | 25.21 |  |  |  |  |  |  |
| IL-1β + DMSO 0.3% | 18.56 | 24.91 | 0.09 | 0.08 | 0.01817 | 0.02171 | N/A | N/A |
|  | 18.70 | 24.98 |  |  |  |  |  |  |
|  | 18.63 | 25.46 |  |  |  |  |  |  |
| IL-1β +  MT-SYK-03  10 μM | 19.86 | 24.19 | 0.09 | 0.46 | 0.04694 | 0.23019 | 0.9445 | 0.101 |
|  | 21.17 | 25.11 |  |  |  |  |  |  |
|  | 19.55 | 23.61 |  |  |  |  |  |  |
| IL-1β +  MT-SYK-03  2 μM | 18.58 | 23.09 | 0.12 | 0.42 | 0.05002 | 0.16 | 0.561 | 0.0155 |
|  | 19.85 | 24.18 |  |  |  |  |  |  |
|  | 18.48 | 23.32 |  |  |  |  |  |  |
| IL-1β +  MT-SYK-03  0.4 μM | 18.10 | 23.61 | 0.12 | 0.20 | 0.02279 | 0.04708 | 0.017 | 0.001 |
|  | 18.35 | 24.13 |  |  |  |  |  |  |
|  | 18.56 | 24.24 |  |  |  |  |  |  |

**Table S6.** Individual and mean plasma concentration-time data of 100 mg/kg MT-SYK-03 after PO administration in male Wistar rats.

| **Sampling**  **time**  **(h)** | **Concentration** | | | | | | **Mean**  **(ng/mL)** | **SD** | **CV (%)** |
| --- | --- | --- | --- | --- | --- | --- | --- | --- | --- |
|  | **(ng/mL)** | | | | | |  |  |  |
|  | **Rat#1** | **Rat#2** | **Rat#3** | **Rat#4** | **Rat#5** | **Rat#6** |  |  |  |
| 0.25 | 257 | 127 | 313 | 251 | 240 | 283 | 245 | 63.6 | 25.9 |
| 0.5 | 331 | 166 | 419 | 280 | 345 | 375 | 319 | 88.2 | 27.6 |
| 1 | 470 | 220 | 487 | 367 | 392 | 483 | 403 | 103 | 25.5 |
| 2 | 529 | 209 | 501 | 372 | 256 | 359 | 371 | 128 | 34.4 |
| 4 | 196 | 78.0 | 171 | 170 | 226 | 206 | 175 | 51.9 | 29.7 |
| 8 | 47.2 | 31.2 | 92.6 | 41.6 | 88.8 | 43.5 | 57.5 | 26.3 | 45.8 |
| 24 | 0.818 | BQL | BQL | 17.7 | 1.57 | 16.5 | 9.15 | 9.20 | 101 |
| 48 | 0.984 | BQL | 1.55 | BQL | BQL | BQL | 1.27 | NA | NA |
| **PK parameters** | **Rat#1** | **Rat#2** | **Rat#3** | **Rat#4** | **Rat#5** | **Rat#6** | **Mean** | **SD** | **CV (%)** |
| T_max_, hr | 2.00 | 1.00 | 2.00 | 2.00 | 1.00 | 1.00 | 1.50 | 0.548 | 36.5 |
| C_max_, ng/mL | 529 | 220 | 501 | 372 | 392 | 483 | 416 | 114 | 27.5 |
| Terminal t_1/2_, h | 7.83 | 2.28 | 6.60 | 7.22 | 2.78 | 6.45 | 5.53 | 2.38 | 43.0 |
| AUC_last_, h∙ng/mL | 2423 | 869 | 3933 | 2069 | 2446 | 2297 | 2339 | 979 | 41.9 |
| AUC_INF_, h∙ng/mL | 2434 | 971 | 3948 | 2253 | 2452 | 2451 | 2418 | 945 | 39.1 |

**Table S7.** Individual and mean plasma concentration-time data of 100 mg/kg MT-SYK-03 after PO administration in male Wistar rats.

| **Sampling**  **time**  **(h)** | **Concentration** | | | | | | **Mean**  **(ng/mL)** | **SD** | **CV (%)** |
| --- | --- | --- | --- | --- | --- | --- | --- | --- | --- |
|  | **(ng/mL)** | | | | | |  |  |  |
|  | **Rat#7** | **Rat#8** | **Rat#9** | **Rat#10** | **Rat#11** | **Rat#12** |  |  |  |
| 0.25 | 400 | 401 | 436 | 404 | 463 | 388 | 415 | 28.3 | 6.82 |
| 0.5 | 716 | 654 | 612 | 677 | 709 | 707 | 679 | 40.4 | 5.95 |
| 1 | 830 | 1060 | 760 | 879 | 883 | 805 | 870 | 104.2 | 12.0 |
| 2 | 519 | 687 | 576 | 603 | 618 | 608 | 602 | 54.9 | 9.13 |
| 4 | 327 | 399 | 254 | 397 | 533 | 391 | 384 | 92.5 | 24.1 |
| 8 | 87.7 | 152 | 64.3 | 178 | 89.2 | 82.6 | 109 | 45.1 | 41.3 |
| 24 | 32.8 | 4.94 | 6.04 | 1.11 | 1.56 | 1.54 | 8.00 | 12.3 | 154 |
| 48 | 1.74 | 0.932 | BQL | BQL | BQL | 2.71 | 1.79 | 0.890 | 49.6 |
| **PK parameters** | **Rat#7** | **Rat#8** | **Rat#9** | **Rat#10** | **Rat#11** | **Rat#12** | **Mean** | **SD** | **CV (%)** |
| T_max_, h | 1.00 | 1.00 | 1.00 | 1.00 | 1.00 | 1.00 | 1.00 | 0.00 | 0.00 |
| C_max_, ng/mL | 822 | 1030 | 770 | 859 | 876 | 824 | 864 | 89.4 | 10.3 |
| Terminal t_1/2_, h | 5.58 | 4.86 | 3.71 | 2.36 | 2.34 | 5.52 | 4.06 | 1.49 | 36.6 |
| AUC_last_, h∙ng/mL | 4237 | 4843 | 3238 | 4878 | 4452 | 3910 | 4260 | 621 | 14.6 |
| AUC_INF_, h∙ng/mL | 4247 | 4850 | 3276 | 4883 | 4456 | 3930 | 4273 | 608 | 14.2 |

**Table S8.** Individual and mean plasma concentration-time data of 200 mg/kg MT-SYK-03 after PO administration in male Wistar rats.

| **Sampling**  **time**  **(h)** | **Concentration** | | | | | | **Mean**  **(ng/mL)** | **SD** | **CV (%)** |
| --- | --- | --- | --- | --- | --- | --- | --- | --- | --- |
|  | **(ng/mL)** | | | | | |  |  |  |
|  | **Rat#13** | **Rat#14** | **Rat#15** | **Rat#16** | **Rat#17** | **Rat#18** |  |  |  |
| 0.25 | 489 | 521 | 371 | 446 | 365 | 355 | 425 | 70.9 | 16.7 |
| 0.5 | 858 | 1010 | 1030 | 881 | 767 | 804 | 892 | 107 | 12.0 |
| 1 | 984 | 1430 | 1360 | 1370 | 998 | 1170 | 1219 | 197 | 16.1 |
| 2 | 1180 | 924 | 1210 | 863 | 728 | 1320 | 1038 | 232 | 22.4 |
| 4 | 840 | 470 | 681 | 692 | 693 | 972 | 725 | 169 | 23.4 |
| 8 | 190 | 163 | 146 | 225 | 151 | 358 | 206 | 80.2 | 39.0 |
| 24 | 4.40 | 1.85 | 2.41 | 2.18 | 2.62 | 16.5 | 4.99 | 5.71 | 114 |
| 48 | 1.82 | 0.916 | BQL | BQL | 17.9 | 1.14 | 5.44 | 8.31 | 153 |
| **PK parameters** | **Rat#13** | **Rat#14** | **Rat#15** | **Rat#16** | **Rat#17** | **Rat#18** | **Mean** | **SD** | **CV (%)** |
| T_max_, h | 2.00 | 1.00 | 1.00 | 1.00 | 1.00 | 2.00 | 1.33 | 0.516 | 38.7 |
| C_max_, ng/mL | 1180 | 1430 | 1360 | 1370 | 998 | 1320 | 1276 | 160 | 12.5 |
| Terminal t_1/2_, h | 6.31 | 5.70 | 2.52 | 2.40 | 8.67 | 4.91 | 5.08 | 2.39 | 47.0 |
| AUC_last_, h∙ng/mL | 7482 | 6055 | 6836 | 7107 | 6076 | 10087 | 7274 | 1490 | 20.5 |
| AUC_INF_, h∙ng/mL | 7498 | 6063 | 6845 | 7115 | 6299 | 10096 | 7319 | 1458 | 19.9 |

**Table S9.** Individual and mean bone concentration-time data of 100 mg/kg MT-SYK-03 after a PO administration in male Wistar rats.

| **Sampling time**  **(h)** | **Concentration (ng/mL)** | | | **Mean**  **(ng/mL)** | **SD** | **CV (%)** |
| --- | --- | --- | --- | --- | --- | --- |
| 2 | 327 | 433 | 576 | 445 | 125 | 28.1 |
| 8 | 1290 | 681 | 153 | 708 | 569 | 80.4 |
| 24 | 8.28 | 19.9 | 14.5 | 14.2 | 5.81 | 40.9 |
| **PK parameters** | Estimated Value | | | | | |
| T_max_, h | 8.00 | | | | | |
| C_max_, ng/mL | 708 | | | | | |
| Terminal t_1/2_, h | NA | | | | | |
| AUC_24_, h∙ng/mL | 9683 | | | | | |
| AUC_INF_, h∙ng/mL | NA | | | | | |
| AUC_24_/AUC_24_ (plasma) | 2.50 | | | | | |

**Table S10.** Individual and mean joint concentration-time data of 100 mg/kg MT-SYK-03 after PO administration in male Wistar rats.

| **Sampling time**  **(h)** | **Concentration (ng/mL)** | | | **Mean**  **(ng/mL)** | **SD** | **CV (%)** |
| --- | --- | --- | --- | --- | --- | --- |
| 2 | 470 | 584 | 631 | 562 | 82.8 | 14.7 |
| 8 | 1100 | 673 | 216 | 663 | 442 | 66.7 |
| 24 | 12.2 | 20.4 | 8.49 | 13.7 | 6.09 | 44.5 |
| **PK parameters** | Estimated Value | | | | | |
| T_max_, h | 8.00 | | | | | |
| C_max_, ng/mL | 663 | | | | | |
| Terminal t_1/2_, h | NA | | | | | |
| AUC_24_, h∙ng/mL | 9649 | | | | | |
| AUC_INF_, h∙ng/mL | NA | | | | | |
| AUC_24_/AUC_24_ (plasma) | 2.49 | | | | | |

**Table S11.** Individual and mean spleen concentration-time data of 100 mg/kg MT-SYK-03 after PO administration in male Wistar rats.

| **Sampling time**  **(hr)** | **Concentration (ng/mL)** | | | **Mean**  **(ng/mL)** | **SD** | **CV (%)** |
| --- | --- | --- | --- | --- | --- | --- |
| 2 | 2060 | 1420 | 2260 | 1913 | 439 | 22.9 |
| 8 | 726 | 129 | 268 | 374 | 312 | 83.5 |
| 24 | BQL | 30.3 | BQL | 30.3 | NA | NA |
| **PK parameters** | Estimated Value | | | | | |
| T_max_, hr | 2.00 | | | | | |
| C_max_, ng/mL | 1913 | | | | | |
| Terminal t_1/2_, hr | 3.81 | | | | | |
| AUC_24_, h∙ng/mL | 12013 | | | | | |
| AUC_INF_, h∙ng/mL | 12180 | | | | | |
| AUC_24_/AUC_24_ (plasma) | 3.10 | | | | | |

**Table S12.** Individual and mean muscle concentration-time data of 100 mg/kg MT-SYK-03 after PO administration in male Wistar rats.

| **Sampling time**  **(h)** | **Concentration (ng/mL)** | | | **Mean**  **(ng/mL)** | **SD** | **CV (%)** |
| --- | --- | --- | --- | --- | --- | --- |
| 2 | 456 | 435 | 416 | 436 | 20.0 | 4.59 |
| 8 | 160 | 107 | 93.2 | 120 | 35.3 | 29.4 |
| 24 | 4.68 | 8.92 | BQL | 6.80 | NA | NA |
| **PK parameters** | Estimated Value | | | | | |
| T_max_, h | 2.00 | | | | | |
| C_max_, ng/mL | 436 | | | | | |
| Terminal t_1/2_, h | 3.70 | | | | | |
| AUC_24_, h∙ng/mL | 3118 | | | | | |
| AUC_INF_, h∙ng/mL | 3154 | | | | | |
| AUC_24_/AUC_24_ (plasma) | 0.805 | | | | | |

**Table S13.** Individual and mean liver concentration-time data of 100 mg/kg MT-SYK-03 after PO administration in male Wistar rats.

| **Sampling time**  **(h)** | **Concentration (ng/mL)** | | | **Mean**  **(ng/mL)** | **SD** | **CV (%)** |
| --- | --- | --- | --- | --- | --- | --- |
| 2 | 4450 | 4700 | 6610 | 5253 | 1182 | 22.5 |
| 8 | 1860 | 635 | 442 | 979 | 769 | 78.6 |
| 24 | 7.95 | 115 | 12.8 | 45.3 | 60.5 | 134 |
| **PK parameters** | Estimated Value | | | | | |
| T_max_, h | 2.00 | | | | | |
| C_max_, ng/mL | 5253 | | | | | |
| Terminal t_1/2_, h | 3.28 | | | | | |
| AUC_24_, h∙ng/mL | 32144 | | | | | |
| AUC_INF_, h∙ng/mL | 32359 | | | | | |
| AUC_24_/AUC_24_ (plasma) | 8.30 | | | | | |

**Table S14.** Individual and mean kidney concentration-time data of 100 mg/kg MT-SYK-03 after PO administration in male Wistar rats.

| **Sampling time**  **(h)** | **Concentration (ng/mL)** | | | **Mean**  **(ng/mL)** | **SD** | **CV (%)** |
| --- | --- | --- | --- | --- | --- | --- |
| 2 | 2350 | 2880 | 3160 | 2797 | 411 | 14.7 |
| 8 | 1200 | 375 | 260 | 612 | 513 | 83.8 |
| 24 | 5.30 | 72.0 | 8.51 | 28.6 | 37.6 | 132 |
| **PK parameters** | Estimated Value | | | | | |
| T_max_, h | 2.00 | | | | | |
| C_max_, ng/mL | 2797 | | | | | |
| Terminal t_1/2_, h | 3.38 | | | | | |
| AUC_24_, h∙ng/mL | 18144 | | | | | |
| AUC_INF_, h∙ng/mL | 18283 | | | | | |
| AUC_24_/AUC_24_ (plasma) | 4.68 | | | | | |

**Table S15.** Individual and mean prostate concentration-time data of 100 mg/kg MT-SYK-03 after PO administration in male Wistar rats.

| **Sampling time**  **(h)** | **Concentration (ng/mL)** | | | **Mean**  **(ng/mL)** | **SD** | **CV (%)** |
| --- | --- | --- | --- | --- | --- | --- |
| 2 | 1070 | 1200 | 1500 | 1257 | 221 | 17.5 |
| 8 | 500 | 154 | 98.0 | 251 | 218 | 86.9 |
| 24 | BQL | 21.6 | BQL | 21.6 | NA | NA |
| **PK parameters** | Estimated Value | | | | | |
| T_max_, h | 2.00 | | | | | |
| C_max_, ng/mL | 1257 | | | | | |
| Terminal t_1/2_, h | 3.89 | | | | | |
| AUC_24_, h∙ng/mL | 7957 | | | | | |
| AUC_INF_, h∙ng/mL | 8078 | | | | | |
| AUC_24_/AUC_24_ (plasma) | 2.05 | | | | | |

**Table S16.** Individual Animal Summary of Paw Score (All Paws)

| **Treatment group** | **Average Paw Score Per Arthritis Day** | | | | | | | | | | | **Total AUC** |
| --- | --- | --- | --- | --- | --- | --- | --- | --- | --- | --- | --- | --- |
| **Group 1**  **Naïve** | **Day 1** | **Day 2** | **Day 3** | **Day 4** | **Day 5** | **Day 6** | **Day 7** | **Day 8** | **Day 9** | **Day 10** | **Day 11** | **Sum (d1-11)** |
| 1 | 0.00 | 0.00 | 0.00 | 0.00 | 0.00 | 0.00 | 0.00 | 0.00 | 0.00 | 0.00 | 0.00 | 0.00 |
| 2 | 0.00 | 0.00 | 0.00 | 0.00 | 0.00 | 0.00 | 0.00 | 0.00 | 0.00 | 0.00 | 0.00 | 0.00 |
| 3 | 0.00 | 0.00 | 0.00 | 0.00 | 0.00 | 0.00 | 0.00 | 0.00 | 0.00 | 0.00 | 0.00 | 0.00 |
| 4 | 0.00 | 0.00 | 0.00 | 0.00 | 0.00 | 0.00 | 0.00 | 0.00 | 0.00 | 0.00 | 0.00 | 0.00 |
| Mean | 0.00 | 0.00 | 0.00 | 0.00 | 0.00 | 0.00 | 0.00 | 0.00 | 0.00 | 0.00 | 0.00 | 0.00 |
| SE | 0.00 | 0.00 | 0.00 | 0.00 | 0.00 | 0.00 | 0.00 | 0.00 | 0.00 | 0.00 | 0.00 | 0.002 |
| t-test to Vehicle | 0.001 | 0.002 | 0.002 | 0.002 | 0.001 | 0.002 | 0.001 | 0.001 | 0.001 | 0.001 | 0.001 | 100% |
| % inhibition to Vehicle | 100% | 100% | 100% | 100% | 100% | 100% | 100% | 100% | 100% | 100% | 100% | 0.00 |
| **Group 2**  **Vehicle Control** | **Average Paw Score Per Arthritis Day** | | | | | | | | | | | **Total AUC** |
|  | **Day 1** | **Day 2** | **Day 3** | **Day 4** | **Day 5** | **Day 6** | **Day 7** | **Day 8** | **Day 9** | **Day 10** | **Day 11** | **Sum (d1-11)** |
| 1 | 1.00 | 2.50 | 3.25 | 4.00 | 4.75 | 5.00 | 5.00 | 5.00 | 5.00 | 5.00 | 5.00 | 42.50 |
| 2 | 1.50 | 2.75 | 3.50 | 3.75 | 4.00 | 4.00 | 4.25 | 4.25 | 4.50 | 4.50 | 4.50 | 38.50 |
| 3 | 0.50 | 1.25 | 1.50 | 2.00 | 2.25 | 2.25 | 2.25 | 2.50 | 2.50 | 2.75 | 2.75 | 20.88 |
| 4 | 0.50 | 1.00 | 1.50 | 2.00 | 2.00 | 2.25 | 2.50 | 2.75 | 2.75 | 3.00 | 3.00 | 21.50 |
| 5 | 0.50 | 1.75 | 3.00 | 3.50 | 4.25 | 4.25 | 4.50 | 4.50 | 4.75 | 5.00 | 5.00 | 38.25 |
| 6 | 1.00 | 1.50 | 3.00 | 3.50 | 4.25 | 4.25 | 4.50 | 4.50 | 4.75 | 5.00 | 5.00 | 38.25 |
| 7 | 0.50 | 1.25 | 2.00 | 2.25 | 2.50 | 2.50 | 2.50 | 2.50 | 2.50 | 2.50 | 2.50 | 22.00 |
| 8 | 1.00 | 2.50 | 3.50 | 4.00 | 4.00 | 4.00 | 4.00 | 4.25 | 4.50 | 4.50 | 4.50 | 38.00 |
| 9 | 0.50 | 2.25 | 3.25 | 4.00 | 4.00 | 4.25 | 4.25 | 4.25 | 4.50 | 4.50 | 4.50 | 37.75 |
| 10 | 1.00 | 1.50 | 2.00 | 2.50 | 2.75 | 2.75 | 2.75 | 3.25 | 3.75 | 3.75 | 3.75 | 27.38 |
| Mean | 0.80 | 1.83 | 2.65 | 3.15 | 3.48 | 3.55 | 3.65 | 3.78 | 3.95 | 4.05 | 4.05 | 32.50 |
| SE | 0.11 | 0.20 | 0.26 | 0.27 | 0.31 | 0.32 | 0.33 | 0.29 | 0.32 | 0.31 | 0.31 | 2.69 |
| % inhibition to Vehicle | 0% | 0% | 0% | 0% | 0% | 0% | 0% | 0% | 0% | 0% | 0% | 0% |

**Table S16.** Individual Animal Summary of Paw Score (All Paws) (continued)

| **Group 3**  **MTX (1.5 mg/kg) PO, QD** | **Average Paw Score Per Arthritis Day** | | | | | | | | | | | **Total AUC** |
| --- | --- | --- | --- | --- | --- | --- | --- | --- | --- | --- | --- | --- |
|  | **Day 1** | **Day 2** | **Day 3** | **Day 4** | **Day 5** | **Day 6** | **Day 7** | **Day 8** | **Day 9** | **Day 10** | **Day 11** | **Sum (d1-11)** |
| 1 | 0.50 | 1.00 | 2.00 | 2.50 | 3.25 | 3.25 | 3.50 | 3.75 | 4.00 | 4.75 | 4.75 | 30.63 |
| 2 | 0.50 | 1.00 | 2.50 | 3.50 | 4.00 | 4.00 | 4.00 | 4.00 | 4.25 | 4.50 | 4.50 | 34.25 |
| 3 | 0.50 | 2.00 | 2.75 | 3.25 | 3.50 | 3.50 | 3.50 | 3.50 | 3.50 | 3.50 | 3.50 | 31.00 |
| 4 | 1.00 | 1.75 | 2.00 | 2.75 | 2.75 | 2.75 | 2.75 | 2.75 | 2.75 | 2.75 | 2.75 | 24.88 |
| 5 | 1.00 | 2.50 | 3.00 | 3.50 | 3.50 | 3.50 | 3.50 | 3.50 | 3.75 | 4.00 | 4.00 | 33.25 |
| 6 | 1.50 | 2.00 | 2.75 | 4.00 | 4.75 | 4.75 | 4.75 | 4.75 | 5.00 | 5.00 | 5.00 | 41.00 |
| 7 | 0.50 | 0.50 | 0.75 | 1.75 | 2.25 | 2.50 | 2.50 | 2.50 | 2.50 | 2.50 | 2.50 | 19.25 |
| 8 | 1.00 | 1.25 | 1.75 | 3.00 | 3.25 | 3.25 | 3.50 | 3.50 | 3.50 | 3.50 | 3.50 | 28.75 |
| 9 | 0.50 | 1.25 | 1.75 | 2.00 | 2.00 | 2.00 | 2.00 | 2.00 | 2.00 | 2.00 | 2.00 | 18.25 |
| 10 | 1.00 | 1.50 | 2.00 | 2.25 | 2.25 | 2.25 | 2.25 | 2.50 | 3.00 | 3.25 | 3.25 | 23.38 |
| Mean | 0.80 | 1.48 | 2.13 | 2.85 | 3.15 | 3.18 | 3.23 | 3.28 | 3.43 | 3.58 | 3.58 | 28.46 |
| SE | 0.11 | 0.19 | 0.21 | 0.23 | 0.27 | 0.26 | 0.27 | 0.26 | 0.28 | 0.31 | 0.31 | 2.24 |
| ANOVA Results (post-test to grp 2) | >0.9999 | 0.904 | 0.685 | >0.9999 | >0.9999 | >0.9999 | >0.9999 | 0.849 | 0.861 | >0.9999 | >0.9999 | 0.926 |
| % inhibition to Vehicle | 0% | 19% | 20% | 10% | 9% | 11% | 12% | 13% | 13% | 12% | 12% | 12% |
| **Group 4**  MT-SYK-03 (103 mg/kg) PO, BID | **Average Paw Score Per Arthritis Day** | | | | | | | | | | | **Total AUC** |
|  | **Day 1** | **Day 2** | **Day 3** | **Day 4** | **Day 5** | **Day 6** | **Day 7** | **Day 8** | **Day 9** | **Day 10** | **Day 11** | **Sum (d1-11)** |
| 1 | 0.50 | 1.25 | 2.25 | 2.75 | 3.00 | 3.00 | 3.00 | 3.25 | 3.25 | 3.50 | 3.50 | 27.25 |
| 2 | 1.50 | 2.00 | 3.00 | 3.25 | 3.50 | 3.50 | 3.50 | 3.50 | 3.75 | 3.75 | 3.75 | 32.38 |
| 3 | 0.50 | 1.00 | 1.25 | 2.00 | 2.75 | 3.00 | 3.25 | 3.25 | 3.50 | 3.75 | 4.00 | 26.00 |
| 4 | 1.00 | 2.50 | 3.25 | 3.50 | 3.75 | 3.75 | 4.00 | 4.00 | 4.25 | 4.25 | 4.25 | 35.88 |
| 5 | 0.50 | 1.00 | 1.25 | 1.25 | 1.25 | 1.25 | 1.25 | 1.25 | 1.50 | 1.50 | 1.50 | 12.50 |
| 6 | 0.50 | 1.25 | 2.00 | 2.75 | 3.50 | 3.50 | 3.50 | 3.50 | 3.50 | 3.50 | 3.50 | 29.00 |
| 7 | 1.00 | 2.00 | 3.00 | 3.50 | 4.00 | 4.00 | 4.25 | 4.50 | 4.50 | 4.75 | 4.75 | 37.38 |
| 8 | 1.00 | 2.00 | 2.75 | 3.50 | 3.50 | 3.50 | 3.75 | 3.75 | 3.75 | 4.00 | 4.00 | 33.00 |
| 9 | 1.00 | 1.50 | 2.50 | 3.00 | 3.50 | 4.00 | 4.00 | 4.25 | 4.25 | 4.25 | 4.25 | 33.88 |
| 10 | 0.50 | 0.50 | 1.25 | 2.00 | 2.00 | 2.25 | 2.25 | 2.00 | 2.00 | 2.00 | 2.00 | 17.50 |
| Mean | 0.80 | 1.50 | 2.25 | 2.75 | 3.08 | 3.18 | 3.28 | 3.33 | 3.43 | 3.53 | 3.55 | 28.48 |
| SE | 0.11 | 0.19 | 0.25 | 0.24 | 0.27 | 0.27 | 0.29 | 0.32 | 0.31 | 0.32 | 0.32 | 2.54 |
| ANOVA Results (post-test to grp 2) | >0.9999 | >0.9999 | >0.9999 | >0.9999 | >0.9999 | >0.9999 | >0.9999 | >0.9999 | >0.9999 | >0.9999 | >0.9999 | >0.9999 |
| % inhibition to Vehicle | 0% | 18% | 15% | 13% | 12% | 11% | 10% | 12% | 13% | 13% | 12% | 12% |

**Table S16.** Individual Animal Summary of Paw Score (All Paws) (continued)

| **Group 5**  MT-SYK-03 + MTX (52 + 1.5 mg/kg)  PO, BID (MTX is QD) | **Average Paw Score Per Arthritis Day** | | | | | | | | | | | **Total AUC** |
| --- | --- | --- | --- | --- | --- | --- | --- | --- | --- | --- | --- | --- |
|  | **Day 1** | **Day 2** | **Day 3** | **Day 4** | **Day 5** | **Day 6** | **Day 7** | **Day 8** | **Day 9** | **Day 10** | **Day 11** | **Sum (d1-11)** |
| 1 | 1.50 | 2.25 | 3.25 | 3.75 | 4.00 | 4.00 | 4.00 | 4.00 | 4.25 | 4.25 | 4.25 | 36.63 |
| 2 | 0.50 | 1.25 | 2.00 | 3.25 | 3.75 | 3.75 | 4.00 | 4.00 | 4.00 | 4.00 | 4.00 | 32.25 |
| 3 | 1.00 | 1.50 | 2.00 | 2.50 | 2.50 | 2.75 | 3.00 | 3.00 | 3.00 | 3.00 | 3.00 | 25.25 |
| 4 | 1.00 | 1.50 | 1.75 | 2.00 | 2.00 | 2.00 | 2.00 | 2.00 | 2.00 | 2.00 | 2.00 | 18.75 |
| 5 | 0.50 | 1.25 | 1.75 | 2.25 | 2.75 | 2.75 | 2.75 | 2.75 | 3.25 | 3.50 | 3.50 | 25.00 |
| 6 | 0.50 | 1.00 | 1.50 | 1.75 | 2.00 | 2.00 | 2.00 | 2.00 | 2.00 | 2.00 | 2.00 | 17.50 |
| 7 | 0.50 | 0.75 | 0.75 | 1.25 | 1.75 | 1.75 | 1.75 | 1.75 | 1.50 | 1.25 | 1.25 | 13.38 |
| 8 | 1.00 | 1.25 | 2.00 | 3.00 | 2.75 | 2.75 | 2.75 | 2.75 | 2.50 | 2.50 | 2.50 | 24.00 |
| 9 | 1.00 | 1.50 | 2.50 | 3.50 | 3.50 | 3.75 | 3.75 | 4.00 | 4.00 | 4.00 | 4.00 | 33.00 |
| 10 | 0.50 | 0.75 | 1.00 | 1.00 | 1.00 | 1.00 | 1.00 | 1.25 | 1.25 | 1.25 | 1.25 | 10.38 |
| Mean | 0.80 | 1.30 | 1.85 | 2.43 | 2.60 | 2.65 | 2.70 | 2.75 | 2.78 | 2.78 | 2.78 | 23.61 |
| SE | 0.11 | 0.14 | 0.22 | 0.30 | 0.30 | 0.31 | 0.32 | 0.32 | 0.34 | 0.36 | 0.36 | 2.74 |
| ANOVA Results (post-test to grp 2) | 0.9999 | 0.250 | 0.110 | 0.247 | 0.155 | 0.144 | 0.142 | 0.094 | 0.058 | 0.052 | 0.050 | 0.084 |
| % inhibition to Vehicle | 0% | 29% | 30% | 23% | 25% | 25% | 26% | 27% | 30% | 31% | 31% | 27% |
| ANOVA Results (post-test to grp 3) | >0.9999 | >0.9999 | >0.9999 | >0.9999 | >0.9999 | >0.9999 | >0.9999 | >0.9999 | 0.908 | 0.609 | 0.600 | >0.9999 |
| % inhibition to MTX | 0% | 12% | 13% | 15% | 17% | 17% | 16% | 16% | 19% | 22% | 22% | 17% |
| **Group 6**  MT-SYK-03 + MTX (103 + 1.5 mg/kg)  PO, BID (MTX is QD) | **Average Paw Score Per Arthritis Day** | | | | | | | | | | | **Total AUC** |
|  | **Day 1** | **Day 2** | **Day 3** | **Day 4** | **Day 5** | **Day 6** | **Day 7** | **Day 8** | **Day 9** | **Day 10** | **Day 11** | **Sum (d1-11)** |
| 1 | 1.50 | 2.00 | 2.25 | 2.25 | 2.25 | 2.25 | 2.50 | 2.50 | 2.50 | 2.50 | 2.50 | 23.00 |
| 2 | 0.50 | 1.00 | 1.50 | 1.75 | 2.00 | 2.25 | 2.25 | 2.25 | 2.50 | 2.50 | 2.50 | 19.50 |
| 3 | 0.50 | 1.00 | 1.50 | 1.75 | 2.00 | 2.00 | 2.00 | 2.00 | 2.00 | 2.00 | 2.00 | 17.50 |
| 4 | 0.50 | 1.50 | 2.00 | 2.25 | 2.50 | 2.50 | 2.50 | 2.75 | 2.75 | 3.00 | 3.00 | 23.50 |
| 5 | 0.50 | 0.50 | 0.50 | 1.00 | 1.25 | 1.25 | 1.25 | 1.25 | 1.00 | 1.00 | 1.00 | 9.75 |
| 6 | 1.00 | 1.50 | 2.50 | 3.50 | 4.00 | 4.00 | 4.00 | 4.00 | 4.00 | 4.00 | 4.00 | 34.00 |
| 7 | 1.00 | 1.00 | 1.50 | 1.75 | 1.75 | 1.75 | 1.75 | 1.75 | 1.75 | 1.50 | 1.50 | 15.75 |
| 8 | 1.00 | 2.00 | 2.75 | 3.50 | 3.75 | 4.00 | 4.00 | 4.00 | 4.00 | 4.00 | 4.00 | 34.50 |
| 9 | 0.50 | 0.50 | 0.75 | 1.00 | 1.00 | 1.00 | 1.00 | 1.00 | 1.00 | 1.00 | 1.00 | 9.00 |
| 10 | 1.00 | 1.50 | 2.00 | 2.50 | 2.50 | 2.50 | 2.50 | 2.25 | 2.00 | 2.00 | 2.00 | 21.25 |
| Mean | 0.80 | 1.25 | 1.73 | 2.13 | 2.30 | 2.35 | 2.38 | 2.38 | 2.35 | 2.35 | 2.35 | 20.78 |
| SE | 0.11 | 0.17 | 0.23 | 0.28 | 0.30 | 0.32 | 0.32 | 0.32 | 0.33 | 0.34 | 0.34 | 2.74 |
| ANOVA Results (post-test to grp 2) | >0.9999 | 0.184 | 0.056 | 0.033 | 0.026 | 0.026 | 0.021 | 0.010 | 0.005 | 0.005 | 0.005 | 0.012 |
| % inhibition to Vehicle | 0% | 32% | 35% | 33% | 34% | 34% | 35% | 37% | 41% | 42% | 42% | 36% |
| ANOVA Results (post-test to grp 3) | >0.9999 | >0.9999 | >0.9999 | 0.324 | 0.310 | 0.372 | 0.378 | 0.296 | 0.188 | 0.118 | 0.115 | 0.311 |
| % inhibition to MTX | 0% | 15% | 19% | 25% | 27% | 26% | 26% | 27% | 31% | 34% | 34% | 27% |

**Table S17.** Summary of Individual Animal Histopathology – All Joints

| **Treatment group** | **Mean of All Joints** | | | | | | |
| --- | --- | --- | --- | --- | --- | --- | --- |
| **Group 1**  **Naïve** | **Histopathology Scores** | | | | | | **Periosteal Bone Width**  **μm (40*25x measure)** |
|  | **Inflammation** | **Pannus** | **Cartilage**  **Damage** | **Bone**  **Resorption** | **Periosteal**  **Bone Formation** | **Summed**  **Scores** |  |
| 1 | 0.00 | 0.00 | 0.00 | 0.00 | 0.00 | 0.00 | 0.00  0.00  0.00  0.00  0.00  0.00  0.007  100% |
| 2 | 0.00 | 0.00 | 0.00 | 0.00 | 0.00 | 0.00 |  |
| 3 | 0.00 | 0.00 | 0.00 | 0.00 | 0.00 | 0.00 |  |
| 4 | 0.00 | 0.00 | 0.00 | 0.00 | 0.00 | 0.00 |  |
| Mean | 0.00 | 0.00 | 0.00 | 0.00 | 0.00 | 0.00 |  |
| SE | 0.00 | 0.00 | 0.00 | 0.00 | 0.00 | 0.00 |  |
| t-test to grp 2 | 0.002 | 0.001 | 0.002 | 0.001 | 0.009 | 0.002 |  |
| % inhibition | 100% | 100% | 100% | 100% | 100% | 100% |  |
| **Group 2**  **Vehicle Control** | **Histopathology Scores** | | | | | | **Periosteal Bone Width**  **μm (40*25x measure)** |
|  | **Inflammation** | **Pannus** | **Cartilage**  **Damage** | **Bone**  **Resorption** | **Periosteal**  **Bone Formation** | **Summed**  **Scores** |  |
| 1 | 5.00 | 2.83 | 5.00 | 2.83 | 2.00 | 17.67 | 173.33 |
| 2 | 4.33 | 2.25 | 3.83 | 2.25 | 1.17 | 13.83 | 93.33 |
| 3 | 2.25 | 0.50 | 1.25 | 0.50 | 0.33 | 4.83 | 26.67 |
| 4 | 2.17 | 1.33 | 1.83 | 1.33 | 1.00 | 7.67 | 86.67 |
| 5 | 4.83 | 2.50 | 4.83 | 2.50 | 1.83 | 16.50 | 146.67 |
| 6 | 4.83 | 2.42 | 4.50 | 2.42 | 1.33 | 15.50 | 113.33 |
| 7 | 1.67 | 0.17 | 0.50 | 0.17 | 0.00 | 2.50 | 0.00 |
| 8 | 4.33 | 2.25 | 4.67 | 2.25 | 2.00 | 15.50 | 186.67 |
| 9 | 4.50 | 1.50 | 3.67 | 1.50 | 0.83 | 12.00 | 80.00 |
| 10 | 3.25 | 1.50 | 3.08 | 1.50 | 0.83 | 10.17 | 80.00 |
| Mean | 3.72 | 1.73 | 3.32 | 1.73 | 1.13 | 11.62 | 98.67 |
| SE | 0.40 | 0.28 | 0.51 | 0.28 | 0.21 | 1.64 | 18.75 |
| % inhibition to Vehicle | 0% | 0% | 0% | 0% | 0% | 0% | 0% |

**Table S17.** Summary of Individual Animal Histopathology – All Joints (continued)

| **Group 3**  **MTX (1.5 mg/kg) PO, QD** | **Histopathology Scores** | | | | | | **Periosteal Bone Width**  **μm (40*25x measure)** |
| --- | --- | --- | --- | --- | --- | --- | --- |
|  | **Inflammation** | **Pannus** | **Cartilage**  **Damage** | **Bone**  **Resorption** | **Periosteal**  **Bone Formation** | **Summed**  **Scores** |  |
| 1 | 4.67 | 1.67 | 4.17 | 1.67 | 1.17 | 13.33 | 80.00 |
| 2 | 4.83 | 2.33 | 4.33 | 2.33 | 1.50 | 15.33 | 140.00 |
| 3 | 4.00 | 1.58 | 3.83 | 1.58 | 1.17 | 12.17 | 86.67 |
| 4 | 3.33 | 1.25 | 3.00 | 1.25 | 0.83 | 9.67 | 60.00 |
| 5 | 3.83 | 1.83 | 3.17 | 1.83 | 1.33 | 12.00 | 106.67 |
| 6 | 4.67 | 2.25 | 4.83 | 2.25 | 1.50 | 15.50 | 146.67 |
| 7 | 2.33 | 1.17 | 1.92 | 1.17 | 0.83 | 7.42 | 80.00 |
| 8 | 3.08 | 1.67 | 3.00 | 1.67 | 1.33 | 10.75 | 106.67 |
| 9 | 2.50 | 1.08 | 1.67 | 1.08 | 0.50 | 6.83 | 40.00 |
| 10 | 2.25 | 1.00 | 2.17 | 1.00 | 0.83 | 7.25 | 80.00 |
| Mean | 3.55 | 1.58 | 3.21 | 1.58 | 1.10 | 11.03 | 92.67 |
| SE | 0.32 | 0.15 | 0.34 | 0.15 | 0.11 | 1.02 | 10.49 |
| ANOVA Results (post-test to grp 2) | >0.9999 | >0.9999 | >0.9999 | >0.9999 | >0.9999 | >0.9999 | 0.734 |
| % inhibition | 4% | 8% | 3% | 8% | 3% | 5% | 6% |
| **Group 4**  **MT-SYK-03 (103 mg/kg) PO, BID** | **Histopathology Scores** | | | | | | **Periosteal Bone Width**  **μm (40*25x measure)** |
|  | **Inflammation** | **Pannus** | **Cartilage**  **Damage** | **Bone**  **Resorption** | **Periosteal**  **Bone Formation** | **Summed**  **Scores** |  |
| 1 | 2.83 | 0.83 | 2.33 | 0.83 | 0.67 | 7.50 | 40.00 |
| 2 | 3.17 | 0.75 | 2.00 | 0.75 | 0.67 | 7.33 | 53.33 |
| 3 | 3.83 | 1.17 | 2.58 | 1.17 | 1.17 | 9.92 | 106.67 |
| 4 | 4.67 | 1.92 | 4.00 | 1.92 | 1.33 | 13.83 | 120.00 |
| 5 | 1.92 | 0.75 | 1.67 | 0.75 | 0.33 | 5.42 | 33.33 |
| 6 | 3.42 | 1.42 | 2.75 | 1.42 | 1.17 | 10.17 | 86.67 |
| 7 | 4.50 | 2.00 | 4.17 | 2.00 | 1.17 | 13.83 | 106.67 |
| 8 | 3.83 | 1.42 | 3.25 | 1.42 | 1.67 | 11.58 | 140.00 |
| 9 | 4.33 | 1.75 | 4.00 | 1.75 | 0.83 | 12.67 | 80.00 |
| 10 | 1.92 | 0.33 | 0.67 | 0.33 | 0.33 | 3.58 | 26.67 |
| Mean | 3.44 | 1.23 | 2.74 | 1.23 | 0.93 | 9.58 | 79.33 |
| SE | 0.31 | 0.18 | 0.36 | 0.18 | 0.14 | 1.12 | 12.47 |
| ANOVA Results (post-test to grp 2) | >0.9999 | 0.777 | >0.9999 | 0.746 | >0.9999 | >0.9999 | 0.475 |
| % inhibition | 7% | 29% | 17% | 29% | 18% | 18% | 20% |

**Table S17.** Summary of Individual Animal Histopathology – All Joints (continued)

| **Group 5**  **MT-SYK-03 (52 mg/kg) + MTX (1.5 mg/kg)**  **PO, BID (MTX is QD)** | **Histopathology Scores** | | | | | | **Periosteal Bone Width**  **μm (40*25x measure)** |
| --- | --- | --- | --- | --- | --- | --- | --- |
|  | **Inflammation** | **Pannus** | **Cartilage**  **Damage** | **Bone**  **Resorption** | **Periosteal**  **Bone Formation** | **Summed**  **Scores** |  |
| 1 | 4.50 | 2.17 | 3.83 | 2.17 | 1.33 | 14.00 | 126.67 |
| 2 | 2.83 | 1.00 | 1.67 | 1.00 | 0.67 | 7.17 | 53.33 |
| 3 | 2.67 | 0.75 | 2.00 | 0.83 | 0.50 | 6.75 | 40.00 |
| 4 | 3.00 | 0.67 | 2.00 | 0.67 | 0.33 | 6.67 | 26.67 |
| 5 | 2.50 | 0.75 | 2.00 | 0.75 | 0.33 | 6.33 | 20.00 |
| 6 | 1.75 | 1.00 | 1.83 | 1.00 | 0.67 | 6.25 | 53.33 |
| 7 | 2.00 | 0.33 | 0.92 | 0.33 | 0.17 | 3.75 | 6.67 |
| 8 | 3.08 | 1.08 | 2.83 | 1.08 | 0.50 | 8.58 | 33.33 |
| 9 | 3.17 | 1.17 | 3.00 | 1.17 | 0.67 | 9.17 | 66.67 |
| 10 | 1.00 | 0.33 | 0.92 | 0.33 | 0.33 | 2.92 | 33.33 |
| Mean | 2.65 | 0.93 | 2.10 | 0.93 | 0.55 | 7.16 | 46.00 |
| SE | 0.30 | 0.17 | 0.29 | 0.16 | 0.10 | 0.97 | 10.54 |
| ANOVA Results (post-test to grp 2) | 0.142 | 0.040 | 0.176 | 0.042 | 0.034 | 0.046 | 0.017 |
| % inhibition to Vehicle | 29% | 46% | 37% | 46% | 51% | 38% | 53% |
| ANOVA Results (post-test to grp 3) | 0.236 | 0.048 | 0.173 | 0.051 | 0.011 | 0.080 | 0.042 |
| % inhibition to MTX | 25% | 42% | 35% | 41% | 50% | 35% | 50% |
| **Group 6**  **MT-SYK-03 (103 mg/kg) + MTX (1.5 mg/kg)**  **PO, BID (MTX is QD)** | **Histopathology Scores** | | | | | | **Periosteal Bone Width**  **μm (40*25x measure)** |
|  | **Inflammation** | **Pannus** | **Cartilage**  **Damage** | **Bone**  **Resorption** | **Periosteal**  **Bone Formation** | **Summed**  **Scores** |  |
| 1 | 2.75 | 1.00 | 2.00 | 1.00 | 0.67 | 7.42 | 66.67 |
| 2 | 2.17 | 1.17 | 2.00 | 1.17 | 0.67 | 7.17 | 66.67 |
| 3 | 2.08 | 0.50 | 1.67 | 0.50 | 0.50 | 5.25 | 40.00 |
| 4 | 3.33 | 1.08 | 2.42 | 1.08 | 0.83 | 8.75 | 66.67 |
| 5 | 1.00 | 0.17 | 0.33 | 0.17 | 0.17 | 1.83 | 6.67 |
| 6 | 3.17 | 0.67 | 1.92 | 0.67 | 1.00 | 7.42 | 60.00 |
| 7 | 1.75 | 0.67 | 1.17 | 0.67 | 0.67 | 4.92 | 53.33 |
| 8 | 3.50 | 1.33 | 2.83 | 1.33 | 0.67 | 9.67 | 46.67 |
| 9 | 1.33 | 0.33 | 1.25 | 0.33 | 0.33 | 3.58 | 26.67 |
| 10 | 3.00 | 0.92 | 2.00 | 0.92 | 0.50 | 7.33 | 40.00 |
| Mean | 2.41 | 0.78 | 1.76 | 0.78 | 0.60 | 6.33 | 47.33 |
| SE | 0.28 | 0.12 | 0.22 | 0.12 | 0.08 | 0.76 | 6.24 |
| ANOVA Results (post-test to grp 2) | 0.055 | 0.011 | 0.038 | 0.011 | 0.096 | 0.024 | 0.017 |
| % inhibition to Vehicle | 35% | 55% | 47% | 55% | 47% | 45% | 52% |
| ANOVA Results (post-test to grp 3) | 0.098 | 0.013 | 0.038 | 0.013 | 0.036 | 0.043 | 0.042 |
| % inhibition to MTX | 32% | 51% | 45% | 51% | 45% | 43% | 49% |

**Table S18.** Individual Animal Summary of Paw Score (Therapeutic/Enrollment Paws)

| **Treatmeny group** | **Summed Therapeutic (Enrollment) Paw Score** | | | | | | | | | | | **Total AUC** |
| --- | --- | --- | --- | --- | --- | --- | --- | --- | --- | --- | --- | --- |
| **Group 2**  **Vehicle Control** |  |  |  |  |  |  |  |  |  |  |  |  |
|  | **Day 1** | **Day 2** | **Day 3** | **Day 4** | **Day 5** | **Day 6** | **Day 7** | **Day 8** | **Day 9** | **Day 10** | **Day 11** | **Sum (d1-11)** |
| 1 | 4 | 6 | 7 | 8 | 10 | 10 | 10 | 10 | 10 | 10 | 10 | 88 |
| 2 | 6 | 9 | 11 | 12 | 12 | 12 | 13 | 13 | 14 | 14 | 14 | 120 |
| 3 | 2 | 3 | 4 | 4 | 4 | 4 | 4 | 4 | 4 | 4 | 4 | 38 |
| 4 | 2 | 2 | 3 | 4 | 4 | 4 | 5 | 5 | 5 | 5 | 5 | 41 |
| 5 | 2 | 3 | 4 | 4 | 5 | 5 | 5 | 5 | 5 | 5 | 5 | 45 |
| 6 | 4 | 6 | 8 | 8 | 9 | 9 | 9 | 9 | 9 | 10 | 10 | 84 |
| 7 | 2 | 3 | 4 | 4 | 4 | 4 | 4 | 4 | 4 | 4 | 4 | 38 |
| 8 | 4 | 6 | 8 | 8 | 8 | 8 | 8 | 9 | 10 | 10 | 10 | 82 |
| 9 | 2 | 3 | 4 | 4 | 4 | 4 | 4 | 4 | 4 | 4 | 4 | 38 |
| 10 | 4 | 6 | 6 | 6 | 6 | 6 | 6 | 7 | 8 | 8 | 8 | 65 |
| Mean | 3.20 | 4.70 | 5.90 | 6.20 | 6.60 | 6.60 | 6.80 | 7.00 | 7.30 | 7.40 | 7.40 | 63.80 |
| SE | 0.44 | 0.70 | 0.81 | 0.87 | 0.93 | 0.93 | 0.98 | 0.99 | 1.09 | 1.11 | 1.11 | 9.07 |
| % inhibition to Vehicle | 0% | 0% | 0% | 0% | 0% | 0% | 0% | 0% | 0% | 0% | 0% | 0% |
| **Group 3**  **MTX (1.5 mg/kg) PO, QD** | **Summed Therapeutic (Enrollment) Paw Score** | | | | | | | | | | | **Total AUC** |
|  | **Day 1** | **Day 2** | **Day 3** | **Day 4** | **Day 5** | **Day 6** | **Day 7** | **Day 8** | **Day 9** | **Day 10** | **Day 11** | **Sum (d1-11)** |
| 1 | 2 | 3 | 4 | 4 | 4 | 4 | 5 | 5 | 5 | 5 | 5 | 43 |
| 2 | 2 | 2 | 3 | 4 | 4 | 4 | 4 | 4 | 4 | 4 | 4 | 36 |
| 3 | 2 | 3 | 3 | 3 | 4 | 4 | 4 | 4 | 4 | 4 | 4 | 36 |
| 4 | 4 | 5 | 5 | 7 | 7 | 7 | 7 | 7 | 7 | 7 | 7 | 65 |
| 5 | 4 | 6 | 7 | 8 | 8 | 8 | 8 | 8 | 8 | 8 | 8 | 75 |
| 6 | 6 | 8 | 9 | 12 | 15 | 15 | 15 | 15 | 15 | 15 | 15 | 130 |
| 7 | 2 | 2 | 3 | 4 | 4 | 4 | 4 | 4 | 4 | 4 | 4 | 36 |
| 8 | 4 | 5 | 7 | 9 | 9 | 9 | 9 | 9 | 9 | 9 | 9 | 82 |
| 9 | 2 | 3 | 4 | 4 | 4 | 4 | 4 | 4 | 4 | 4 | 4 | 38 |
| 10 | 4 | 6 | 7 | 8 | 8 | 8 | 8 | 8 | 9 | 9 | 9 | 78 |
| Mean | 3.20 | 4.30 | 5.20 | 6.30 | 6.70 | 6.70 | 6.80 | 6.80 | 6.90 | 6.90 | 6.90 | 61.65 |
| SE | 0.44 | 0.63 | 0.68 | 0.93 | 1.13 | 1.13 | 1.10 | 1.10 | 1.12 | 1.12 | 1.12 | 9.62 |
| ANOVA Results (post-test to grp 2) | >0.9999 | >0.9999 | >0.9999 | >0.9999 | >0.9999 | >0.9999 | >0.9999 | >0.9999 | >0.9999 | >0.9999 | >0.9999 | >0.9999 |
| % inhibition to Vehicle | 0% | 9% | 12% | −2% | −2% | −2% | 0% | 3% | 5% | 7% | 7% | 3% |

**Table S18.** Individual Animal Summary of Paw Score (Therapeutic/Enrollment Paws) (continued)

| **Group 4**  **MT-SYK-03 (103 mg/kg) PO, BID** | **Average Paw Score Per Arthritis Day** | | | | | | | | | | | **Total AUC** |
| --- | --- | --- | --- | --- | --- | --- | --- | --- | --- | --- | --- | --- |
|  | **Day 1** | **Day 2** | **Day 3** | **Day 4** | **Day 5** | **Day 6** | **Day 7** | **Day 8** | **Day 9** | **Day 10** | **Day 11** | **Sum (d1-11)** |
| 1 | 2 | 3 | 4 | 4 | 4 | 4 | 4 | 5 | 5 | 5 | 5 | 42 |
| 2 | 6 | 6 | 9 | 10 | 11 | 11 | 11 | 11 | 12 | 12 | 12 | 102 |
| 3 | 2 | 2 | 3 | 3 | 3 | 3 | 4 | 4 | 4 | 4 | 4 | 33 |
| 4 | 4 | 6 | 8 | 8 | 9 | 9 | 9 | 9 | 9 | 9 | 9 | 83 |
| 5 | 2 | 3 | 4 | 4 | 4 | 4 | 4 | 4 | 4 | 4 | 4 | 38 |
| 6 | 2 | 2 | 3 | 4 | 4 | 4 | 4 | 4 | 4 | 4 | 4 | 36 |
| 7 | 4 | 4 | 6 | 7 | 8 | 8 | 9 | 10 | 10 | 10 | 10 | 79 |
| 8 | 4 | 6 | 8 | 8 | 8 | 8 | 9 | 9 | 9 | 9 | 9 | 81 |
| 9 | 4 | 4 | 6 | 6 | 7 | 8 | 8 | 9 | 9 | 9 | 9 | 73 |
| 10 | 2 | 2 | 3 | 4 | 4 | 4 | 4 | 3 | 3 | 3 | 3 | 33 |
| Mean | 3.20 | 3.80 | 5.40 | 5.80 | 6.20 | 6.30 | 6.60 | 6.80 | 6.90 | 6.90 | 6.90 | 59.75 |
| SE | 0.44 | 0.53 | 0.73 | 0.74 | 0.87 | 0.88 | 0.90 | 0.96 | 1.02 | 1.02 | 1.02 | 8.23 |
| ANOVA Results (post-test to grp 2) | >0.9999 | >0.9999 | >0.9999 | >0.9999 | >0.9999 | >0.9999 | >0.9999 | >0.9999 | >0.9999 | >0.9999 | >0.9999 | >0.9999 |
| % inhibition to Vehicle | 0% | 19% | 8% | 6% | 6% | 5% | 3% | 3% | 5% | 7% | 7% | 6% |
| **Group 5**  **MT-SYK-03 + MTX (52 + 1.5 mg/kg)**  **PO, BID (MTX is QD)** | **Average Paw Score Per Arthritis Day** | | | | | | | | | | | **Total AUC** |
|  | **Day 1** | **Day 2** | **Day 3** | **Day 4** | **Day 5** | **Day 6** | **Day 7** | **Day 8** | **Day 9** | **Day 10** | **Day 11** | **Sum (d1-11)** |
| 1 | 6 | 8 | 11 | 13 | 14 | 14 | 14 | 14 | 15 | 15 | 15 | 129 |
| 2 | 2 | 2 | 3 | 4 | 4 | 4 | 4 | 4 | 4 | 4 | 4 | 36 |
| 3 | 4 | 4 | 4 | 5 | 5 | 5 | 6 | 6 | 6 | 6 | 6 | 52 |
| 4 | 4 | 6 | 7 | 8 | 8 | 8 | 8 | 8 | 8 | 8 | 8 | 75 |
| 5 | 2 | 2 | 2 | 3 | 3 | 3 | 3 | 3 | 3 | 3 | 3 | 28 |
| 6 | 2 | 3 | 4 | 4 | 4 | 4 | 4 | 4 | 4 | 4 | 4 | 38 |
| 7 | 2 | 3 | 3 | 3 | 3 | 3 | 3 | 3 | 2 | 2 | 2 | 27 |
| 8 | 4 | 5 | 6 | 8 | 8 | 8 | 8 | 8 | 8 | 8 | 8 | 73 |
| 9 | 4 | 6 | 6 | 8 | 8 | 8 | 8 | 9 | 9 | 9 | 9 | 78 |
| 10 | 2 | 3 | 4 | 4 | 4 | 4 | 4 | 5 | 5 | 5 | 5 | 42 |
| Mean | 3.20 | 4.20 | 5.00 | 6.00 | 6.10 | 6.10 | 6.20 | 6.40 | 6.40 | 6.40 | 6.40 | 57.60 |
| SE | 0.44 | 0.63 | 0.83 | 1.01 | 1.09 | 1.09 | 1.08 | 1.09 | 1.20 | 1.20 | 1.20 | 9.97 |
| ANOVA Results (post-test to grp 2) | >0.9999 | >0.9999 | >0.9999 | >0.9999 | >0.9999 | >0.9999 | >0.9999 | >0.9999 | >0.9999 | >0.9999 | >0.9999 | >0.9999 |
| % inhibition to Vehicle | 0% | 11% | 15% | 3% | 8% | 8% | 9% | 9% | 12% | 14% | 14% | 10% |
| ANOVA Results (post-test to grp 3) | >0.9999 | >0.9999 | >0.9999 | >0.9999 | >0.9999 | >0.9999 | >0.9999 | >0.9999 | >0.9999 | >0.9999 | >0.9999 | >0.9999 |
| % inhibition to MTX | 0% | 2% | 4% | 5% | 9% | 9% | 9% | 6% | 7% | 7% | 7% | 7% |

**Table S18.** Individual Animal Summary of Paw Score (Therapeutic/Enrollment Paws) (continued)

| **Group 6**  **MT-SYK-03 + MTX (103 + 1.5 mg/kg)**  **PO, BID (MTX is QD)** | **Average Paw Score Per Arthritis Day** | | | | | | | | | | | **Total AUC** |
| --- | --- | --- | --- | --- | --- | --- | --- | --- | --- | --- | --- | --- |
|  | **Day 1** | **Day 2** | **Day 3** | **Day 4** | **Day 5** | **Day 6** | **Day 7** | **Day 8** | **Day 9** | **Day 10** | **Day 11** | **Sum (d1-11)** |
| 1 | 6 | 8 | 9 | 9 | 9 | 9 | 10 | 10 | 10 | 10 | 10 | 92 |
| 2 | 2 | 2 | 3 | 4 | 4 | 5 | 5 | 5 | 5 | 5 | 5 | 42 |
| 3 | 2 | 2 | 3 | 4 | 4 | 4 | 4 | 4 | 4 | 4 | 4 | 36 |
| 4 | 2 | 2 | 3 | 3 | 4 | 4 | 4 | 4 | 4 | 4 | 4 | 35 |
| 5 | 2 | 2 | 2 | 2 | 2 | 2 | 2 | 2 | 1 | 1 | 1 | 18 |
| 6 | 4 | 5 | 6 | 8 | 8 | 8 | 8 | 8 | 8 | 8 | 8 | 73 |
| 7 | 4 | 4 | 6 | 6 | 6 | 6 | 6 | 6 | 6 | 5 | 5 | 56 |
| 8 | 4 | 6 | 6 | 7 | 7 | 8 | 8 | 8 | 8 | 8 | 8 | 72 |
| 9 | 2 | 2 | 3 | 4 | 4 | 4 | 4 | 4 | 4 | 4 | 4 | 36 |
| 10 | 4 | 6 | 6 | 6 | 6 | 6 | 6 | 5 | 4 | 4 | 4 | 53 |
| Mean | 3.20 | 3.90 | 4.70 | 5.30 | 5.40 | 5.60 | 5.70 | 5.60 | 5.40 | 5.30 | 5.30 | 51.15 |
| SE | 0.44 | 0.71 | 0.70 | 0.72 | 0.69 | 0.70 | 0.76 | 0.76 | 0.83 | 0.83 | 0.83 | 7.11 |
| ANOVA Results (post-test to grp 2) | >0.9999 | >0.9999 | >0.9999 | >0.9999 | >0.9999 | >0.9999 | >0.9999 | >0.9999 | >0.9999 | >0.9999 | >0.9999 | >0.9999 |
| % inhibition to Vehicle | 0% | 17% | 20% | 15% | 18% | 15% | 16% | 20% | 26% | 28% | 28% | 20% |
| ANOVA Results (post-test to grp 3) | >0.9999 | >0.9999 | >0.9999 | >0.9999 | >0.9999 | >0.9999 | >0.9999 | >0.9999 | >0.9999 | >0.9999 | >0.9999 | >0.9999 |
| % inhibition to MTX | 0% | 9% | 10% | 16% | 19% | 16% | 16% | 18% | 22% | 23% | 23% | 17% |

**Table S19.** Individual Animal Summary of Paw Score (Prophylactic/Non-Enrollment Paws)

| **Treatmeny group** |  |  |  |  |  |  |  |  |  |  |  |  |
| --- | --- | --- | --- | --- | --- | --- | --- | --- | --- | --- | --- | --- |
| **Group 1**  **Naïve** | **Summed Prophylactic (Non-Enrollment) Paw Score** | | | | | | | | | | | **Total AUC** |
|  | **Day 1** | **Day 2** | **Day 3** | **Day 4** | **Day 5** | **Day 6** | **Day 7** | **Day 8** | **Day 9** | **Day 10** | **Day 11** | **Sum (d1-11)** |
| 1 | 0 | 0 | 0 | 0 | 0 | 0 | 0 | 0 | 0 | 0 | 0 | 0.0 |
| 2 | 0 | 0 | 0 | 0 | 0 | 0 | 0 | 0 | 0 | 0 | 0 | 0.0 |
| 3 | 0 | 0 | 0 | 0 | 0 | 0 | 0 | 0 | 0 | 0 | 0 | 0.0 |
| 4 | 0 | 0 | 0 | 0 | 0 | 0 | 0 | 0 | 0 | 0 | 0 | 0.0 |
| Mean | 0.00 | 0.00 | 0.00 | 0.00 | 0.00 | 0.00 | 0.00 | 0.00 | 0.00 | 0.00 | 0.00 | 0.00 |
| SE | 0.00 | 0.00 | 0.00 | 0.00 | 0.00 | 0.00 | 0.00 | 0.00 | 0.00 | 0.00 | 0.00 | 0.00 |
| *t*-test to Vehicle |  | 0.028 | 0.002 | 0.002 | 0.001 | 0.001 | 0.002 | 0.002 | 0.002 | 0.002 | 0.002 | 0.002 |
| % inhibition to Vehicle |  | 100% | 100% | 100% | 100% | 100% | 100% | 100% | 100% | 100% | 100% | 100% |
| **Group 2**  **Vehicle Control** | **Summed Prophylactic (Non-Enrollment) Paw Score** | | | | | | | | | | | **Total AUC** |
|  | **Day 1** | **Day 2** | **Day 3** | **Day 4** | **Day 5** | **Day 6** | **Day 7** | **Day 8** | **Day 9** | **Day 10** | **Day 11** | **Sum (d1-11)** |
| 1 | 0 | 4 | 6 | 8 | 9 | 10 | 10 | 10 | 10 | 10 | 10 | 82.0 |
| 2 | 0 | 2 | 3 | 3 | 4 | 4 | 4 | 4 | 4 | 4 | 4 | 34.0 |
| 3 | 0 | 2 | 2 | 4 | 5 | 5 | 5 | 6 | 6 | 7 | 7 | 45.5 |
| 4 | 0 | 2 | 3 | 4 | 4 | 5 | 5 | 6 | 6 | 7 | 7 | 45.5 |
| 5 | 0 | 4 | 8 | 10 | 12 | 12 | 13 | 13 | 14 | 15 | 15 | 108.5 |
| 6 | 0 | 0 | 4 | 6 | 8 | 8 | 9 | 9 | 10 | 10 | 10 | 69.0 |
| 7 | 0 | 2 | 4 | 5 | 6 | 6 | 6 | 6 | 6 | 6 | 6 | 50.0 |
| 8 | 0 | 4 | 6 | 8 | 8 | 8 | 8 | 8 | 8 | 8 | 8 | 70.0 |
| 9 | 0 | 6 | 9 | 12 | 12 | 13 | 13 | 13 | 14 | 14 | 14 | 113.0 |
| 10 | 0 | 0 | 2 | 4 | 5 | 5 | 5 | 6 | 7 | 7 | 7 | 44.5 |
| Mean | 0.00 | 2.60 | 4.70 | 6.40 | 7.30 | 7.60 | 7.80 | 8.10 | 8.50 | 8.80 | 8.80 | 66.20 |
| SE | 0.00 | 0.60 | 0.78 | 0.95 | 0.96 | 1.00 | 1.06 | 0.98 | 1.09 | 1.10 | 1.10 | 8.74 |
| % inhibition to Vehicle |  | 0% | 0% | 0% | 0% | 0% | 0% | 0% | 0% | 0% | 0% | 0% |

**Table S19**. Individual Animal Summary of Paw Score (Prophylactic/Non-Enrollment Paws) (continued)

| **Group 3**  **MTX (1.5 mg/kg) PO, QD** | **Summed Prophylactic (Non-Enrollment) Paw Score** | | | | | | | | | | | **Total AUC** |
| --- | --- | --- | --- | --- | --- | --- | --- | --- | --- | --- | --- | --- |
|  | **Day 1** | **Day 2** | **Day 3** | **Day 4** | **Day 5** | **Day 6** | **Day 7** | **Day 8** | **Day 9** | **Day 10** | **Day 11** | **Sum (d1-11)** |
| 1 | 0 | 1 | 4 | 6 | 9 | 9 | 9 | 10 | 11 | 14 | 14 | 80.0 |
| 2 | 0 | 2 | 7 | 10 | 12 | 12 | 12 | 12 | 13 | 14 | 14 | 101.0 |
| 3 | 0 | 5 | 8 | 10 | 10 | 10 | 10 | 10 | 10 | 10 | 10 | 88.0 |
| 4 | 0 | 2 | 3 | 4 | 4 | 4 | 4 | 4 | 4 | 4 | 4 | 35.0 |
| 5 | 0 | 4 | 5 | 6 | 6 | 6 | 6 | 6 | 7 | 8 | 8 | 58.0 |
| 6 | 0 | 0 | 2 | 4 | 4 | 4 | 4 | 4 | 5 | 5 | 5 | 34.5 |
| 7 | 0 | 0 | 0 | 3 | 5 | 6 | 6 | 6 | 6 | 6 | 6 | 41.0 |
| 8 | 0 | 0 | 0 | 3 | 4 | 4 | 5 | 5 | 5 | 5 | 5 | 33.5 |
| 9 | 0 | 2 | 3 | 4 | 4 | 4 | 4 | 4 | 4 | 4 | 4 | 35.0 |
| 10 | 0 | 0 | 1 | 1 | 1 | 1 | 1 | 2 | 3 | 4 | 4 | 16.0 |
| Mean | 0.00 | 1.60 | 3.30 | 5.10 | 5.90 | 6.00 | 6.10 | 6.30 | 6.80 | 7.40 | 7.40 | 52.20 |
| SE | 0.00 | 0.56 | 0.87 | 0.94 | 1.07 | 1.06 | 1.05 | 1.03 | 1.07 | 1.26 | 1.26 | 8.92 |
| ANOVA Results (post-test to grp 2) |  | 0.731 | 0.835 | >0.9999 | >0.9999 | >0.9999 | >0.9999 | 0.925 | >0.9999 | >0.9999 | >0.9999 | >0.9999 |
| % inhibition to Vehicle |  | 38% | 30% | 20% | 19% | 21% | 22% | 22% | 20% | 16% | 16% | 21% |
| **Group 4**  **MT-SYK-03 (103 mg/kg) PO, BID** | **Summed Prophylactic (Non-Enrollment) Paw Score** | | | | | | | | | | | **Total AUC** |
|  | **Day 1** | **Day 2** | **Day 3** | **Day 4** | **Day 5** | **Day 6** | **Day 7** | **Day 8** | **Day 9** | **Day 10** | **Day 11** | **Sum (d1-11)** |
| 1 | 0 | 2 | 5 | 7 | 8 | 8 | 8 | 8 | 8 | 9 | 9 | 67.5 |
| 2 | 0 | 2 | 3 | 3 | 3 | 3 | 3 | 3 | 3 | 3 | 3 | 27.5 |
| 3 | 0 | 2 | 2 | 5 | 8 | 9 | 9 | 9 | 10 | 11 | 12 | 71.0 |
| 4 | 0 | 4 | 5 | 6 | 6 | 6 | 7 | 7 | 8 | 8 | 8 | 61.0 |
| 5 | 0 | 1 | 1 | 1 | 1 | 1 | 1 | 1 | 2 | 2 | 2 | 12.0 |
| 6 | 0 | 3 | 5 | 7 | 10 | 10 | 10 | 10 | 10 | 10 | 10 | 80.0 |
| 7 | 0 | 4 | 6 | 7 | 8 | 8 | 8 | 8 | 8 | 9 | 9 | 70.5 |
| 8 | 0 | 2 | 3 | 6 | 6 | 6 | 6 | 6 | 6 | 7 | 7 | 51.5 |
| 9 | 0 | 2 | 4 | 6 | 7 | 8 | 8 | 8 | 8 | 8 | 8 | 63.0 |
| 10 | 0 | 0 | 2 | 4 | 4 | 5 | 5 | 5 | 5 | 5 | 5 | 37.5 |
| Mean | 0.00 | 2.20 | 3.60 | 5.20 | 6.10 | 6.40 | 6.50 | 6.50 | 6.80 | 7.20 | 7.30 | 54.15 |
| SE | 0.00 | 0.39 | 0.52 | 0.63 | 0.86 | 0.88 | 0.89 | 0.89 | 0.87 | 0.94 | 0.99 | 6.91 |
| ANOVA Results (post-test to grp 2) |  | >0.9999 | >0.9999 | >0.9999 | >0.9999 | >0.9999 | >0.9999 | >0.9999 | >0.9999 | >0.9999 | >0.9999 | >0.9999 |
| % inhibition to Vehicle |  | 15% | 23% | 19% | 16% | 16% | 17% | 20% | 20% | 18% | 17% | 18% |

**Table S19**. Individual Animal Summary of Paw Score (Prophylactic/Non-Enrollment Paws) (continued)

| **Group 5**  **MT-SYK-03 + MTX (52 + 1.5 mg/kg)**  **PO, BID (MTX is QD)** | **Summed Prophylactic (Non-Enrollment) Paw Score** | | | | | | | | | | | **Total AUC** |
| --- | --- | --- | --- | --- | --- | --- | --- | --- | --- | --- | --- | --- |
|  | **Day 1** | **Day 2** | **Day 3** | **Day 4** | **Day 5** | **Day 6** | **Day 7** | **Day 8** | **Day 9** | **Day 10** | **Day 11** | **Sum (d1-11)** |
| 1 | 0 | 1 | 2 | 2 | 2 | 2 | 2 | 2 | 2 | 2 | 2 | 18.0 |
| 2 | 0 | 3 | 5 | 9 | 11 | 11 | 12 | 12 | 12 | 12 | 12 | 93.0 |
| 3 | 0 | 2 | 4 | 5 | 5 | 6 | 6 | 6 | 6 | 6 | 6 | 49.0 |
| 4 | 0 | 0 | 0 | 0 | 0 | 0 | 0 | 0 | 0 | 0 | 0 | 0.0 |
| 5 | 0 | 3 | 5 | 6 | 8 | 8 | 8 | 8 | 10 | 11 | 11 | 72.5 |
| 6 | 0 | 1 | 2 | 3 | 4 | 4 | 4 | 4 | 4 | 4 | 4 | 32.0 |
| 7 | 0 | 0 | 0 | 2 | 4 | 4 | 4 | 4 | 4 | 3 | 3 | 26.5 |
| 8 | 0 | 0 | 2 | 4 | 3 | 3 | 3 | 3 | 2 | 2 | 2 | 23.0 |
| 9 | 0 | 0 | 4 | 6 | 6 | 7 | 7 | 7 | 7 | 7 | 7 | 54.5 |
| 10 | 0 | 0 | 0 | 0 | 0 | 0 | 0 | 0 | 0 | 0 | 0 | 0.0 |
| Mean | 0.00 | 1.00 | 2.40 | 3.70 | 4.30 | 4.50 | 4.60 | 4.60 | 4.70 | 4.70 | 4.70 | 36.85 |
| SE | 0.00 | 0.39 | 0.64 | 0.91 | 1.09 | 1.12 | 1.19 | 1.19 | 1.28 | 1.34 | 1.34 | 9.59 |
| ANOVA Results (post-test to grp 2) |  | 0.155 | 0.192 | 0.194 | 0.143 | 0.165 | 0.143 | 0.095 | 0.092 | 0.078 | 0.075 | 0.096 |
| % inhibition to Vehicle |  | 62% | 49% | 42% | 41% | 41% | 41% | 43% | 45% | 47% | 47% | 44% |
| ANOVA Results (post-test to grp 3) |  | 0.155 | 0.192 | 0.194 | 0.143 | 0.165 | 0.143 | 0.095 | 0.092 | 0.078 | 0.075 | 0.096 |
| % inhibition to MTX |  | 38% | 27% | 27% | 27% | 25% | 25% | 27% | 31% | 36% | 36% | 29% |
| **Group 6**  **MT-SYK-03 + MTX (103 + 1.5 mg/kg)**  **PO, BID (MTX is QD)** | **Summed Prophylactic (Non-Enrollment) Paw Score** | | | | | | | | | | | **Total AUC** |
|  | **Day 1** | **Day 2** | **Day 3** | **Day 4** | **Day 5** | **Day 6** | **Day 7** | **Day 8** | **Day 9** | **Day 10** | **Day 11** | **Sum (d1-11)** |
| 1 | 0 | 0 | 0 | 0 | 0 | 0 | 0 | 0 | 0 | 0 | 0 | 0.0 |
| 2 | 0 | 2 | 3 | 3 | 4 | 4 | 4 | 4 | 5 | 5 | 5 | 36.5 |
| 3 | 0 | 2 | 3 | 3 | 4 | 4 | 4 | 4 | 4 | 4 | 4 | 34.0 |
| 4 | 0 | 4 | 5 | 6 | 6 | 6 | 6 | 7 | 7 | 8 | 8 | 59.0 |
| 5 | 0 | 0 | 0 | 2 | 3 | 3 | 3 | 3 | 3 | 3 | 3 | 21.5 |
| 6 | 0 | 1 | 4 | 6 | 8 | 8 | 8 | 8 | 8 | 8 | 8 | 63.0 |
| 7 | 0 | 0 | 0 | 1 | 1 | 1 | 1 | 1 | 1 | 1 | 1 | 7.5 |
| 8 | 0 | 2 | 5 | 7 | 8 | 8 | 8 | 8 | 8 | 8 | 8 | 66.0 |
| 9 | 0 | 0 | 0 | 0 | 0 | 0 | 0 | 0 | 0 | 0 | 0 | 0.0 |
| 10 | 0 | 0 | 2 | 4 | 4 | 4 | 4 | 4 | 4 | 4 | 4 | 32.0 |
| Mean | 0.00 | 1.10 | 2.20 | 3.20 | 3.80 | 3.80 | 3.80 | 3.90 | 4.00 | 4.10 | 4.10 | 31.95 |
| SE | 0.00 | 0.43 | 0.66 | 0.80 | 0.93 | 0.93 | 0.93 | 0.96 | 0.97 | 1.00 | 1.00 | 7.91 |
| ANOVA Results (post-test to grp 2) |  | 0.195 | 0.132 | 0.084 | 0.068 | 0.053 | 0.044 | 0.035 | 0.037 | 0.043 | 0.043 | 0.040 |
| % inhibition to Vehicle |  | 58% | 53% | 50% | 48% | 50% | 51% | 52% | 53% | 53% | 53% | 52% |
| ANOVA Results (post-test to grp 3) |  | 0.195 | 0.132 | 0.084 | 0.068 | 0.053 | 0.044 | 0.035 | 0.037 | 0.043 | 0.043 | 0.040 |
| % inhibition to MTX |  | 31% | 33% | 37% | 36% | 37% | 38% | 38% | 41% | 45% | 45% | 39% |

**Table S20.** Histopathology Report for Ankles after CIA model induction in rats at study day 16.

| **Treatment Group** | | **Histopathology Scores (from 0=normal to 5=severe)** | | | | | **Summed**  **Histopathology**  **Scores** | **Periosteal**  **Naïve Bone Width** | |
| --- | --- | --- | --- | --- | --- | --- | --- | --- | --- |
| **Group 1**  **Naïve** | | **Inflammation** | **Pannus** | **Cartilage Damage** | **Bone Resorption** | **Periosteal Bone Formation** |  | **Actual μm** | **16x Measure** |
| **1** | **R** | 0 | 0 | 0 | 0 | 0 | 0 | 0 |  |
|  | **L** | 0 | 0 | 0 | 0 | 0 | 0 | 0 |  |
| **2** | **R** | 0 | 0 | 0 | 0 | 0 | 0 | 0 |  |
|  | **L** | 0 | 0 | 0 | 0 | 0 | 0 | 0 |  |
| **3** | **R** | 0 | 0 | 0 | 0 | 0 | 0 | 0 |  |
|  | **L** | 0 | 0 | 0 | 0 | 0 | 0 | 0 |  |
| **4** | **R** | 0 | 0 | 0 | 0 | 0 | 0 | 0 |  |
|  | **L** | 0 | 0 | 0 | 0 | 0 | 0 | 0 |  |
| Mean | | 0.00 | 0.00 | 0.00 | 0.00 | 0.00 | 0.00 | 0.00 |  |
| SE | | 0.00 | 0.00 | 0.00 | 0.00 | 0.00 | 0.00 | 0.00 |  |
| ANOVA to Vehicle | | p < 0.05 | p < 0.05 | p < 0.05 | p < 0.05 | p < 0.05 | p < 0.05 | p < 0.05 |  |
| % inhibition | | 100% | 100% | 100% | 100% | 100% | 100% | 100% |  |
| **Group 2**  **Test Art. Veh (PO, BID)**  **MTX Veh (PO, QD)** | | **Histopathology Scores (from 0=normal to 5=severe)** | | | | | **Summed**  **Histopathology**  **Scores** | **Periosteal**  **Naïve Bone Width** | |
|  |  | **Inflammation** | **Pannus** | **Cartilage Damage** | **Bone Resorption** | **Periosteal Bone Formation** |  | **Actual μm** | **16x Measure** |
| **1** | **R** | 2 | 0.5 | 1 | 0.5 | 0 | 4 | 0 | 0 |
|  | **L** | 5 | 2 | 3 | 2 | 1 | 13 | 189 | 3 |
| **2** | **R** | 5 | 1 | 2 | 1 | 0.5 | 9.5 | 63 | 1 |
|  | **L** | 5 | 2 | 3 | 2 | 2 | 14 | 315 | 5 |
| **3** | **R** | 1 | 0 | 0.5 | 0 | 0 | 1.5 | 0 | 0 |
|  | **L** | 5 | 2 | 3 | 2 | 1 | 13 | 189 | 3 |
| **4** | **R** | 5 | 1 | 2 | 1 | 1 | 10 | 189 | 3 |
|  | **L** | 5 | 2 | 3 | 2 | 2 | 14 | 315 | 5 |
| **5** | **R** | 5 | 2 | 3 | 2 | 1 | 13 | 189 | 3 |
|  | **L** | 4 | 1 | 2 | 1 | 1 | 9 | 189 | 3 |
| **6** | **R** | 5 | 1 | 2 | 1 | 2 | 11 | 315 | 5 |
|  | **L** | 5 | 2 | 3 | 2 | 2 | 14 | 378 | 6 |
| **7** | **R** | 5 | 1 | 2 | 1 | 2 | 11 | 315 | 5 |
|  | **L** | 5 | 2 | 3 | 2 | 1 | 13 | 189 | 3 |
| **8** | **R** | 5 | 2 | 3 | 2 | 2 | 14 | 441 | 7 |
|  | **L** | 5 | 2 | 3 | 2 | 2 | 14 | 315 | 5 |
| Mean | | 4.50 | 1.47 | 2.41 | 1.47 | 1.28 | 11.13 | 224.44 |  |
| SE | | 0.30 | 0.17 | 0.20 | 0.17 | 0.18 | 0.93 | 32.00 |  |
| % inhibition | | 0% | 0% | 0% | 0% | 0% | 0% | 0% |  |

**Table S20**. Histopathology Report for Ankles after CIA model induction in rats at study day 16 (continued).

| **Treatment Group** | | **Histopathology Scores (from 0=normal to 5=severe)** | | | | | **Summed**  **Histopathology**  **Scores** | **Periosteal**  **Naïve Bone Width** | |
| --- | --- | --- | --- | --- | --- | --- | --- | --- | --- |
| **Group 3**  **MTX (0.075 mg/kg), (PO, QD)**  **Test Article Vehicle (PO, BID)** | | **Inflammation** | **Pannus** | **Cartilage Damage** | **Bone Resorption** | **Periosteal Bone Formation** |  | **Actual μm** | **16x Measure** |
| **1** | **R** | 5 | 1 | 2 | 1 | 0.5 | 9.5 | 63 | 1 |
|  | **L** | 5 | 2 | 3 | 2 | 1 | 13 | 189 | 3 |
| **2** | **R** | 5 | 1 | 2 | 1 | 0.5 | 9.5 | 126 | 2 |
|  | **L** | 2 | 0.5 | 0.5 | 0.5 | 0.5 | 4 | 63 | 1 |
| **3** | **R** | 5 | 2 | 3 | 2 | 1 | 13 | 189 | 3 |
|  | **L** | 5 | 2 | 3 | 2 | 1 | 13 | 252 | 4 |
| **4** | **R** | 5 | 2 | 3 | 2 | 1 | 13 | 189 | 3 |
|  | **L** | 5 | 2 | 3 | 2 | 1 | 13 | 252 | 4 |
| **5** | **R** | 1 | 0 | 0.5 | 0 | 0 | 1.5 | 0 | 0 |
|  | **L** | 5 | 1 | 2 | 1 | 0.5 | 9.5 | 126 | 2 |
| **6** | **R** | 4 | 1 | 2 | 1 | 0.5 | 8.5 | 63 | 1 |
|  | **L** | 3 | 0.5 | 2 | 0.5 | 0.5 | 6.5 | 126 | 2 |
| **7** | **R** | 1 | 0 | 0.5 | 0 | 0 | 1.5 | 0 | 0 |
|  | **L** | 5 | 2 | 2 | 2 | 1 | 12 | 189 | 3 |
| **8** | **R** | 1 | 0 | 0.5 | 0 | 0 | 1.5 | 0 | 0 |
|  | **L** | 5 | 2 | 3 | 2 | 1 | 13 | 189 | 3 |
| Mean | | 3.88 | 1.19 | 2.00 | 1.19 | 0.63 | 8.88 | 126.00 |  |
| SE | | 0.42 | 0.20 | 0.25 | 0.20 | 0.10 | 1.13 | 21.52 |  |
| ANOVA to Vehicle | | NS | NS | NS | NS | NS | NS | p < 0.05 |  |
| % inhibition | | 14% | 19% | 17% | 19% | 51% | 20% | 44% |  |

**Table S20**. Histopathology Report for Ankles after CIA model induction in rats at study day 16 (continued).

| **Treatment Group** | | **Histopathology Scores (from 0=normal to 5=severe)** | | | | | **Summed**  **Histopathology**  **Scores** | **Periosteal**  **Naïve Bone Width** | |
| --- | --- | --- | --- | --- | --- | --- | --- | --- | --- |
| **Group 4**  **MT-SYK-03 (207 mg/kg)**  **(PO, BID)** | | **Inflammation** | **Pannus** | **Cartilage Damage** | **Bone Resorption** | **Periosteal Bone Formation** |  | **Actual μm** | **16x Measure** |
| **1** | **R** | 0.5 | 0 | 0.5 | 0 | 0 | 1 | 0 | 0 |
|  | **L** | 1 | 0 | 0.5 | 0 | 0 | 1.5 | 0 | 0 |
| **2** | **R** | 2 | 0.5 | 0.5 | 0.5 | 0 | 3.5 | 0 | 0 |
|  | **L** | 2 | 0.5 | 1 | 0.5 | 0 | 4 | 0 | 0 |
| **3** | **R** | 5 | 2 | 3 | 2 | 2 | 14 | 315 | 5 |
|  | **L** | 3 | 1 | 1 | 1 | 0.5 | 6.5 | 63 | 1 |
| **4** | **R** | 4 | 1 | 2 | 1 | 0.5 | 8.5 | 63 | 1 |
|  | **L** | 5 | 2 | 3 | 2 | 0.5 | 12.5 | 126 | 2 |
| **5** | **R** | 5 | 2 | 3 | 2 | 1 | 13 | 189 | 3 |
|  | **L** | 5 | 2 | 2 | 2 | 1 | 12 | 252 | 4 |
| **6** | **R** | 5 | 1 | 2 | 1 | 1 | 10 | 189 | 3 |
|  | **L** | 5 | 1 | 2 | 1 | 1 | 10 | 189 | 3 |
| **7** | **R** | 5 | 1 | 2 | 1 | 1 | 10 | 189 | 3 |
|  | **L** | 5 | 1 | 2 | 1 | 1 | 10 | 189 | 3 |
| **8** | **R** | 5 | 1 | 2 | 1 | 1 | 10 | 189 | 3 |
|  | **L** | 4 | 1 | 2 | 1 | 0.5 | 8.5 | 126 | 2 |
| Mean | | 3.84 | 1.06 | 1.78 | 1.06 | 0.69 | 8.44 | 129.94 |  |
| SE | | 0.40 | 0.16 | 0.21 | 0.16 | 0.14 | 1.01 | 24.72 |  |
| ANOVA to Vehicle | | NS | NS | NS | NS | NS | NS | p < 0.05 |  |
| % inhibition | | 15% | 28% | 26% | 28% | 46% | 24% | 42% |  |

**Table S20**. Histopathology Report for Ankles after CIA model induction in rats at study day 16 (continued).

| **Treatment Group** | | **Histopathology Scores (from 0=normal to 5=severe)** | | | | | **Summed**  **Histopathology**  **Scores** | **Periosteal**  **Naïve Bone Width** | |
| --- | --- | --- | --- | --- | --- | --- | --- | --- | --- |
| **Group 5**  **MT-SYK-03 (103 mg/kg) (PO, BID)**  **MTX Veh (PO, QD))** | | **Inflammation** | **Pannus** | **Cartilage Damage** | **Bone Resorption** | **Periosteal Bone Formation** |  | **Actual μm** | **16x Measure** |
| **1** | **R** | 4 | 1 | 2 | 1 | 0.5 | 8.5 | 63 | 1 |
|  | **L** | 3 | 1 | 1 | 1 | 0.5 | 6.5 | 63 | 1 |
| **2** | **R** | 4 | 1 | 2 | 1 | 2 | 10 | 315 | 5 |
|  | **L** | 5 | 2 | 2 | 2 | 2 | 13 | 315 | 5 |
| **3** | **R** | 5 | 2 | 3 | 2 | 2 | 14 | 315 | 5 |
|  | **L** | 5 | 2 | 3 | 2 | 1 | 13 | 252 | 4 |
| **4** | **R** | 5 | 2 | 3 | 2 | 2 | 14 | 315 | 5 |
|  | **L** | 5 | 2 | 3 | 2 | 2 | 14 | 315 | 5 |
| **5** | **R** | 2 | 0.5 | 0.5 | 0.5 | 0 | 3.5 | 0 | 0 |
|  | **L** | 5 | 2 | 2 | 2 | 1 | 12 | 189 | 3 |
| **6** | **R** | 5 | 2 | 2 | 2 | 1 | 12 | 189 | 3 |
|  | **L** | 5 | 2 | 3 | 2 | 1 | 13 | 189 | 3 |
| **7** | **R** | 5 | 2 | 3 | 2 | 2 | 14 | 315 | 5 |
|  | **L** | 5 | 1 | 2 | 1 | 1 | 10 | 189 | 3 |
| **8** | **R** | 4 | 0.5 | 1 | 0.5 | 0.5 | 6.5 | 126 | 2 |
|  | **L** | 4 | 1 | 1 | 1 | 0.5 | 7.5 | 63 | 1 |
| Mean | | 4.44 | 1.50 | 2.09 | 1.50 | 1.19 | 10.72 | 200.81 |  |
| SE | | 0.22 | 0.15 | 0.22 | 0.15 | 0.18 | 0.83 | 27.71 |  |
| ANOVA to Vehicle | | NS | NS | NS | NS | NS | NS | NS |  |
| % inhibition | | 1% | -2% | 13% | −2% | 7% | 4% | 11% |  |

**Table S20**. Histopathology Report for Ankles after CIA model induction in rats at study day 16 (continued).

| **Treatment Group** | | **Histopathology Scores (from 0=normal to 5=severe)** | | | | | **Summed**  **Histopathology**  **Scores** | **Periosteal**  **Naïve Bone Width** | |
| --- | --- | --- | --- | --- | --- | --- | --- | --- | --- |
| **Group 6**  **MT-SYK-03 (103 mg/kg) (PO, BID)**  **MTX (0.075 mg/kg) (PO, QD)** | | **Inflammation** | **Pannus** | **Cartilage Damage** | **Bone Resorption** | **Periosteal Bone Formation** |  | **Actual μm** | **16x Measure** |
| **1** | **R** | 4 | 1 | 2 | 1 | 1 | 9 | 189 | 3 |
|  | **L** | 2 | 0.5 | 0.5 | 0.5 | 0 | 3.5 | 0 | 0 |
| **2** | **R** | 1 | 0 | 0.5 | 0 | 0 | 1.5 | 0 | 0 |
|  | **L** | 0.5 | 0 | 0.5 | 0 | 0 | 1 | 0 | 0 |
| **3** | **R** | 3 | 0.5 | 0.5 | 0.5 | 0.5 | 5 | 63 | 1 |
|  | **L** | 3 | 0.5 | 0.5 | 0.5 | 0.5 | 5 | 63 | 1 |
| **4** | **R** | 3 | 0.5 | 0.5 | 0.5 | 0.5 | 5 | 63 | 1 |
|  | **L** | 0.5 | 0 | 0.5 | 0 | 0 | 1 | 0 | 0 |
| **5** | **R** | 1 | 0 | 0.5 | 0 | 0 | 1.5 | 0 | 0 |
|  | **L** | 5 | 1 | 2 | 1 | 1 | 10 | 189 | 3 |
| **6** | **R** | 3 | 0.5 | 1 | 0.5 | 0.5 | 5.5 | 63 | 1 |
|  | **L** | 3 | 0.5 | 1 | 0.5 | 0.5 | 5.5 | 63 | 1 |
| **7** | **R** | 5 | 2 | 3 | 2 | 1 | 13 | 189 | 3 |
|  | **L** | 3 | 0.5 | 1 | 0.5 | 0.5 | 5.5 | 63 | 1 |
| **8** | **R** | 1 | 0 | 0.5 | 0 | 0 | 1.5 | 0 | 0 |
|  | **L** | 4 | 1 | 2 | 1 | 1 | 9 | 189 | 3 |
| Mean | | 2.63 | 0.53 | 1.03 | 0.53 | 0.44 | 5.16 | 70.88 |  |
| SE | | 0.37 | 0.13 | 0.20 | 0.13 | 0.10 | 0.90 | 18.97 |  |
| ANOVA to Vehicle | | p < 0.05 | p < 0.05 | p < 0.05 | p < 0.05 | p < 0.05 | p < 0.05 | p < 0.05 |  |
| % inhibition | | 42% | 64% | 57% | 64% | 66% | 54% | 68% |  |

**Table S21.** Histopathology Report for Knees after CIA model induction in rats at study day 16.

| **Treatment Group** | | **Histopathology Scores (from 0=normal to 5=severe)** | | | | **Summed**  **Histopathology**  **Scores** |
| --- | --- | --- | --- | --- | --- | --- |
| **Group 1**  **Naïve** | | **Inflammation** | **Pannus** | **Cartilage Damage** | **Bone Resorption** |  |
| **1** | **R** | 0 | 0 | 0 | 0 | 0 |
|  | **L** | 0 | 0 | 0 | 0 | 0 |
| **2** | **R** | 0 | 0 | 0 | 0 | 0 |
|  | **L** | 0 | 0 | 0 | 0 | 0 |
| **3** | **R** | 0 | 0 | 0 | 0 | 0 |
|  | **L** | 0 | 0 | 0 | 0 | 0 |
| **4** | **R** | 0 | 0 | 0 | 0 | 0 |
|  | **L** | 0 | 0 | 0 | 0 | 0 |
| Mean | | 0.00 | 0.00 | 0.00 | 0.00 | 0.00 |
| SE | | 0.00 | 0.00 | 0.00 | 0.00 | 0.00 |
| ANOVA to Vehicle | | p < 0.05 | p < 0.05 | p < 0.05 | p < 0.05 | p < 0.05 |
| % inhibition | | 100% | 100% | 100% | 100% | 100% |
| **Group 2**  **Test Art. Veh (PO, BID)**  **MTX Veh (PO, QD)** | | **Histopathology Scores (from 0=normal to 5=severe)** | | | | **Summed**  **Histopathology**  **Scores** |
|  |  | **Inflammation** | **Pannus** | **Cartilage Damage** | **Bone Resorption** |  |
| **1** | **R** | 0.5 | 0 | 0.5 | 0 | 1 |
|  | **L** | 3 | 0.5 | 1 | 0.5 | 5 |
| **2** | **R** | 1 | 0.5 | 0.5 | 0 | 2 |
|  | **L** | 5 | 1 | 2 | 1 | 9 |
| **3** | **R** | 0.5 | 0 | 0 | 0 | 0.5 |
|  | **L** | 5 | 1 | 2 | 1 | 9 |
| **4** | **R** | 3 | 0.5 | 1 | 0.5 | 5 |
|  | **L** | 5 | 1 | 2 | 1 | 9 |
| **5** | **R** | 0.5 | 0 | 0.5 | 0 | 1 |
|  | **L** | 5 | 1 | 2 | 1 | 9 |
| **6** | **R** | 5 | 1 | 2 | 1 | 9 |
|  | **L** | 3 | 0.5 | 1 | 0.5 | 5 |
| **7** | **R** | 4 | 1 | 1 | 1 | 7 |
|  | **L** | 0.5 | 0 | 0.5 | 0 | 1 |
| **8** | **R** | 5 | 2 | 2 | 2 | 11 |
|  | **L** | 5 | 2 | 3 | 2 | 12 |
| Mean | | 3.19 | 0.75 | 1.31 | 0.72 | 5.97 |
| SE | | 0.49 | 0.16 | 0.21 | 0.16 | 0.98 |
| % inhibition | | 0% | 0% | 0% | 0% | 0% |

**Table S21**. Histopathology Report for Knees after CIA model induction in rats at study day 16 (continued).

| **Treatment Group** | | **Histopathology Scores (from 0=normal to 5=severe)** | | | | **Summed**  **Histopathology**  **Scores** |
| --- | --- | --- | --- | --- | --- | --- |
| **Group 3**  **MTX (0.075 mg/kg), (PO, QD)**  **Test Article Vehicle (PO, BID)** | | **Inflammation** | **Pannus** | **Cartilage Damage** | **Bone Resorption** |  |
| **1** | **R** | 4 | 0.5 | 1 | 0.5 | 6 |
|  | **L** | 3 | 1 | 1 | 0.5 | 5.5 |
| **2** | **R** | 2 | 0.5 | 0.5 | 0 | 3 |
|  | **L** | 0.5 | 0 | 0.5 | 0 | 1 |
| **3** | **R** | 3 | 1 | 1 | 0.5 | 5.5 |
|  | **L** | 3 | 1 | 1 | 0.5 | 5.5 |
| **4** | **R** | 3 | 0.5 | 1 | 0.5 | 5 |
|  | **L** | 3 | 0.5 | 0.5 | 0.5 | 4.5 |
| **5** | **R** | 0.5 | 0 | 0 | 0 | 0.5 |
|  | **L** | 0.5 | 0 | 0 | 0 | 0.5 |
| **6** | **R** | 1 | 0.5 | 0.5 | 0 | 2 |
|  | **L** | 0.5 | 0 | 0.5 | 0 | 1 |
| **7** | **R** | 0 | 0 | 0 | 0 | 0 |
|  | **L** | 3 | 0.5 | 1 | 0.5 | 5 |
| **8** | **R** | 0.5 | 0 | 0 | 0 | 0.5 |
|  | **L** | 5 | 1 | 2 | 1 | 9 |
| Mean | | 2.03 | 0.44 | 0.66 | 0.28 | 3.41 |
| SE | | 0.38 | 0.10 | 0.13 | 0.08 | 0.67 |
| ANOVA to Vehicle | | NS | NS | NS | NS | NS |
| % inhibition | | 36% | 42% | 50% | 61% | 43% |

**Table S21**. Histopathology Report for Knees after CIA model induction in rats at study day 16 (continued).

| **Treatment Group** | | **Histopathology Scores (from 0=normal to 5=severe)** | | | | **Summed**  **Histopathology**  **Scores** |
| --- | --- | --- | --- | --- | --- | --- |
| **Group 4**  **MT-SYK-03 (207 mg/kg) (PO, BID)**  **MTX Veh (PO, QD)** | | **Inflammation** | **Pannus** | **Cartilage Damage** | **Bone Resorption** |  |
| **1** | **R** | 0 | 0 | 0 | 0 | 0 |
|  | **L** | 0 | 0 | 0 | 0 | 0 |
| **2** | **R** | 0 | 0 | 0 | 0 | 0 |
|  | **L** | 0.5 | 0 | 0 | 0 | 0.5 |
| **3** | **R** | 1 | 0.5 | 0.5 | 0 | 0.5 |
|  | **L** | 1 | 0.5 | 0.5 | 0 | 2 |
| **4** | **R** | 4 | 0.5 | 1 | 0.5 | 6 |
|  | **L** | 4 | 1 | 1 | 1 | 7 |
| **5** | **R** | 1 | 0.5 | 1 | 0 | 2.5 |
|  | **L** | 0 | 0 | 0 | 0 | 0 |
| **6** | **R** | 2 | 0.5 | 0.5 | 0 | 3 |
|  | **L** | 3 | 0.5 | 1 | 0 | 4.5 |
| **7** | **R** | 1 | 0.5 | 0.5 | 0 | 2 |
|  | **L** | 3 | 0.5 | 1 | 0.5 | 5 |
| **8** | **R** | 3 | 1 | 1 | 0.5 | 5.5 |
|  | **L** | 2 | 0.5 | 1 | 0 | 3.5 |
| Mean | | 1.59 | 0.41 | 0.56 | 0.16 | 2.63 |
| SE | | 0.36 | 0.08 | 0.11 | 0.08 | 0.60 |
| ANOVA to Vehicle | | NS | NS | NS | p < 0.05 | NS |
| % inhibition | | 50% | 46% | 57% | 78% | 56% |

**Table S21**. Histopathology Report for Knees after CIA model induction in rats at study day 16 (continued).

| **Treatment Group** | | **Histopathology Scores (from 0=normal to 5=severe)** | | | | **Summed**  **Histopathology**  **Scores** |
| --- | --- | --- | --- | --- | --- | --- |
| **Group 5**  **MTX Veh (PO, QD)**  **MT-SYK-03 (103 mg/kg) (PO, BID)** | | **Inflammation** | **Pannus** | **Cartilage Damage** | **Bone Resorption** |  |
| **1** | **R** | 2 | 0.5 | 0.5 | 0 | 3 |
|  | **L** | 0.5 | 0 | 0.5 | 0 | 1 |
| **2** | **R** | 0.5 | 0 | 0 | 0 | 0.5 |
|  | **L** | 0.5 | 0 | 0.5 | 0 | 1 |
| **3** | **R** | 5 | 2 | 2 | 2 | 11 |
|  | **L** | 4 | 1 | 1 | 1 | 7 |
| **4** | **R** | 5 | 1 | 2 | 1 | 9 |
|  | **L** | 5 | 2 | 2 | 2 | 11 |
| **5** | **R** | 0.5 | 0 | 0 | 0 | 0.5 |
|  | **L** | 0.5 | 0 | 0 | 0 | 0.5 |
| **6** | **R** | 4 | 1 | 1 | 1 | 7 |
|  | **L** | 5 | 1 | 2 | 1 | 9 |
| **7** | **R** | 4 | 1 | 2 | 1 | 8 |
|  | **L** | 3 | 0.5 | 1 | 0.5 | 5 |
| **8** | **R** | 3 | 0.5 | 1 | 0.5 | 5 |
|  | **L** | 2 | 1 | 1 | 0.5 | 4.5 |
| Mean | | 2.78 | 0.72 | 1.03 | 0.66 | 5.19 |
| SE | | 0.46 | 0.16 | 0.19 | 0.17 | 0.95 |
| ANOVA to Vehicle | | NS | NS | NS | NS | NS |
| % inhibition | | 13% | 4% | 21% | 9% | 13% |

**Table S21**. Histopathology Report for Knees after CIA model induction in rats at study day 16 (continued).

| **Treatment Group** | | **Histopathology Scores (from 0=normal to 5=severe)** | | | | **Summed**  **Histopathology**  **Scores** |
| --- | --- | --- | --- | --- | --- | --- |
| **Group 6**  **MT-SYK-03 (103 mg/kg) (PO, BID)**  **MTX (0.075 mg/kg) (PO, QD)** | | **Inflammation** | **Pannus** | **Cartilage Damage** | **Bone Resorption** |  |
| **1** | **R** | 0 | 0 | 0 | 0 | 0 |
|  | **L** | 0.5 | 0 | 0.5 | 0 | 1 |
| **2** | **R** | 0.5 | 0 | 0 | 0 | 0.5 |
|  | **L** | 0.5 | 0 | 0 | 0 | 0.5 |
| **3** | **R** | 0.5 | 0 | 0 | 0 | 0.5 |
|  | **L** | 0.5 | 0 | 0 | 0 | 0.5 |
| **4** | **R** | 0.5 | 0 | 0.5 | 0 | 1 |
|  | **L** | 0.5 | 0 | 0.5 | 0 | 1 |
| **5** | **R** | 0.5 | 0 | 0.5 | 0 | 1 |
|  | **L** | 0.5 | 0 | 0 | 0 | 0.5 |
| **6** | **R** | 0 | 0 | 0 | 0 | 0 |
|  | **L** | 0 | 0 | 0 | 0 | 0 |
| **7** | **R** | 4 | 1 | 2 | 1 | 8 |
|  | **L** | 0.5 | 0 | 0.5 | 0 | 1 |
| **8** | **R** | 0.5 | 0 | 0.5 | 0 | 1 |
|  | **L** | 0 | 0 | 0 | 0 | 0 |
| Mean | | 0.59 | 0.06 | 0.31 | 0.06 | 1.03 |
| SE | | 0.23 | 0.06 | 0.13 | 0.06 | 0.48 |
| ANOVA to Vehicle | | p < 0.05 | p < 0.05 | p < 0.05 | p < 0.05 | p < 0.05 |
| % inhibition | | 81% | 92% | 76% | 91% | 83% |

**Table S22.** The increase in the volume of the treated paws after subplantar CFA injection and intragastric drug administration on days 14–28.

| **Compound** | **The increase in the volume of the paws relative to the initial level, %** | | | | |
| --- | --- | --- | --- | --- | --- |
|  | **14 days** | **18 days** | **21 days** | **25 days** | **28 days** |
| Intact, n = 10 | 0.84 ± 0.28 | 1.14 ± 0.25 | 2.48 ± 0.5 | 3.17 ± 0.43 | 4.85 ± 0.48 |
| Control, n = 10 | 85.02 ± 7.54 & | 85.72 ± 7.57 & | 92.56 ± 6.32 & | 94.57 ± 6.05 & | 100.2 ± 6.19 & |
| Diclofenac, 8 mg/kg QD | 85.1 ± 8.01 & n = 10 | 90.16 ± 9.34 & n = 10 | 89.64 ± 9.99 & n = 9 | 88.04 ± 12.52 &. n = 8 | 92.37 ± 13.78 & n=8 |
| Methotrexate, 0.2 mg/kg QD | 86.76 ± 6.08 & n=10 | 98.21 ± 6.72 & n=10 | 91.28 ± 4.42 & n=10 | 87.99 ± 5.83 & n=9 | 87.92 ± 6.49 & n=8 |
| Methotrexate, 0.5 mg/kg QD | 75.07 ± 8.31 & n=10 | 89.12 ± 6.16 & n=10 | 81.38 ± 5.74 & n=10 | 67 ± 8.92& # n=8 | 38.31 ± 13.28 # n=3# |
| MT-SYK-03, 52 mg/kg BID, n = 10 | 77.6 ± 4.53 & | 85.72 ± 3.7 & | 88.24 ± 4.4 & | 94.75 ± 7.18 & | 93.88 ± 6.77 & |
| MT-SYK-03, 103 mg/kg BID, n = 10 | 76.18 ± 7.73 & | 85.98 ± 7.51 & | 86.55 ± 8.13 & | 86.47 ± 7.89 & | 89.02 ± 7.37 & |
| MT-SYK-03, 207 mg/kg QD, n = 10 | 81.54 ± 6.88 & | 82.92 ± 6.64 & | 83.22 ± 6.53 & | 88.58 ± 7.8 & | 93.67 ± 9.08 & |
| Methotrexate, 0.2 mg/kg QD +  MT-SYK-03, 52 mg/kg BID | 83.68 ± 5.49 & n=10 | 84.69 ± 5.74 & n=10 | 80 ± 6.15 & n=10 | 68.67 ± 6.16& # n=10 | 68.48 ± 8.68 &# n = 6 |
| Methotrexate, 0.2 mg/kg QD +  MT-SYK-03, 103 mg/kg BID | 99.27 ± 5.74 & n=10 | 96.79 ± 3.68 & n=10 | 85.22 ± 5.54 & n=10 | 74.99 ± 5.11 &# n=10 | 64.5 ± 5.12 &# n = 5 |
| Methotrexate, 0.2 mg/kg QD +  MT-SYK-03, 207 mg/kg QD | 68.3 ± 6.66 & n=10 | 85.62 ± 5.99 & n=10 | 76.52 ± 4.96 & n=10 | 72 ± 5.41 &# n=10 | 71.98 ± 5.91 &# n = 9 |

& – statistically significant compared with intact (p < 0.05); # – statistically significant compared with control (р < 0.05).

**Table S23.** Animal death after subplantar administration of CFA and intragastric administration of test compound to rats on the 14-28th days of the study.

| **Compound** | **Animal death, %** | | | | |
| --- | --- | --- | --- | --- | --- |
|  | **14 days** | **18 days** | **21 days** | **25 days** | **28 days** |
| Intact | 0 | 0 | 0 | 0 | 0 |
| Control | 0 | 0 | 0 | 0 | 0 |
| Diclofenac, 8 mg/kg QD | 0 | 0 | 10 | 20 | 20 |
| Methotrexate, 0.2 mg/kg QD | 0 | 0 | 0 | 10 | 20 |
| Methotrexate, 0.5 mg/kg QD | 0 | 0 | 0 | 20 | 70 |
| MT-SYK-03, 52 mg/kg BID | 0 | 0 | 0 | 0 | 0 |
| MT-SYK-03, 103 mg/kg BID | 0 | 0 | 0 | 0 | 0 |
| MT-SYK-03, 207 mg/kg QD | 0 | 0 | 0 | 0 | 0 |
| Methotrexate, 0.2 mg/kg QD +  MT-SYK-03, 52 mg/kg BID | 0 | 0 | 0 | 0 | 40 |
| Methotrexate, 0.2 mg/kg QD +  MT-SYK-03, 103 mg/kg BID | 0 | 0 | 0 | 0 | 50 |
| Methotrexate, 0.2 mg/kg QD +  MT-SYK-03, 207 mg/kg QD | 0 | 0 | 0 | 0 | 10 |

**Table S24.** Effects of MT-SYK-03 on hind paws grip strength (% from the initial one) in MIA-induced OA on the 14th, 21st and 28th days of pathology induction (1 and 3 hours after drug administration). & – statistically significant compared with intact (p < 0.05); # – statistically significant compared with the control group (p < 0.05).

| **Treatment group** | **Regimen** | **14 day** | | **21 day** | | **28 day** | |
| --- | --- | --- | --- | --- | --- | --- | --- |
|  |  | 1 hour after admin. | 3 hours after admin. | 1 hour after admin. | 3 hours after admin. | 1 hour after admin. | 3 hours after admin. |
| Intact |  | 105.9 ± 5.3 | 104.1 ± 5.7 | 109.9 ± 9.2 | 116.17 ± 11.77 | 94.9 ± 8.3 | 98.1 ± 10.0 |
| Control |  | 57.0 ± 7.0 & | 58.9 ± 7.0 & | 54.4 ± 11.1 & | 53.73 ± 7.16 & | 53.4 ± 4.2 & | 54.7 ± 3.3 & |
| MT-SYK-03, 100 mg/kg | −1–28 days, QD | 56.3 ± 5.2 & | 58.7 ± 8.2 & | 76.5 ± 4.5 & | 72.69 ± 9.18 | 90.5 ± 14.0 | 88.6 ± 18.5 |
| MT-SYK-03, 500 mg/kg | −1–28 days, QD | 62.8 ± 9.9 | 70.3 ± 7.0 & | 89.6 ± 8.7 | 76.56 ± 4.83 | 86.3 ± 8.3 | 82.3 ± 12.7 |
| MT-SYK-03, 100 mg/kg | 7–28 days, QD | 70.1 ± 10.8 | 58.9 ± 7.8 & | 76.3 ± 6.2 & | 62.56 ± 7.58 | 74.4 ± 13.0 | 58.1 ± 10.0 |
| MT-SYK-03, 500 mg/kg |  | 68.2 ± 11.3 | 78.6 ± 12.1 | 67.7 ± 7.9 & | 68.23 ± 6.84 | 82.8 ± 9.5 **#** | 79.8 ± 4.3 |
| Nalgesin, 50 mg/kg | 14–28 days, QD | 66.0 ± 11.6 | 59.6 ± 7.1 & | 83.1 ± 7.6 &**#** | 78.2 ± 5.6 | 88.2 ± 9.5 # | 83.6 ± 9.6 |
| Zoledronic acid, 0.1 mg/kg | 0–28 days, once three days | 60.4 ± 10.4 & | 75.4 ± 7.7 & | 96 ± 6.8 **#** | 84.3 ± 7.5 | 92.8 ± 7.6 **#** | 93.9 ± 15.5 |

**Table S25.** Effects of MT-SYK-03 on mechanical hyperalgesia (% from the initial one) in MIA-induced OA on the 14th, 21st and 28th days of pathology induction (1 and 3 hours after drug administration). & – statistically significant compared with intact (p < 0.05); # – statistically significant compared with the control group (p < 0.05).

| **Treatment group** | **Regimen** | **14 day** | | **21 day** | | **28 day** | |
| --- | --- | --- | --- | --- | --- | --- | --- |
|  |  | 1 hour after admin. | 3 hours after admin. | 1 hour after admin. | 3 hours after admin. | 1 hour after admin. | 3 hours after admin. |
| Intact |  | 103 ± 5.3 | 103.1 ± 4.2 | 110.8 ± 15.2 | 94.8 ± 4 | 104.9 ± 8.3 | 106.11 ± 9.61 |
| Control |  | 66.3 ± 9.7 & | 77.5 ± 11.7 | 58.6 ± 3.9 & | 61.2 ± 8.2 & | 69.1 ± 6.5 & | 72.89 ± 5.24 |
| MT-SYK-03, 100 mg/kg | −1–28 days, QD | 73.5 ± 10.5 & | 63.9 ± 7.5 & | 74.4 ± 3.3 &# | 80.5 ± 10.3 | 65.1 ± 7.4 & | 67.38 ± 9.74 & |
| MT-SYK-03, 500 mg/kg | −1–28 days, QD | 53.8 ± 4.4 & | 52.5 ± 3.3 & | 75.4 ± 6.4 # | 64.7 ± 4.3 & | 53.5 ± 3.8 & | 61.04 ± 1.81 & |
| MT-SYK-03, 100 mg/kg | 7–28 days, QD | 51.9 ± 4.1 & | 50.6 ± 5.6 & | 85.1 ± 5 # | 73.5 ± 6.9 & | 67.7 ± 9.8 & | 71.24 ± 7.99 |
| MT-SYK-03, 500 mg/kg |  | 51.1 ± 5.4 & | 77.5 ± 6.9 & | 56 ± 7.2 & | 58.5 ± 7.1 & | 75 ± 6.1 & | 78.02 ± 9.37 |
| Nalgesin, 50 mg/kg | 14–28 days, QD | 64.5 ± 9.2 & | 69.6 ± 9 & | 90.3 ± 8.5 # | 74.3 ± 5.4 & | 79.6 ± 9.2 | 78.9 ± 5.3 |
| Zoledronic acid, 0.1 mg/kg | 0–28 days, once three days | 68.1 ± 6.3 & | 68.3 ± 3.5 & | 85.6 ± 5.6 **#** | 83.4 ± 5.2 # | 81.8 ± 3.7 & | 84.2 ± 7.4 |

## **Table S26.** The increase in the volume of the non-involved paws after subplantar CFA injection and intragastric drug administration on days 14–28. & – statistically significant compared with intact (p < 0.05); # – statistically significant compared with control (р < 0.05).

| **Compound** | **The increase in the volume of the paws relative to the initial level, %** | | | | |
| --- | --- | --- | --- | --- | --- |
|  | **14 days** | **18 days** | **21 days** | **25 days** | **28 days** |
| Intact | 1.73±0.56 | 3.46±0.94 | 4.26±0.75 | 5.66±1 | 7.18±1.12 |
| Control | 13.63±1.93& | 21.82±1.81& | 25.57±2.04& | 26.94±2.2& | 28.26±2.15& |
| Diclofenac, 8 mg/kg QD | 14.28±0.98& | 22.01±2.42& | 22.85±2.82& | 19.99±2.76& | 20.8±3.06& |
| MTX, 0.2 mg/kg QD | 11.11±2.13& | 21.49±4.13& | 20.14±4.05& | 21.82±4.51& | 18.98±3.81& |
| MTX, 0.5 mg/kg QD | 11.95±2.98& | 17.5±3.65& | 18.74±3.38& | 19.88±2.94& | **12.98±5.04 #** |
| MT-SYK-03, 52 mg/kg BID | 13.93±1.83& | 22.55±2.93& | 23.66±3.25& | 24.39±2.8& | 25.25±2.86& |
| MT-SYK-03, 103 mg/kg BID | 11.22±1.26& | 17.42±1.16& | 20.38±2.11& | 22.35±2.28& | 21.41±2.05&# |
| MT-SYK-03, 207 mg/kg QD | 14.3±1.82& | 19.97±1.89& | 21.15±1.89& | 22.51±1.61& | **21.94±1.37&#** |
| MTX, 0.2 mg/kg QD + MT-SYK-03, 52 mg/kg BID | 11.99±2.93& | 16.56±2.15& | **14.87±2.63&#** | **13.64±2.6&#** | **15.46±3.34 #** |
| MTX, 0.2 mg/kg QD + MT-SYK-03, 103 mg/kg BID | 11.63±2.66& | 25.49±4.01& | **15.97±2.53&#** | **13.05±2.08&#** | **15.94±2.64&#** |
| MTX, 0.2 mg/kg QD + MT-SYK-03, 207 mg/kg QD | 11.76±2.59& | 18.76±2.32& | **18.52±1.97&#** | **18.71±2.15&#** | **19.51±3.03&#** |

## **Table S27.** Effects of MT-SYK-03 on mechanical allodynia pain threshold (% from the initial one) in MIA-induced OA on the 14th, 21st and 28th days of pathology induction (1 and 3 hours after drug administration). * – statistically significant compared with intact (p < 0.05); & – statistically significant compared with the control group (p < 0.05).

| **Treatment group** | **Regimen** | **14 day** | | **21 day** | | **28 day** | |
| --- | --- | --- | --- | --- | --- | --- | --- |
|  |  | 1 hour after admin. | 3 hours after admin. | 1 hour after admin. | 3 hours after admin. | 1 hour after admin. | 3 hours after admin. |
| Intact |  | 100.6±4.7 | 121.4±19.5 | 117.4±8.3 | 112.3±6.9 | 124.9±5.6 | 120.2±13 |
| Control |  | 59.9±8* | 61.1±10.3* | 58.2±4.8* | 60.7±8.2* | 60±5.7* | 67.9±12.4* |
| MT-SYK-03, 100 mg/kg | −1–28 days, QD | **136.3±22.5 &** | **136.7±29.2 &** | **107.7±12.8 &** | **95.5±4.7 &** | **135.8±27.6 &** | 110.3±16.3 |
| MT-SYK-03, 500 mg/kg | −1–28 days, QD | **113.9±9.7 &** | **111.1±17.7 &** | **105.8±13 &** | **98.1±5.7 &** | **106.7±12 &** | **99.2±5.5 &** |
| MT-SYK-03, 100 mg/kg | 7–28 days, QD | 70.2±7.7* | 78.7±10.6 | 75.3±15.4* | 73.1±7.9* | 61.7±5.8* | 78.3±8* |
| MT-SYK-03, 500 mg/kg |  | 75.7±3.6* | 82.7±12.8 | 74.5±13.9* | 78.9±6.8* | **198.4±40.1 &** | **161.1±30.5 &** |
| Nalgesin, 50 mg/kg | 14–28 days, QD | 82.2±16 | 120.8±33.7 | **101.7±18.2 &** | **94.7±9.7 &** | 96.2±19.3 | 111.3±21.4 |
| Zoledronic acid, 0.1 mg/kg | 0–28 days, once three days | **100.6±8 &** | **101.3±12.3 &** | **108.2±7.9 &** | **106.8±6.5 &** | **112.4±8.6 &** | **126.6±15.4 &** |
